# A porphyrin-based molecular cage guided by designed local-electric field is highly selective and efficient†

**DOI:** 10.1039/d3sc01720f

**Published:** 2023-09-04

**Authors:** Shakir Ali Siddiqui, Sason Shaik, Surajit Kalita, Kshatresh Dutta Dubey

**Affiliations:** a Department of Chemistry, School of Natural Sciences, Shiv Nadar Institution of Eminence Delhi-NCR India kshatresh.dubey@snu.edu.in; b Institute of Chemistry, The Hebrew University of Jerusalem Israel Jerusalem Israel sason@yfaat.ch.huji.ac.il

## Abstract

The present work outlines a general methodology for designing efficient catalytic machineries that can easily be tweaked to meet the demands of the target reactions. This work utilizes a principle of the designed local electric field (LEF) as the driver for an efficient catalyst. It is demonstrated that by tweaking the LEF, we can catalyze the desired hydroxylation products with enantioselectivity that can be changed at will. Using computation tools, we caged a synthetic analog of heme porphyrin (HM1) and investigated the pharmaceutically relevant conversion of tetralin to tetralol, inside the modified supramolecular cage. The QM/MM calculations demonstrate a resulting catalytic efficiency with virtually absolute *R-*selectivity for the tetralin hydroxylation. Our calculations show that the LEF of the supramolecular cage and HM1 exert a strong electric field along the Fe–O reaction axis, which is the main driving force for enhanced reactivity. At the same time, the supramolecular cage applies a lateral LEF that regulates the enantioselectivity. We further demonstrate that swapping the charged/polar substitution in the supramolecular cage switches the lateral LEF which changes the enantioselectivity of hydroxylation from *R* to *S*.

## Abstract 2

The present work outlines a general methodology for designing efficient catalytic machineries that can easily be tweaked to meet the demands of the target reactions.

## Graphical abstract

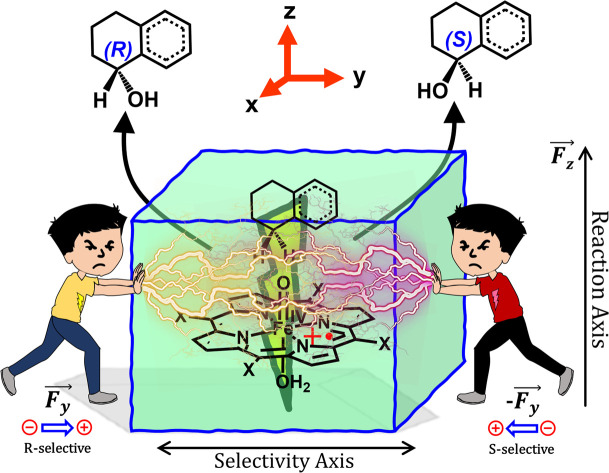

## 1. Introduction

We outline here a general methodology for designing efficient catalysts that can easily be tweaked to meet the demands of the target reactions. The work utilizes computational design principles^1–5^ towards this goal. The principles are then applied to design a supramolecular cage that possesses an efficient scaffold, which can be easily modified to catalyze the formation of desired hydroxylation products with enantioselectivity at will.

Still though, there are two major caveats in such catalyst design; (a) the choice of scaffold for designing, and (b) the codification of the descriptor that affects the catalysis.^6–8^ For the initial scaffold, enzymes could be an alternative; however, the low thermal stabilities^9,10^ and low tolerance towards the organic solvents^11,12^ remain a major challenge for an enzymatic scaffold. In addition, the enzymes are giant macromolecular systems that are very selective for their native reactions. As such, evolving an enzyme for a new activity requires a large random mutation in the protein matrix.^13–15^ However, supramolecular complexes such as organic cages could be a better alternative as they possess a relatively smaller size and an active site architecture analogous to the enzymes.^16–19^ Herein, we show a supramolecular cage could be used as an efficient scaffold that can easily be tweaked for the desired product.

The electrostatic field of the supramolecular cage is an ideal choice for a catalytic effector^19^ and could resolve (b) *i.e.*, the codification of the descriptor. It is established that the preorganization of the electrostatic field is responsible for catalysis and has been well established for the enzyme catalysis.^20^ Indeed, recent studies by several groups have shown that the local electric fields (LEFs) of enzymes and/or solvents constitute the main catalytic elements for several reactions.^21–27^ This fact is also supported by several recent studies where the application of oriented external electric field (OEEF)^28–30^ along the “reaction axis” significantly enhances the rate of reactions and controls their product selectivity.^31–38^ As such, the LEF along the reaction axis can be used as an effector of the catalytic activity^26^ in our target cages. Herein we demonstrate that a methodological design of LEF in a supramolecular cage brings about the desired reactivity and selectivity of C–H hydroxylation reactions.

### 1.1 The model system

**Fig. 1:**
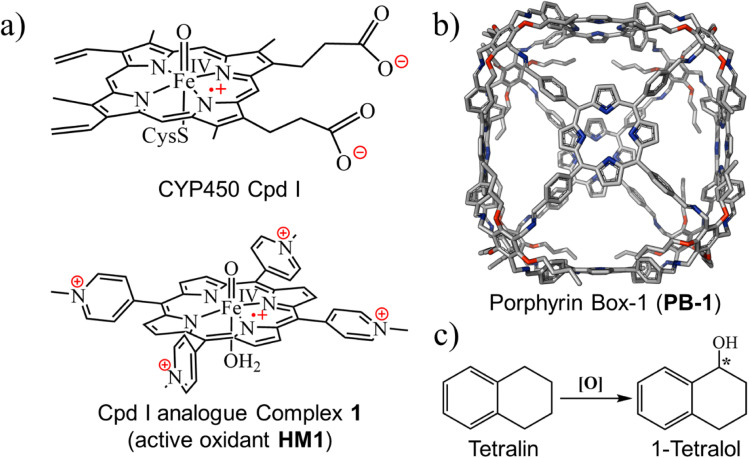
(a) The active oxidant Cpd I in the catalytic cycle of P450s and its analogue complex 1, here referred as HM1. (b) Porphyrin box-1 (PB-1), initial skeleton used in the study. Hydrogens are omitted for clarity. (c) Chemical structure of tetralin and tetralol.

Our choice of initial catalyst is a synthetic porphyrin derivative, HM1 in Fig. 1a, which was developed by Groves *et al.*^39^ and demonstrated to enhance hydrogen abstraction reactions in aqueous solutions. The rate-enhancement capability of HM1 was later shown to originate in the LEF of the charged peripheral substituents.^21^

For the supramolecular scaffold, we selected the porous organic cage (Fig. 1b), which was synthesized by Kim *et al.*, and named porphyrin box, PB-1.^16^ As was shown by Kim *et al.*, PB-1 possesses exceptional chemical stability and applicability; including selective gas sorption, encapsulation of guest molecules, *etc.* Importantly, such porous organic cages were reported to preserve their stability even after post-synthetic modification (PSM)^40–42^ of some functional groups of such cages. Therefore, we deem this supramolecular scaffold to be an ideal starting platform that can be selectively modified (see ESI, Fig. S1 and S2†) for tailoring the LEF along the reaction axis of HM1, as well as for encapsulating target substrates for oxidation.

As a substrate that can be used to explore reactivity and selectivity, we chose the conversion of tetralin to tetralol (Fig. 1c), which involves both C–H hydroxylation and enantioselectivity of the product. Thus, (*R*)-1-tetralol is extensively used to treat several disorders such as obsessive-compulsive disorder, post-traumatic stress, premenstrual dysphoric disorder, and social anxiety.^43–45^ Can the chosen cage produce (*R*)-1-tetralol? Can it produce, on demands, also (*S*)-1-tetralol?

As shown in the foregoing sections, we can tailor the magnitude of the LEF as well as its specific orientation and produce a supramolecular cage that enhances reactivity and enantioselectivity.

## 2. Computational details

In the present study, we used MD simulations for the entrapment, conformational and binding stabilities of TLN & HM1 inside the cage, QM/MM calculations for the mechanism and electronic structure investigations, quantification of the local electric field and QM-only DFT calculations to study the effect of the oriented external electric field (OEEF) onto the reaction axis. The details of each method are described below.

### 2.1 System preparation

**Fig. 2:**
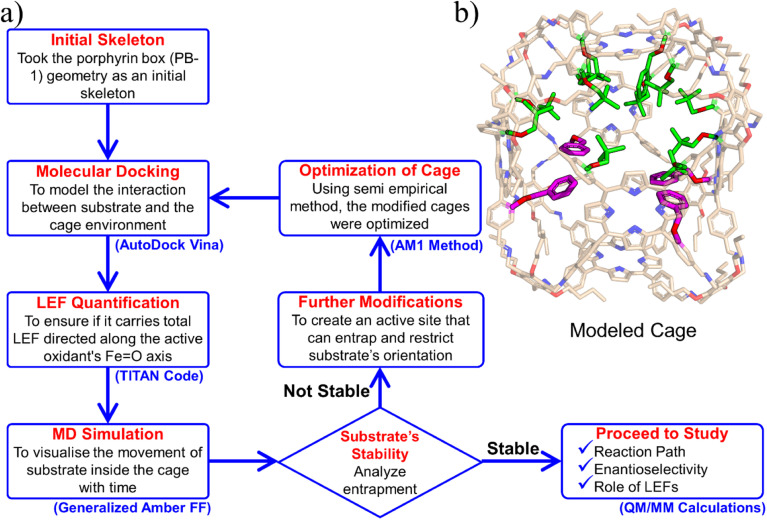
(a) A flowchart of the protocol to engineer the porphyrin box for the substrate and active oxidant HM1. (b) Modeled cage, here modifications are highlighted in green.

We chose the porous organic cage, created by Kim *et al.*^16^ and known as porphyrin box PB-1, for the initial supramolecular scaffold, and modelled it for entrapment of substrate and stability of HM1 using the flowchart in Fig. 2. The detailed description of the modelling procedure is discussed in Text S1 of the ESI.† The PB-1 was optimized with AM1 semi-empirical theory.^46^ The parameters for PB-1 cage were generated using the antechamber module of Amber20 for GAFF2 parameters^47^ for a QM-optimized geometry at the AM1 level of theory. The parameters for oxidant HM1 were prepared by the MCPB.py program, a python-based metal parameter builder used to generate a forcefield for the metal center of the protein, employing the bonded model approach.^48^ Again, the partial atomic charges and missing parameters for tetralin (TLN) substrate were obtained from the RESP charge fitting method for the QM-optimized geometry at the HF/6-31G* level of theory.^49,50^ After docking the HM1 & TLN inside the so-created cages, all the complexes were solvated in a rectangular box of CHCl_3_ solvent molecules with a 10 Å cutoff from the supramolecular cage boundary using the CHCl3BOX using leap module. A suitable quantity of Cl^−^ ions were supplied to balance out the system's overall charge in each case.

### 2.2. MD simulations

The various systems were minimized in two phases after the complexes were initially built up to get rid of the poor connections. In step 1, only solvent molecules were minimized using the conjugate gradient minimizer after 5000 steps of the steepest descent. Using the same minimizer, as in step 1, all of the complexes were minimized in step 2 without any constraints.

All the systems were then gently equilibrated from 10 to 300 K under the NVT ensemble for 50 ps with a weak restraint of 5 kcal mol^−1^ Å^−2^. Subsequently, a density equilibrium was performed using the NPT ensemble at a targeted temperature of 300 K and pressure of 1 atm for 1 ns using the Langevin thermostat^52^ and the Berendsen barostat,^53^ with a weak restraint of 1 kcal mol^−1^ Å^−2^. Following the removal of all restraints, the systems were further equilibrated for 3 ns, and then 100 ns of production simulations for each system were run and the convergence of the simulation is checked. Furthermore, these procedures were replicated three more times. The covalent bonds that contain hydrogens were constrained using the SHAKE algorithm,^51^ and a particle mesh Ewald (PME) method^52^ was utilized to treat long-range electrostatic interactions during all the MD simulations. The entire simulation was run with an integration step of 2 fs. The Amber20 package's GPU version was used to run all of the MD simulations.^53^

### 2.3. QM/MM calculations

The reaction mechanism and the electronic structure were investigated using QM/MM calculations for the snapshot from the MD trajectory. The QM regions included the oxidant HM1 and the substrate tetralin (TLN), for which the coordinates for the QM region for QM/MM optimized geometry can be found in this ESI.† The entire supramolecular modeled cage and solvent molecules within 8 Å of HM1 were included in the ‘active region’ of the QM/MM calculations. The atoms in the ‘active region’ interact with the QM atoms through electrostatic and van der Waals interactions and the corresponding polarization effects were considered in the subsequent QM/MM calculations. All the QM/MM calculations were performed using ChemShell,^54,55^ by combining Turbomole^56,57^ for the QM part and DL_POLY^58^ for the MM part. The MM region was described using the Amber force field generated for the cage, and the electronic embedding scheme was used to account for the polarizing effect of the enzyme environment on the QM region.

During QM/MM geometry optimizations, the QM region was computed using the hybrid UB3LYP functional^59^ with the def2-SVP basis set. All of the QM/MM transition states (TSs) were located by relaxed potential energy surface (PES) scans followed by full TS optimizations using the P-RFO optimizer^60^ implemented in the HDLC code. The results were further validated with single-point calculations at a higher basis set, def2-TZVP. All calculations were performed for the doublet and sextet states since recent studies showed that the pristine oxidant HM1 displays two-state reactivity (doublet and sextet spins) in gas phase.^21,61^

### 2.4. Quantification of the local electric fields (LEFs) and electrostatic stabilization energy

The TITAN and TUPÃ codes were used to quantify the local electric fields (LEFs) present in the systems.^22,62,63^ Using the TUPÃ code, we quantified the evolution of LEFs for solvated cage, oxidant HM1 & entire system from whole MD simulation trajectory at 10 ps time intervals, along the reaction axis, (*z*-axis, Fe=O) as well as the selectivity axis (*y*-axis). The solvent molecules present in the 3 Å vicinity of HM1 were included. The electric fields along the MD trajectory were measured using the point charge distribution included in the parameter topology file.

For the QM/MM optimized RS and TS geometries, the LEF along both the axes were quantified using in-house TITAN code. The quantification included the charges of methyl pyridinium cationic periphery of HM1, entire cage and the solvent molecule near 3 Å of HM1. Dipole moments were calculated for H_2_O–Fe=O, porphyrin and TLN substrate. Then interaction/stabilization energy were calculated using the following relationship:^30^
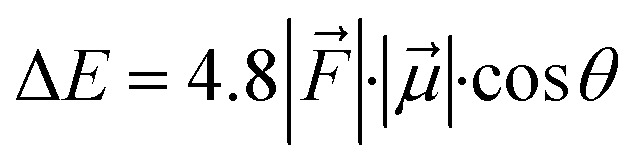
Here, Δ*E* is stabilization energy in kcal mol^−1^, 
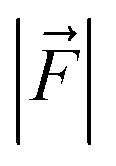
 is the LEF intensity in V Å^−1^, the 
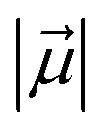
 is the magnitude of dipole moment in Debye and *θ* is the angle between LEF and dipole moment vectors. For electric field and dipole moments, GAUSSIAN convention is used.^30,64^

Furthermore, QM-only single point calculations were performed with and without oriented external electric fields (OEEFs) for the stripped RS and TS geometries. For frequency calculations, we used the hybrid UB3LYP/def2-SVP level of theory^59^ and single-point calculations at a higher basis set, def2-TZVP. These effects were studied using Gaussian09 program.^65^

### 2.5. Adaptive steered molecular dynamics (ASMD) simulations

For efficient catalysis the entry and exit of the substrate/product is crucial, and therefore, we determined the route and feasibility of substrate entry and product escape in the designed cage, using adaptive steered molecular dynamics (ASMD) simulations.^53^ In so doing, we applied an external force in a predetermined direction and performed 20 iterative simulations of ASMD by using the end-to-end distance between the iron center of Fe=O and the ‘C’ of the substrate/product as our reaction coordinate. The adaptive steering was continued along the reaction coordinate from 3 to 23 Å in 10 stages until the substrate/product was entirely outside of the cage. We assigned a spring constant of 10 kcal mol^−1^ Å^−2^, and used a velocity of 0.5 Å ns^−1^ for simulations of a total duration of 800 ns. Finally, we calculated the potential of mean force (PMF), from entire 800 ns of simulation which characterizes the free energy change along a reaction coordinate during different intermediate stages of a product exit using the Jarzynski's equality^66^ -

1 〈exp(−*βW*)〉 = exp(−*β*Δ*G*)

In eqn (1), 〈〉 denotes the ensemble average, *β*=(*k*_B_*T*)^−1^ (*k*_B_ is Boltzmann constant and *T* is temperature), *W* is the work done on the system during a non-equilibrium process, and Δ*G* is the difference in free energy between two equilibrium states of the system.

## 3. Results & discussion

### 3.1 Encapsulation of the active oxidant and the substrate inside the modeled cage

Initially, we tailored PB-1 to better fit HM1 using a protocol shown in Fig. 2a. Since, the active oxidant HM1 contains four peripheral pyridinium groups, we anticipated that it could be stabilized in the cage with the aid of π–π stacking interactions, and therefore, we introduced phenyl rings inside the walls of PB-1. The initial substitution for entrapment of only HM1 can be found in the ESI (Fig. S1).† The simulation of the modified cage and its resident HM1 substantiates our reasoning that the stability of the complex was mainly driven by π–π stacking interactions. Subsequently, we docked the tetralin substrate in the so modified cage. After a few rounds of subsequent modifications that preserve the stability of HM1 and the binding of the substrate during simulations (see ESI, Fig. S2 and S5–S7†), we eventually achieved our final ‘modeled cage’ is shown in Fig. 2b. Here, the violet color shows modifications to stabilize HM1, while green represents the requisite modifications that stabilize the substrate binding.

#### 3.1.1 The stability of the substrate and oxidant inside the cage

**Fig. 3:**
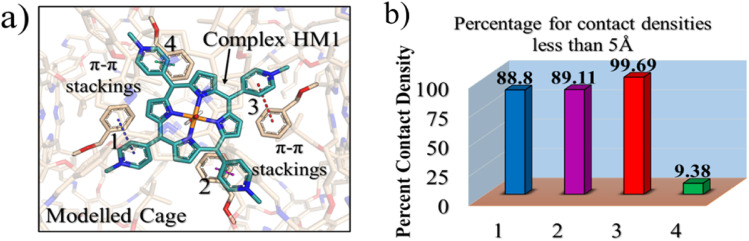
(a) A representative MD snapshot illustrating the π–π stacking interactions between the inserted HM1 and the introduced phenyl rings in interior walls of the modeled cage. 1, 2, 3 and 4 represent the four different π–π stacking interactions, shown in blue, magenta, red and green, respectively. (b) The percentage for contact densities between HM1 and four phenyl substitutions.

The MD simulations of the substrate and the oxidant in the solvated cage show that three phenyl rings of the cage strongly interact with the three peripheral pyridinium substituents of the porphyrin HM1 and stabilize it (see Fig. 3a). The contact density (Fig. 3b) further validates the strong π–π stacking interactions with the modified cage and HM1. Note that three interactions out of four are occupied for more than 88% of the time below 5 Å distance during the simulation, which shows strong π–π interactions (for more, see ESI, Fig. S15†). Additionally, the root mean square deviation (RMSD) of the modified cage in the presence of HM1 and the substrate during the molecular dynamics (MD) simulations is just ∼1 Å, which strongly validates the stability of the host–guest model (see RMSD in Fig. S4†).

#### 3.1.2 The entry of the substrate and exit of the product

Since, the entry of the substrate and exit of the product is crucial for the catalysis, we investigated the entry/exit of the substrate/product *via* adaptive steered molecular dynamics (ASMD) simulation and calculated the potential of mean force (PMF). The total work (PMF) implies that the substrate can easily access the active oxidant inside the modeled cage, while the hydroxylated product can escape and leave the space (see ESI, Text S4† for more details).

### 3.2 Testing the role of the LEF on reactivity and selectivity

This section demonstrates that the unusual impact of the cage on reactivity and enantioselectivity originates in the combined LEF of HM1 and the container PB1. This is done by initially evaluating the combined LEF effects on the RS and TS species along the reaction coordinate. Subsequently, we investigate the role of the LEF in determining the special electronic structures of these species. This was complemented by determining the LEF role on the enantioselectivity of the reaction. And finally, by showing that we can get enantioselectivity at will, by simply flipping the direction of the lateral LEF.

#### 3.2.1 The LEF of the complex

**Fig. 4:**
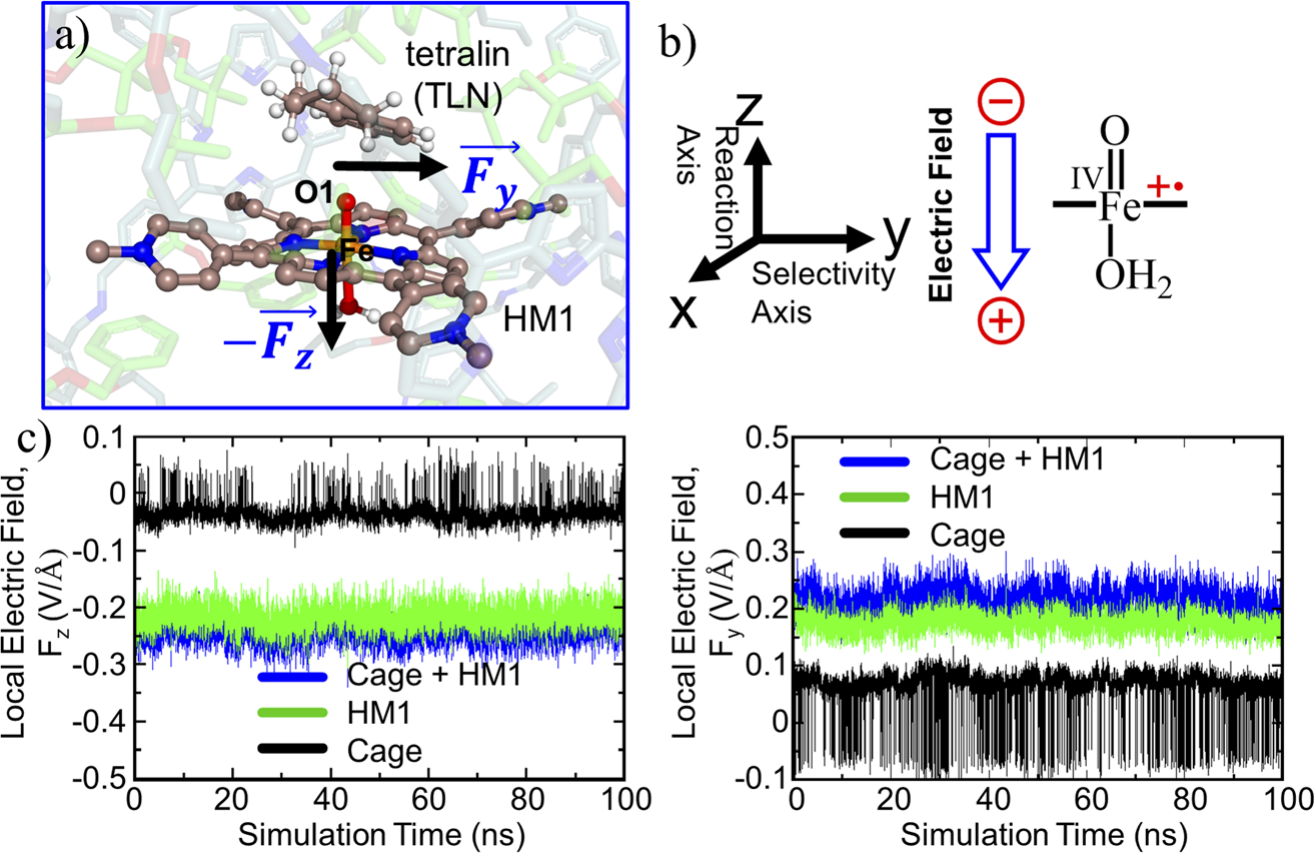
(a) A representative MD snapshot highlighting the reactive complex inside the cage. (b) Reaction axis & electric field direction, GAUSSIAN convention is used. (c) The local electric field (LEF) along *y*- and *z*-axis with evolution of time.

Let us quantify the LEF of the complex (solvated PB1 + HM1) along the reaction axis (*z*-axis) and the lateral direction (*y*-axis). Such a 2-dimensional (2D) electric field was demonstrated in the past to bring about reactivity enhancement as well as enantioselectivity.^33^ Thus, we expect that the LEF along the *z*-axis affects reactivity while along the *y*-axis, it affects the enantioselectivity. Our analysis in Fig. 4 shows the existence of a significant LEF along the Fe–O, *i.e.*, *z*-axis. This LEF(*Z*) is a combination of the LEFs of the solvated cage and the HM1, where the solvated cage contributes ∼0.07 V Å^−1^ and HM1 contributes ∼0.23 V Å^−1^ to the total LEF of ∼0.30 V Å^−1^. Furthermore, the cage contributes also a significant LEF(Y) in the *Y*-direction of −0.10 V Å^−1^. As such, the LEF of the cage and HM1 are expected to play a significant role in the reactivity and enantioselectivity of the oxidation reaction.

**Fig. 5:**
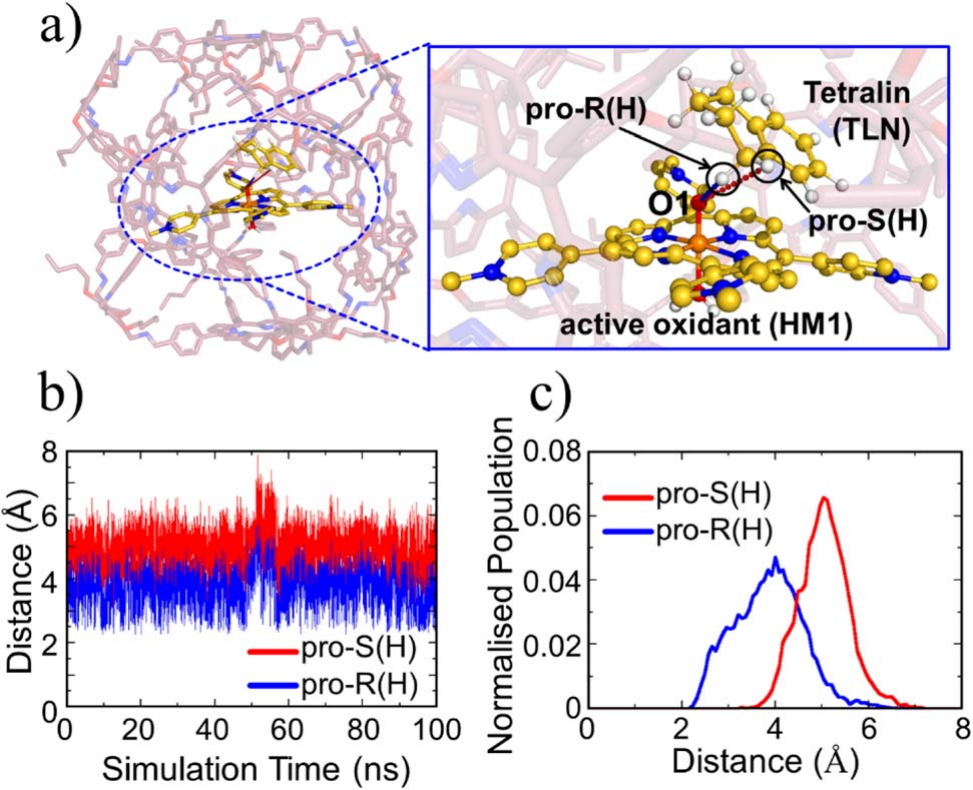
(a) A representative MD snapshot unveiling the pro-*R* and pro-*S* hydrogens of the substrate tetralin (TLN). (b) Distance plots between these hydrogens and O1 atom of the oxidant (HM1). (c) The Boltzmann population of the pro-*R* and pro-*S* hydrogens distances (from the oxo atom of Fe=O) over the entire 100 ns MD simulation.

While oxidant HM1 is known to be intrinsically reactive in H-atom-transfer (HAT) reactions,^21,39,61^ the enantioselectivity has not been explored. As can be seen from Fig. 5a which represents a snapshot from 100 ns MD simulation, the substrate occupies a very a close position to the oxidant, and both pro-*R* and pro-*S* hydrogen atoms are also in the proximity of the oxo-iron moiety of HM1. The proximities of these hydrogen atoms are also persistent throughout the simulations which can be verified by the distance *vs.* time plot (Fig. 5b) and the plot of distance *vs.* population (Fig. 5c). At the same time, it is apparent that the pro-*R* hydrogen is closer than the pro-*S* one. As mentioned earlier, hydroxylation of tetralin at pro-*R* position has medicinal importance.^43^ It is noteworthy that our modeled system gives a clear preference for pro-*R* selectivity, and is thus of industrial relevance.

#### 3.2.2 QM/MM calculations during the reaction course

**Fig. 6:**
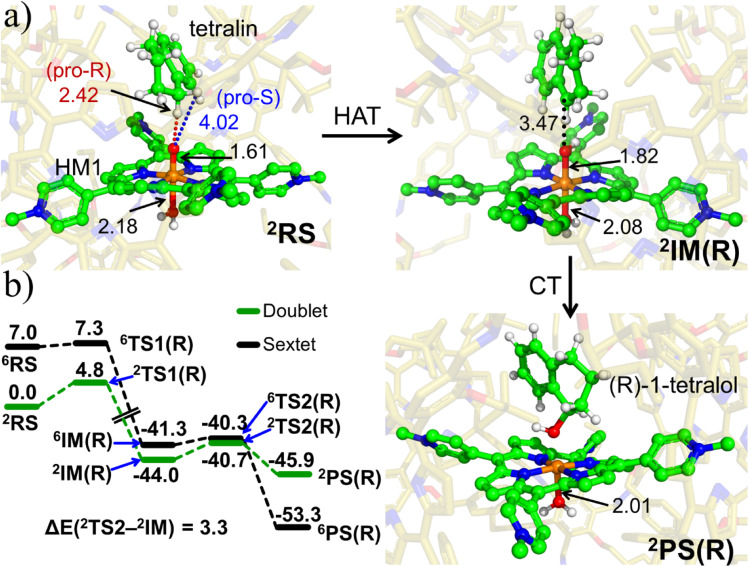
(a) The key geometric data as well as QM/MM optimized geometries of ^2^RS, ^2^IM(R) and ^2^PC(R). The optimized geometries of ^2^TS1(*R*), ^2^TS2(*R*) and for the corresponding sextet state species can be found in ESI, Fig. S9 & S10.† (b) The complete ZPE corrected QM/MM/UB3LYP/def2-TZVP reaction profile diagram for the *R*-enantioselective C–H hydroxylation of tetralin into (*R*)-1-tetralol by active oxidant HM1 entrapped inside the modelled cage. All the calculations have been performed for both doublet and sextet states. Distances are in Å, and energies are reported in kcal mol^−1^ relative to RS. Note that RS, TS and PS, here, stand for reactant state, transition state and product state.

To validate the kinetic feasibility of the *R*-enantioselective reaction, we performed QM/MM calculations, using the most populated snapshot based on Boltzmann population distribution‡‡A choice which is statistically more relevant relative to stochastically chosen snapshots., followed by potential energy scan along the H-abstraction pathway. Furthermore, since recent investigations^21^ revealed that the pristine oxidant HM1 exhibits two-state reactivity (doublet and sextet spins) in its gas phase reaction with methane, we investigate the reactivity of the two spin states. Our QM/MM calculations, in Fig. 6a and b, show that the ground state of HM1 is the doublet state. The reaction barrier is also lower than in the sextet state and the reaction is hence spin-selective (>99%). Clearly therefore the box modifies the intrinsic properties of the captive HM1.

Furthermore, it is seen that the H-transfer energy barrier is merely 4.8 kcal mol^−1^, and the process is preferred for the pro-*R* H-abstraction. Notably, the reaction is mediated by IM1, which involves a tetralinyl cation and Fe(iii)–OH^−^ heme species. Subsequently, the substrate cation attacks the Fe–OH bond and generates the *R*-tetralol product. By comparison, the pro-*S* H-abstraction barrier is 7.81 kcal mol^−1^ (see ESI, Fig. S11 & S12†). Based on the energy barrier difference, the *R*/*S* ratio at 298.15 K, corresponds to 159 : 1 for *R*/*S*-enantioselectivity of the Tetralol (see ESI, Fig. S11†). Clearly the cage modifies both the reactivity and enantioselectivity of HM1. To further substantiate the role of cage, we performed separate calculations by stripping the cage and details for the same are shown in Fig. S13 of the ESI.†

In contrast to the previous study with HM1,^21^ here there is no spin crossover during the H-transfer step, since the reaction prefers to occur almost exclusively on the doublet state surface. Thus, the cage environment perturbs the electronic states and creates a preference for the doublet state. At the same time, the final *R*-alcohol product is most stable in the sextet state, as happens in many reactions of P450.^67^

#### 3.2.3 The mechanism of the oxidation reaction and the role of LEF

**Fig. 7:**
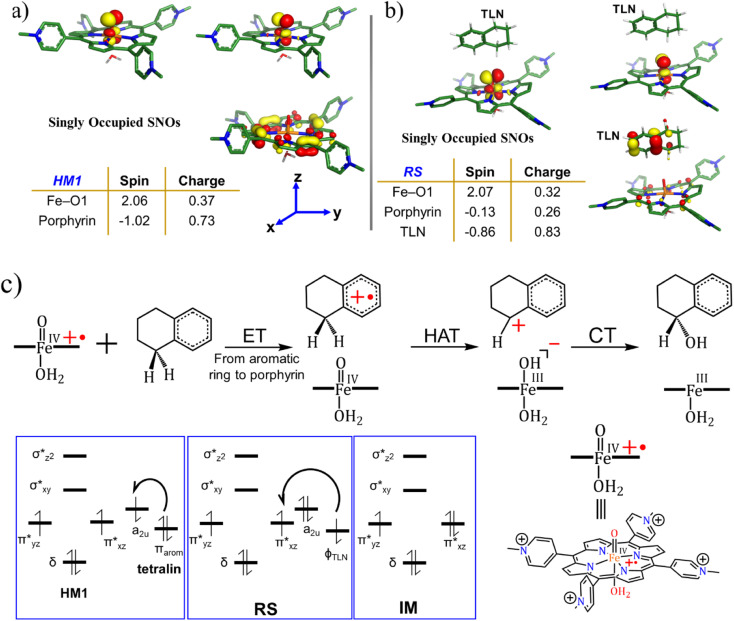
The spin natural orbitals (SNOs), mulliken spin density and charges for (a) lone active oxidant HM1 and (b) reactant state, RS. TLN refers to the substrate tetralin. All the electronic structure calculations were carried out for the doublet ground state of the complex. (c) A complete reaction mechanism along with the orbital occupation during reaction. See ESI† for Mulliken spin density and charges, Table S1.†

To decipher the reaction mechanism, we studied the electronic structures of all stationary species with spin doublet. As can be seen from Fig. 7a, in the free oxidant, the three unpaired electrons are populated on HM1, much the same as in the standard electronic structure of porphyrin radical-cation coupled to a triplet oxo-iron moiety to a total of a doublet spin. However, in the presence of tetralin (TLN) entrapped inside the cage, an electron is transferred from TLN (Fig. 7b) to the singly occupied orbital of the HM1^+^˙ species. This electron transfer occurs due to the LEF of the cage that drives an electron from TLN to HM1. A complete mechanism of the reaction is depicted in Fig. 7c. As can be seen, the reaction begins with a single electron transfer from the electron-rich TLN substrate to HM1, to yield the RS, followed by a hydrogen transfer to the oxo–iron complex, and finally, the TLN-cation rebounds on the hydroxo–iron complex and yields the tetralol product.

**Fig. 8:**
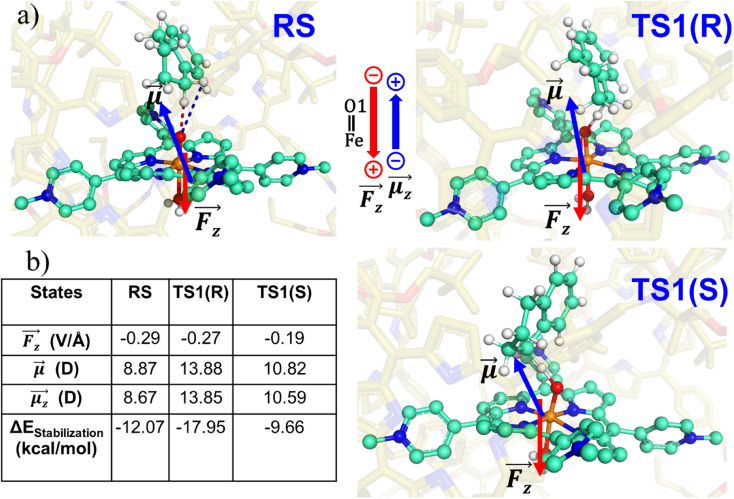
(a) RS, TS1-*R* and TS1-*S* states showing the direction of local electric field (LEF) and dipole moment. (b) Calculated LEF_*z*_, dipole moments, and stabilization energies. Note that we have a GAUSSIAN convention to define the direction of electric fields and dipole moments. Dipole moments have been calculated for H_2_O–Fe=O, porphyrin and TLN.

To substantiate the role of the LEF, we quantify in Fig. 8 the LEF exerted by the cage along the reaction axis using the TITAN program. As we can see, the LEF significantly stabilizes the TS state for the *R-*enantioselectivity compared with the TS for the *S-*enantioselectivity. Herein, the LEF calculation and analysis are more focused on the RS and TS1 state, being the rate-determining step. It plays a crucial role in the overall reaction kinetics because it is the step that significantly impacts the overall reaction rate.

#### 3.2.4 Is the LEF responsible for the initial electron transfer (ET)?

**Fig. 9:**
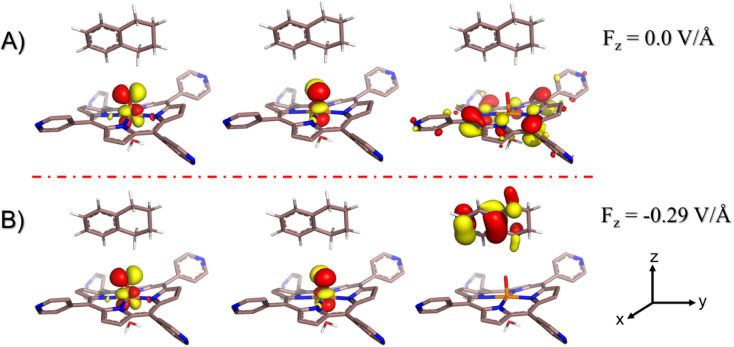
Singly occupied spin natural orbitals (SNOs) for demethylated (uncharged) RS doppelganger; (A) in the absence of any externally oriented electric field, (B) in the presence of the oriented external electric field of intensity −0.29 V Å^−1^ projected along with the F–O axis (negative *z*-axis) of the same order of magnitude exerted by the combination of the modelled cage and the pyridinium periphery as estimated from the QM/MM optimized geometry. Note that for electric field direction, the GAUSSIAN convention is used. The arrows at the right bottom represent the cartesian axis. All the electronic structure calculations were carried out for the doublet ground state of the complex.

To ascertain the role of the LEF on the initiation of the hydroxylation *via* an electron transfer from tetralin to HM1, we removed the cage, solvent and methyl substitutions of the HM1 from the optimized RS complex to mimic a zero-field environment. Thereafter, we performed a single-point calculation of the RS and its singly occupied SNOs for the reactant geometry (see ESI for Mulliken spin density and charges, Table S2†). Our calculations revealed that in the absence of the LEF, HM1 retains its electrons and so does the substrate TLN (see Fig. 9A). As such, ET does not occur in the absence of the LEF of the cage. We then applied an oriented external electric field (OEEF) of similar magnitude as the LEF exerted by the cage and substituted HM1 (the original RS). Consequently, now an electron is extracted from the substrate and regenerates an electronic structure which is in line with the one observed by using the modeled-cage scenario (see Fig. 9B). This provides compelling evidence that it is the LEF of the system that mediates the initial ET step, leading to the generation of RS, and subsequently, the reaction proceeds quickly.

#### 3.2.5 Does the LEF also regulate the *R*/*S* enantioselectivity?

To probe the origins of the reaction's enantioselectivity, we computed the LEF along the *y*-axis for RS, TS1(*R*), and TS1(*S*) (see Table 1). The LEF was found to be directed along positive *y*-axis for all the species. At the same time, the dipole moment for TS1(*R*) is aligned along the stabilizing direction vis-à-vis the LEF.

**Table 1:** Total dipole moment *μ* and their *x*, *y*-components. Local electric field (LEF) along *y*- and *z*-axis and their contributions for electrostatic stabilization for different states. Note that GAUSSIAN conversion is used to define direction of electric field and dipole moment; and iron–oxo, porphyrin, & substrate are used for dipole moment calculation

States	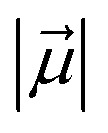 (D)	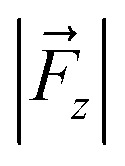 (V Å^−1^)	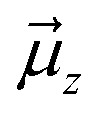 (D)	Δ*E*_*z*_ (kcal mol^−1^)	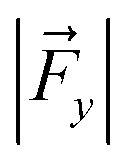 (V Å^−1^)	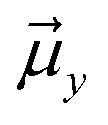 (D)	Δ*E*_*y*_ (kcal mol^−1^)
RS	8.87	−0.29	8.67	−12.07	0.19	1.76	1.61
TS1(*R*)	13.88	−0.27	13.85	−17.95	0.20	−0.40	−0.32
TS1(*S*)	10.82	−0.19	10.59	−9.66	0.23	1.66	1.84

Let us then proceed with the calculation of stabilization energy of the two chiral isomers of the TS in the presence of the 2D-LEF. For this purpose, we employ the following relationship:
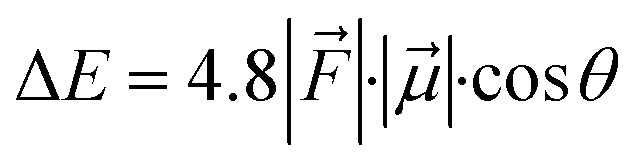
Here, Δ*E* is stabilization/destabilization energy in kcal mol^−1^, 
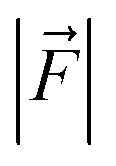
 is the LEF intensity in V Å^−1^, the 
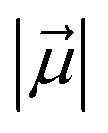
 is the magnitude of dipole moment in Debye and *θ* is the angle between LEF and dipole moment vectors. The results are collected in Table 1.

Table 1 reveals that the TS1(*R*) is better aligned than TS1(*S*) in the direction of the LEF, and it possesses relatively higher dipole moments along the *y*- and *z*-axes. As such, this dipole moment of TS1(*R*) will interact more strongly with the LEF than TS1(*S*), which possesses a lower *z*-LEF and higher *y*-EEF which is misoriented and destabilizes this TS. As such, the pro-*S* TS is less stabilized compared with the pro-*R* TS. The interaction energy calculations predict that the two directional electric fields complement each other for the reactivity and the selectivity. Clearly therefore, the LEF of the system is the decisive factor for the exclusive *R*-selective pathway and for its enhanced reactivity. This was further supported by a separate calculation, in which we stripped the cage and charged substitution, and found no enantioselectivity (see ESI, Fig. S13†).

### 3.3 Can the LEF of the supramolecular cage be tweaked to get enantioselectivity at will?

**Fig. 10:**
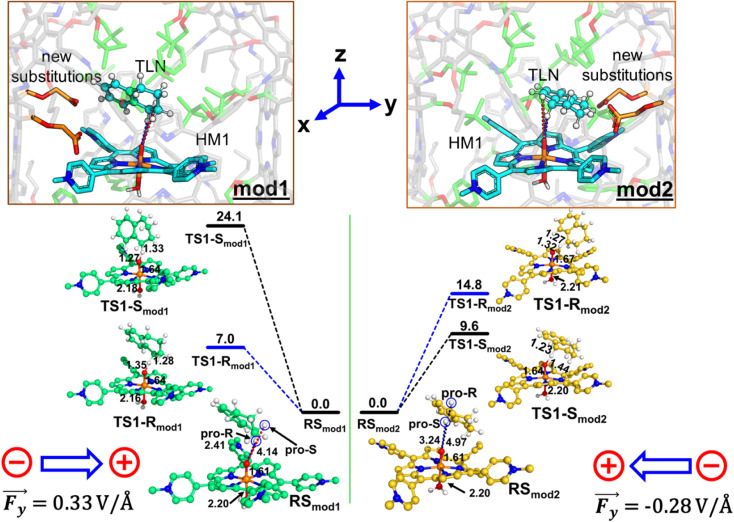
The ZPE corrected QM/MM/UB3LYP/def2-TZVP reaction profile diagram along with the optimized structures and key geometric data for mod1 and mod2 cages. PES scanning for HAT has been performed for the doublet ground state of the complex. Distances are in Å, and energies are reported in kcal mol^−1^ relative to RS. The gaussian convention is used for electric field vectors.

Since a *y*-directional electric field in our cage causes *R*-enantioselective reaction, we set out to reverse the LEF of the cage (see ESI, Fig. S14†) and produce *S*-enantioselectivity. When modifications were done along the *y*-axis for both the cases, this removed the pre-existing *y*-LEF in both directions. Fig. 10 shows that when we place a charged substituent in the negative *y*-direction (mod1), we achieve an absolute *R* enantioselectivity. The explicitly introduced groups are the carboxylate terminals which have −1 electronic charge for both the groups (total −2), which overpowers the overall LEF, leading to a dominant electrostatic effect along the *y*-axis in both the positive and negative directions. In contrast, when we place the same charged substitution on the positive *y*-direction (mod2), the reaction becomes *S*-enantioselective. Therefore, simply flipping the direction of the designed LEF in the supramolecular cages creates absolute enantioselectivity at will!

## 4. Conclusions

In the present work, we demonstrate that a methodological design of LEF in a supramolecular cage brings about the desired reactivity and selectivity of C–H hydroxylation reactions. Using rational designing principles, the supramolecular cage can be strategically modified to entrap the desired product. Furthermore, the designed local electric field lateral to the reaction axis provides absolute enantioselectivity of the product, and it can be tailored to produce the desired enantioselectivity. Finally, the LEF along the reaction axis can readily facilitate the electron transfer process, which in turn, enhances the reactivity several fold.

As such, the present work demonstrates a new state-of-art catalyst design which is sustainable and recyclable for different product formations.

## Data availability

All data needed to reproduce the work, are available in ESI material associated with this article.†

## Author contributions

SAS and SK perfomed the calculations and data analysis. KDD supervised and conceptualized the project. KDD and SS wrote the manuscript.

## Conflicts of interest

There is no conflict to declare.

## Supplementary Material

SC-014-D3SC01720F-s001

SC-014-D3SC01720F-s002
