# Supplementary material for: A porphyrin-based molecular cage guided by designed local-electric field is highly selective and efficient
Source: Chem Sci. 2023 Sep 4;14(37):10329–39. doi: 10.1039/d3sc01720f (PMC10529934; doi:10.1039/d3sc01720f)
Supplement: SC-014-D3SC01720F-s001 [file SC-014-D3SC01720F-s001.pdf]

Electronic Supplementary Material (ESI) for Chemical Science.
This journal is © The Royal Society of Chemistry 2023

Supporting Information

A Porphyrin-Based Molecular Cage Guided by Designed Local-Electric Field Is Highly Selective and Efficient

Shakir Ali Siddiqui[1], Sason Shaik[2]*, Surajit Kalita[1] and Kshatresh Dutta Dubey[1]*

1. Department of Chemistry, School of Natural Sciences, Shiv Nadar Institution of Eminence Delhi-NCR.
2. Institute of Chemistry, The Hebrew University of Jerusalem Israel, Jerusalem, Israel.

* sason@yfaat.ch.huji.ac.il, kshatresh.dubey@snu.edu.in,

Table of Contents:

Pages

Figure S1. Primary altered cage for HM1 entrapment……………………..………………S3

Figure S2. Modifications for *modeled cage*………..……………..………………………S3

Text S1……..……………………………………………………..…………………S4

Text S2……………………………………………………………..…………..……S5

Text S3……………………………………………………..…..………………S6

Text S4 with **Figure S3 & S4** ………………………………………..……………..S6-S8

Figure S5. Tetralin (TLN) docked in the active site………..…..…………………………S9

Figure S6. RMSD during 100 ns MD simulation……………..…………..………………S9

Figure S7. A representative MD snapshot highlighting the substitutions for substrate entrapment…………………………………………………..……..……………………S9

Figure S8. Evolution of pro-R(H) and pro-S(H) distances for replica simulation………...S10

Figure S9. QM/MM TS1(R) and TS2(R) optimized structures…………….……….…..S10

Figure S10. QM/MM optimized structures for (R)-PES scan in sextet spin state…………...…S10

Figure S11. QM/MM (S)-PES scan profile & Comparative rate constant data……………S11

Figure S12. QM/MM optimized structures for (S)-PES scan in sextet spin state……..…....S12

Figure S13. Single-point gas-phase profile for RS, TS1(S) and TS1(R) doppelgangers, in absence and presence of OEEF……….…………………………….………..…………...S12

Table S1. Mulliken spin and charges for both the R- and S- reaction profiles………………S13

Table S2. Mulliken spin and charges for RS doppelganger species…..………..…………...S13

Figure S14. Modifications for generating mod1 and mod2……,,,…………….…………...……S14

Figure S15. Distance analysis for pi-pi interactions….……………………..………………S14

Table S3. QM/MM energy values for all the systems ……………………...……………S15

References……………………………………..……….……..…………………...S16

QM region for QM/MM optimized geometries.……..………S17-S62

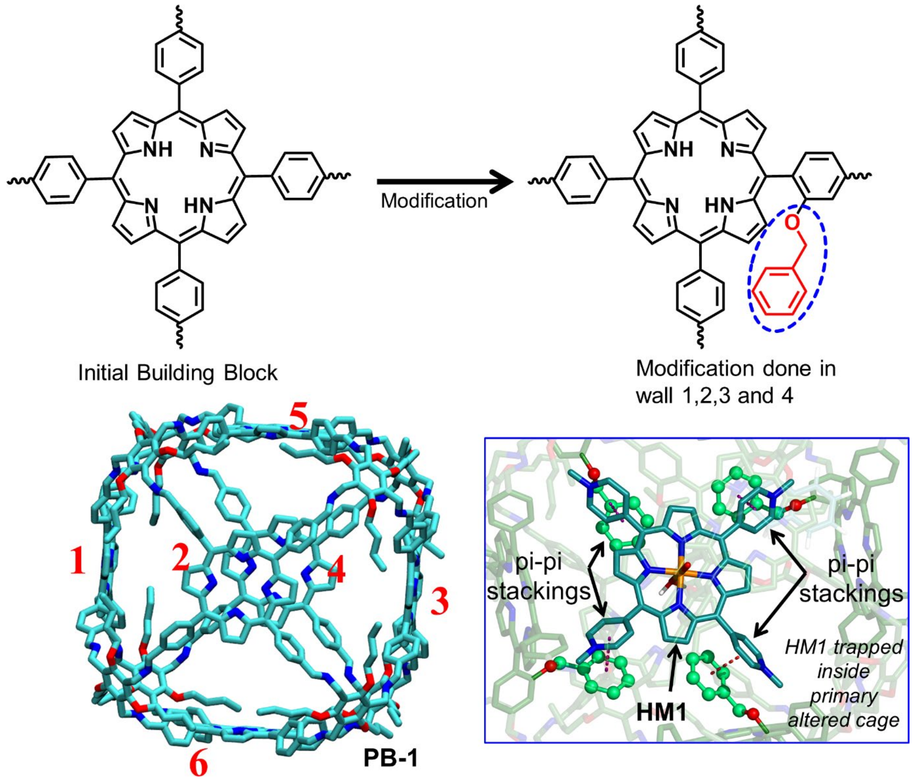

Figure S1. Modification done for primary altered cage for HM1 entrapment through pi-pi stacking interactions.

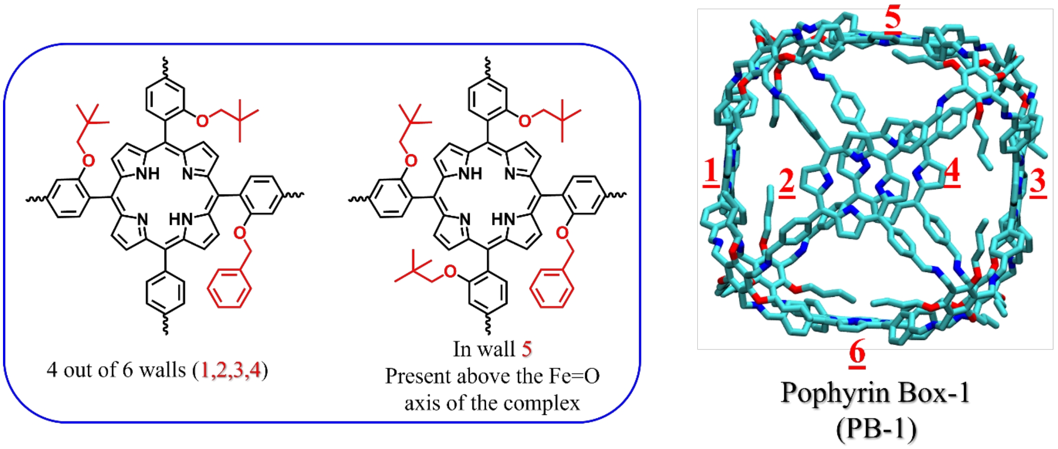

Figure S2. Modifications made in the porphyrin box-1 (PB-1) skeleton for computationally designing the *'modeled cage'* with a local electric field (LEF) aligned along the Fe=O axis of active oxidant HM1. The right-hand side figure shows the structure of PB-1 synthesized by K. Kim et al.[2], H's are omitted for clarity. Numbers 1, 2, 3, 4, 5, and 6 represent the six faces of the box. The left-hand side figure shows the modifications made in red to the faces/walls of the PB-1 skeleton.

Text S1. The structural modification/functionalization for the phenyl, alkyl or other substitutions in the PB-1 can be done through pre-synthetic approach as well as some post synthetic modifications which has been extensively discussed by Yusran et al.[1] For both the approaches, the scaffold should be quite stable towards the modifications and as discussed by K. Kim et al., the PB-1 possesses the applicability and exceptional chemical stability[2] and it has been already subjected to various modifications.[3,4] We have utilized the concept of covalent linkage modification which can be done using functionalizing the already existing pendant groups in the skeleton via triazole linkages, ester linkages, ether linkages, amide linkages etc.[1] These pendant groups can be added with the pre-synthetic approaches in the building block. Herein, we have applied the ether covalent linkage as a source of inspiration for the post synthetic modification that can be generated by the coupling of the hydroxyl (–OH) pendant group (which can be pre-functionalized in the building block meso-porphyrins) and the alkyl or aryl-halide (Scheme S1). Subsequently, the desired porous organic cages were modeled computationally.

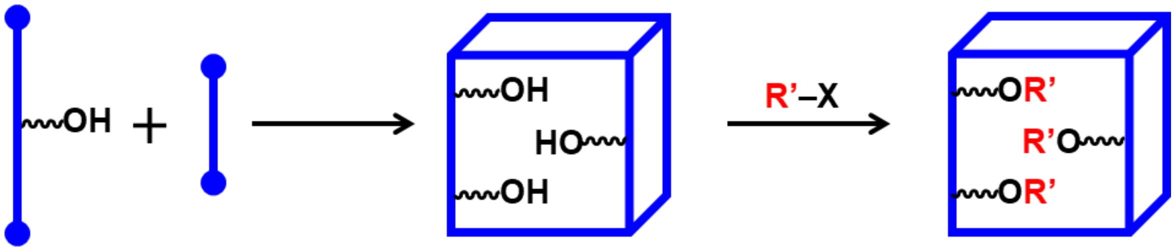

Scheme S1. Structural modification strategy of the cage using post synthetic modifications.

By encasing the guest in the host in the manner of a "ship-in-a-bottle," porphyrin or metalloporphyrin derivatives can be effectively encapsulated inside the cage. One of the most often used strategies to capture a moiety with dimensions larger than the host's aperture size is the ship-in-a-bottle technique.[5]

Text S2. Spin-state Selectivity Calculation:

To calculate the percentage of each spin state (doublet and sextet) for Fe, we can use the Boltzmann population formula:

$P(D) / P(S) = \exp[-((\Delta G(D) - \Delta G(S)) / kT)]$

where P(D) and P(S) are the populations of the doublet and sextet states, ΔG(D) and ΔG(S) are the energy differences between the doublet and sextet states, k is the Boltzmann constant ($8.617333262145 \times 10^{-5}$ eV/K), and T is the temperature in K.

The calculated transition state energy barriers: ΔG(D) = 4.8 kcal/mol and ΔG(S) = 7.3 kcal/mol, it is needed to convert to the corresponding energy units (eV) for consistent calculations. Assuming the temperature of 298 K:

$\Delta G(D) = 4.8$ kcal/mol × 4.184 eV/kcal = 20.07 eV

$\Delta G(S) = 7.3$ kcal/mol × 4.184 eV/kcal = 30.49 eV

Now, we can calculate the population ratio:

$$P(D) / P(S) = \exp[-(\Delta G(D) - \Delta G(S)) / kT]$$

$$= \exp[-(20.07 - 30.49) / (8.617333262145 \times 10^{-5} \times 298)]$$

$P(D) / P(S) \approx \exp(114.78)$

Taking the natural logarithm of both sides:

$\ln(P(D) / P(S)) \approx 114.78$

Taking the exponential (antilog) of both sides:

$P(D) / P(S) \approx e^{114.78} \approx 3.22 \times 10^{49}$

Therefore, the population ratio of the doublet state to the sextet state is estimated to be approximately 3.22×10^{49}.

To estimate the spin selectivity, we can use the following formula:

Spin Selectivity = $(P(D) / (P(D) + P(S))) \times 100$

Plugging in the population ratio:

Spin Selectivity $\approx (3.22 \times 10^{49} / (3.22 \times 10^{49} + 1)) \times 100 \approx 99.99\%$

Based on these calculations, the estimated spin selectivity is >99.9% for the doublet state.

Text S3. Consistency in local electric field calculations in TITAN and TUPA codes:

The TITAN-code and TUPÃ both are used to calculate the magnitude and direction of electric fields (EFs) imposed by surrounding charged groups (point charges). These calculations are mainly based on Coulomb's law for both the codes. Coulomb's law describes the electrostatic interaction between charged particles, and it is a fundamental principle used to calculate electric fields. The specification of the point charges is exactly the same in both cases. Therefore, it is reasonable to expect that the calculation of electric field between both the two codes should be consistent with each other.

However, factors such as numerical approximations and computational precision can still introduce some discrepancies. Therefore, we selected 5 most representative MD snapshots from the clustering analysis and subjected it to LEF calculations by both TITAN and TUPA code. The LEF values from both the codes were exactly matching to one another. Therefore, it is evident that the electric field results are consistent between the two codes. The LEF values are summarized in the adjoining table:

MD Snapshot	LEF along Fe–O axis (in a.u.)		LEF along Fe–O axis (in V/Å)	
	TITAN	TUPA	TITAN	TUPA
**1**	0.0056877579	0.0056640796	0.29235075606	0.2911336948
**2**	0.0056483091	0.0058617523	0.29032308774	0.3012940692
**3**	0.0053655644	0.0054019565	0.27579001016	0.2776605670
**4**	0.0052873262	0.0051657775	0.27176856668	0.2655209680
**5**	0.0050722410	0.0049986949	0.26071318740	0.2569329225

Text S4. Entry/Exit of the Substrate/Product

To investigate the process of substrate/product binding and unbinding, we employed adaptive steered molecular dynamics (ASMD) simulation and calculated the potential of mean

force (PMF) using the Jarzynski average.[6] Previous studies has shown that the PMF values qualitatively fit with the theoretical and experimental binding free energy calculations.[7] The results of this calculation are illustrated in Figure 11a, with PMF values of ~15.5 and ~15.9 kcal/mol for the substrate (tetralin) and the hydroxylated product (*R*-1-tetralol), respectively. The smaller values of free energies as observed by the PMF plot show a feasible substrate entry and exit from the supramolecular cage.

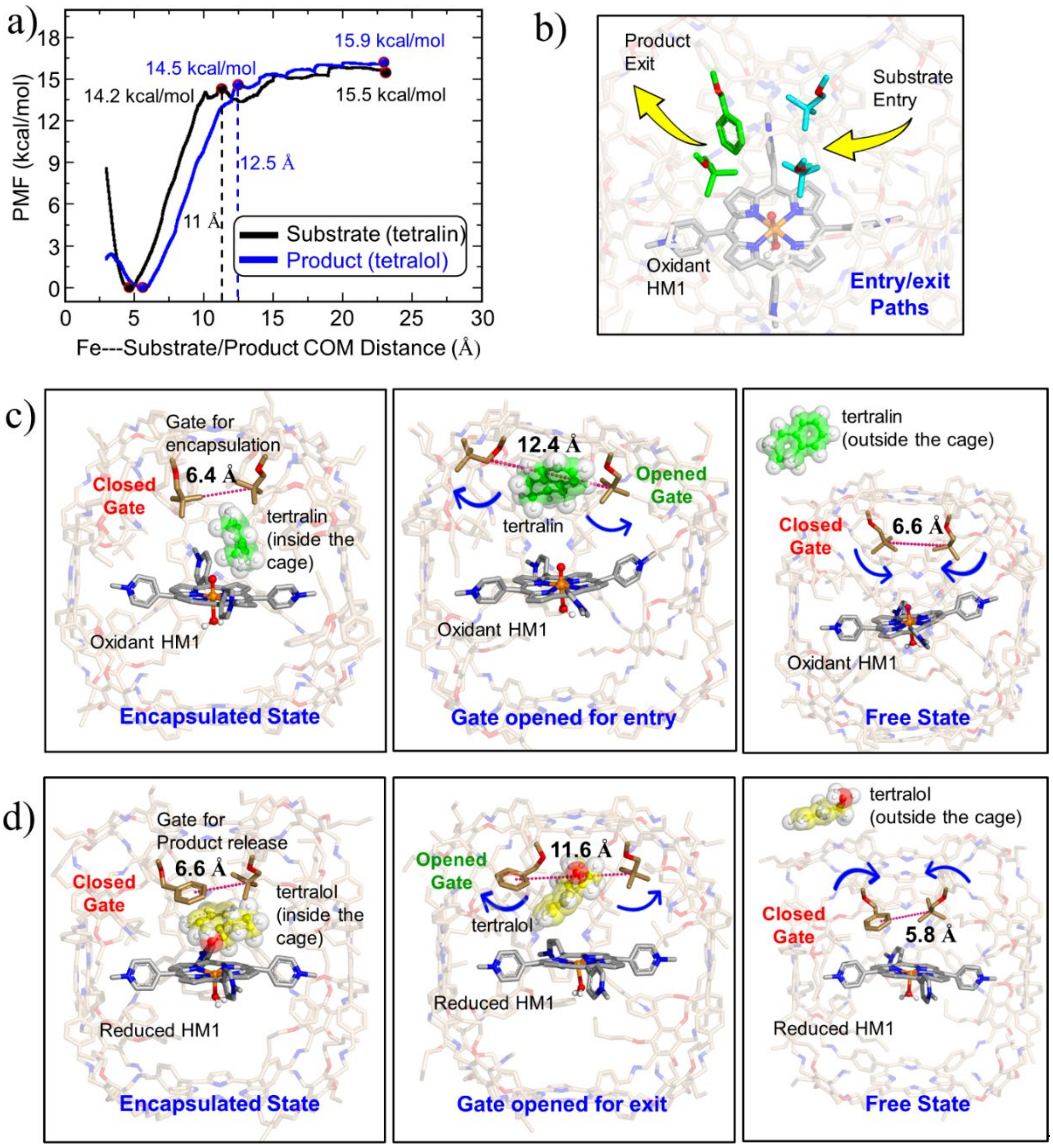

Figure S3. (a) ASMD derived PMF along the reaction coordinate for both, substrate & hydroxylated product. (b) Obtained paths for substrate entry and product exit from the cage. (c,d) Representative states for unbinding/binding process for substrate and product, respectively. The distances for the movement of crucial groups are in Figure S4.

During the exit, we observe a sharp increase in the PMF, i.e., free energy for both substrate and product as they dissociate from the active site. The rise in PMF is mainly due to the force against the hydrophobic interactions (Figure S7) between substrate and cage-substituents when substrate/product moves from 3 to 10-12Å from the reactive center, i.e., Fe=O for **HM1**. Interestingly, we observed that both t-butyl groups and the phenyl substitution of the cage act *as doorways which can easily be shuttles from open to close conformation to allow the substrate entry and the product exit* (Figure S3b-d). Interestingly, once substrate enters or product exists the cage, the two t-butyl groups and the phenyl groups, restore their previous orientations, indicating an important dynamics during the entry/exit of the substrate/product. As can be seen, the PMF becomes linear after the substrate/product dissociates the cage at 23 Å indicating no further force is applied. *The total work (PMF) implies that the substrate can easily access the active oxidant inside the modeled cage, while the hydroxylated product can escape and leave the space*. These findings shed light on the energetics and dynamics of the substrate/product dissociation process and have important implications for the design and optimization of supramolecular cages for catalytic applications.

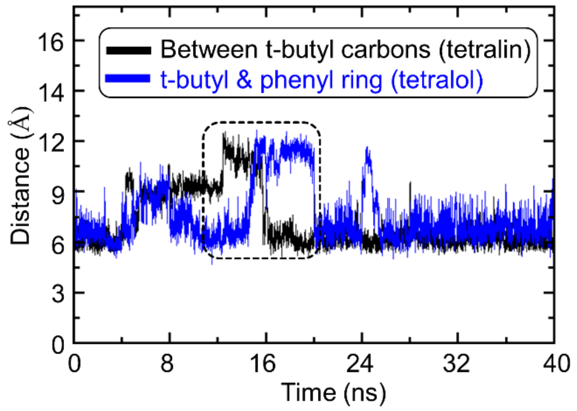

Figure S4. The mutual distance for the gatekeeper groups observed during ASMD simulations.

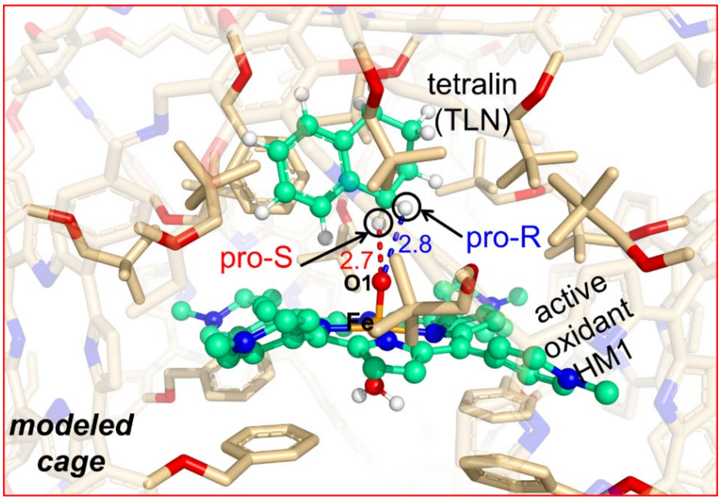

Figure S5. The substrate tetralin (TLN) docked into the so-created active site speculating the initial distances of pro-R(H) and pro-S(H) from reactive O1 atom of active oxidant HM1.

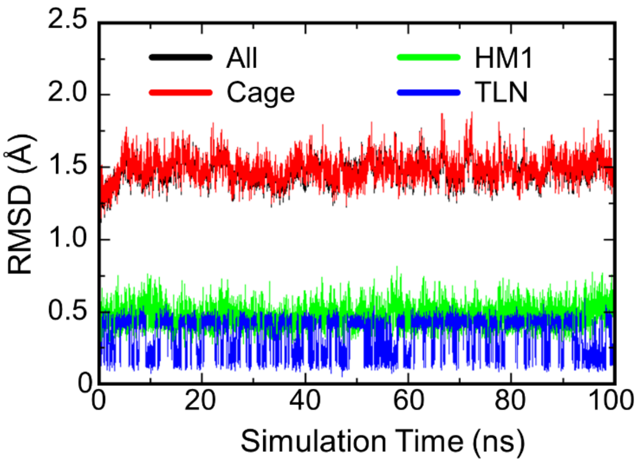

Figure S6. Root mean square deviation (RMSD) during 100 ns MD simulation for modeled cage.

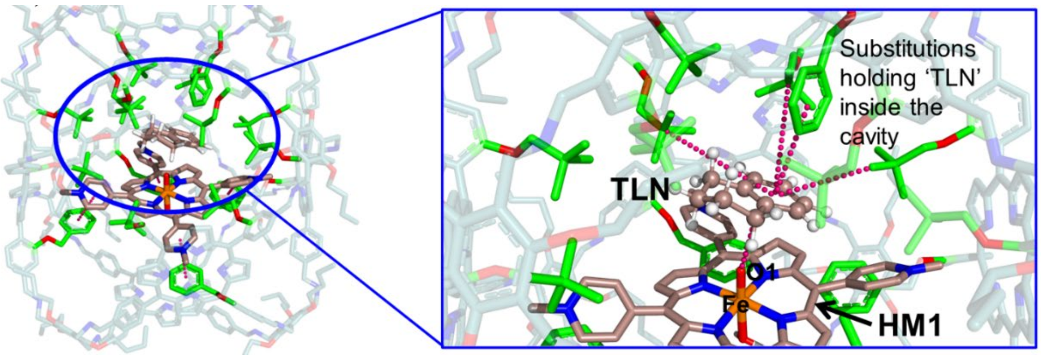

Figure S7. A representative MD snapshot highlighting the substitutions for substrate entrapment.

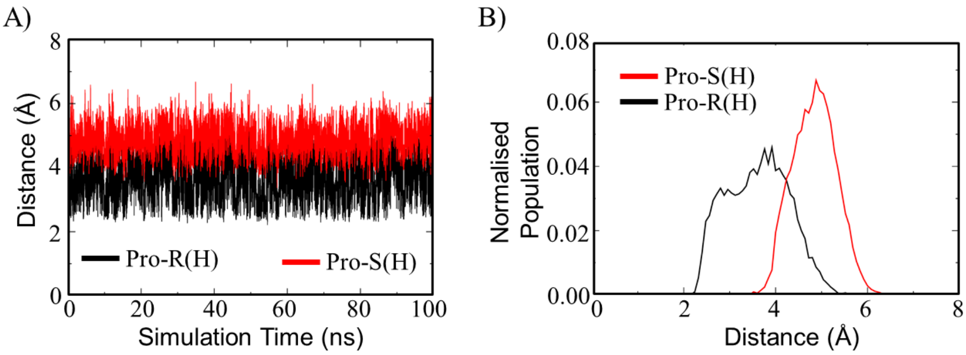

Figure S8. (A) Evolution of pro-R(H) and pro-S(H) distances from O1 atom of the active oxidant HM1 for 100 ns replica simulation. (B) The Boltzmann population distribution plot corresponds to distance plots between the pro-*R* and pro-*S* hydrogens with the reactive oxygen (N1) over the100 ns simulation.

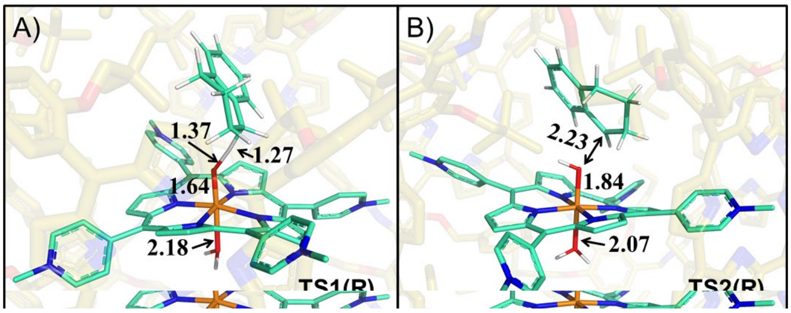

Figure S9. QM/MM optimized transition state structures for R-selective hydroxylation with critical geometric data in doublet spin state. TS1(R) and TS2(R) represent the transition state for H-abstraction and cation transfer steps, respectively. Bond lengths are in Å unit.

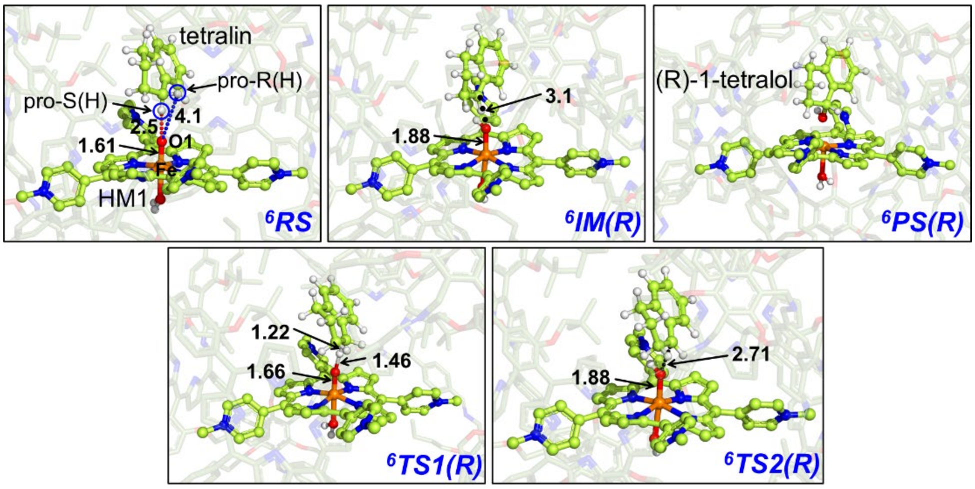

Figure S10. QM/MM optimized structures with critical geometric data for R-selective hydroxylation in sextet spin state, obtained during PES scan. ^{6}TS1(R) and ^{6}TS2(R) represent the transition states for H-abstraction and cation transfer steps, respectively. Bond lengths are in Å unit.

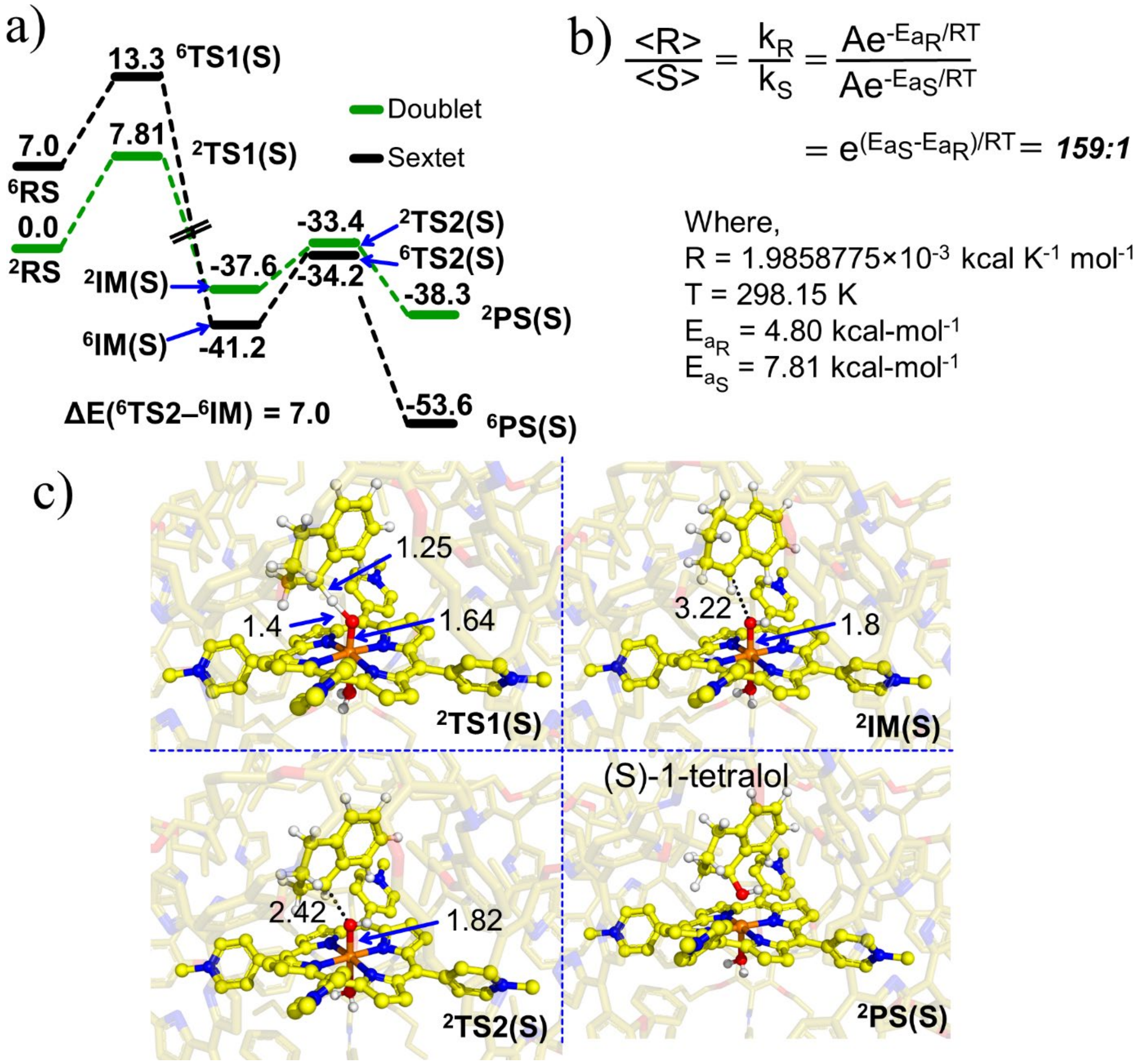

Figure S11. **(A)** ZPE corrected QM/MM/UB3LYP/def2-TZVP reaction profile diagram for the S-enantioselective C-H hydroxylation of tetralin into S-(1)-tetralol by active oxidant HM1 entrapped inside the modeled cage. **(B)** Comparative rate constant data for R- and S-selective hydroxylation calculated using the Arrhenius equation. **(C)** QM/MM optimized structures for S-selective hydroxylation with critical geometric data.

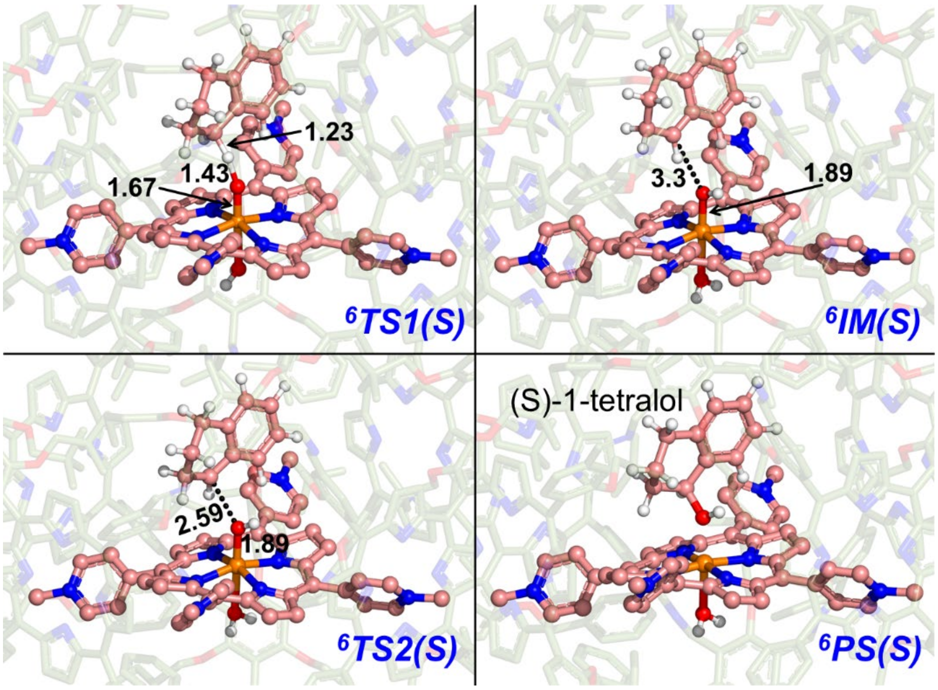

Figure S12. QM/MM optimized structures with critical geometric data for S-selective hydroxylation in sextet spin state, obtained during PES scan. ^{6}TS1(S) and ^{6}TS2(S) represent the transition states for H-abstraction and cation transfer steps, respectively. Bond lengths are in Å unit.

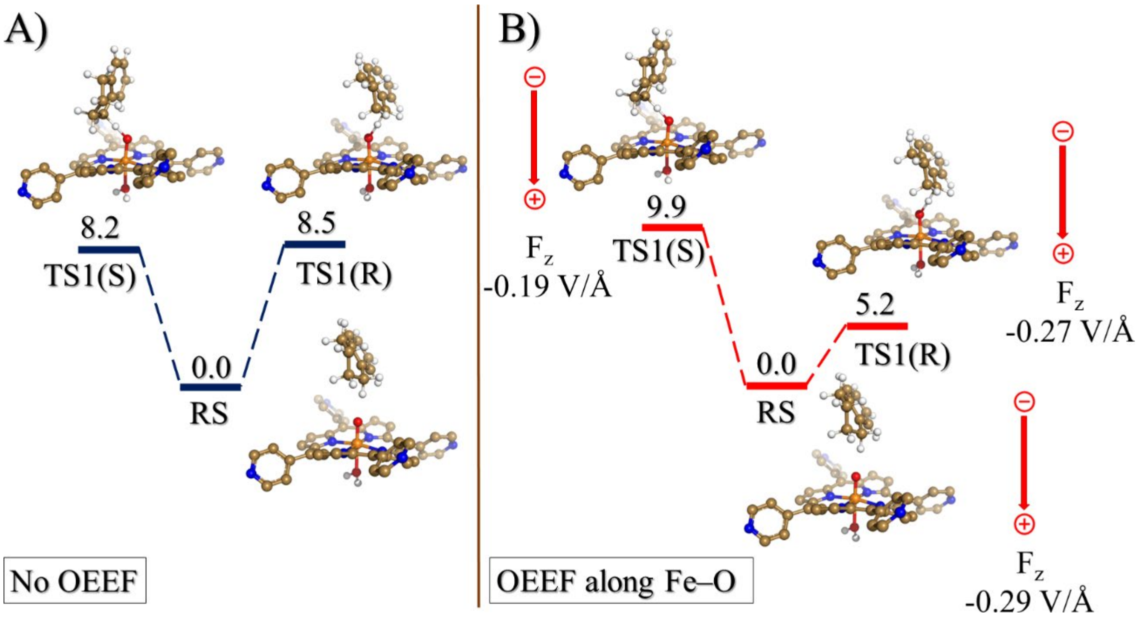

Figure S13. The zero-point corrected UB3LYP/def2-TZVP single-point gas-phase energy profile diagram for RS, TS1(S) and TS1(R) doppelgangers, in (A) the absence of EF and (B) presence of OEEF along Fe–O axis (negative z axis). For electric field vectors, gaussian convention is used.

Table S1. Mulliken spin and charges of RS, IM, and PS species for both the R- and S- reaction profiles (modeled cage).

**Mulliken Spin**										
	**^{2}RS**	**^{2}IM(R)**	**^{2}PS(R)**	**^{2}IM(S)**	**^{2}PS(S)**	**^{6}RS**	**^{6}IM(R)**	**^{6}PS(R)**	**^{6}IM(S)**	**^{6}PS(S)**
**Fe-O1**	2.07	1.07	1.06	1.07	1.05	3.74	4.54	4.29	4.54	4.36
**Rest of Complex**	-0.13	-0.07	-0.06	-0.07	-0.05	0.40	0.34	0.56	0.36	0.56
**Substrate**	-0.86	0.00	0.00	0.00	0.00	0.81	0.00	0.00	0.00	0.00
**Mulliken Charge**										
	**RS**	**IM(R)**	**PS(R)**	**IM(S)**	**PS(S)**	**^{6}RS**	**^{6}IM(R)**	**^{6}PS(R)**	**^{6}IM(S)**	**^{6}PS(S)**
**Fe–O1**	0.32	0.24	0.33	0.24	0.32	0.47	0.46	0.59	0.47	0.57
**Rest of Complex**	3.85	3.86	4.00	3.86	4.00	3.86	3.46	4.00	3.47	4.00
**Substrate**	0.83	0.90	0.34	0.90	0.34	0.80	1.07	0.27	1.05	0.27

Table S2. Mulliken spin and charges of N-methyl stripped RS doppelganger species at the oriented external electric field (OEEF) = 0 and -0.29 V/Å along the Fe=O1 axis.

	**Mulliken Spin**		**Mulliken Charge**	
	**0.0 V/Å**	**-0.29 V/Å**	**0.0 V/Å**	**-0.29 V/Å**
**Fe–O1**	2.06	2.06	0.32	0.31
**Rest of Complex**	-1.06	-0.07	0.70	-0.28
**Substrate**	0.00	-1.00	-0.02	0.97

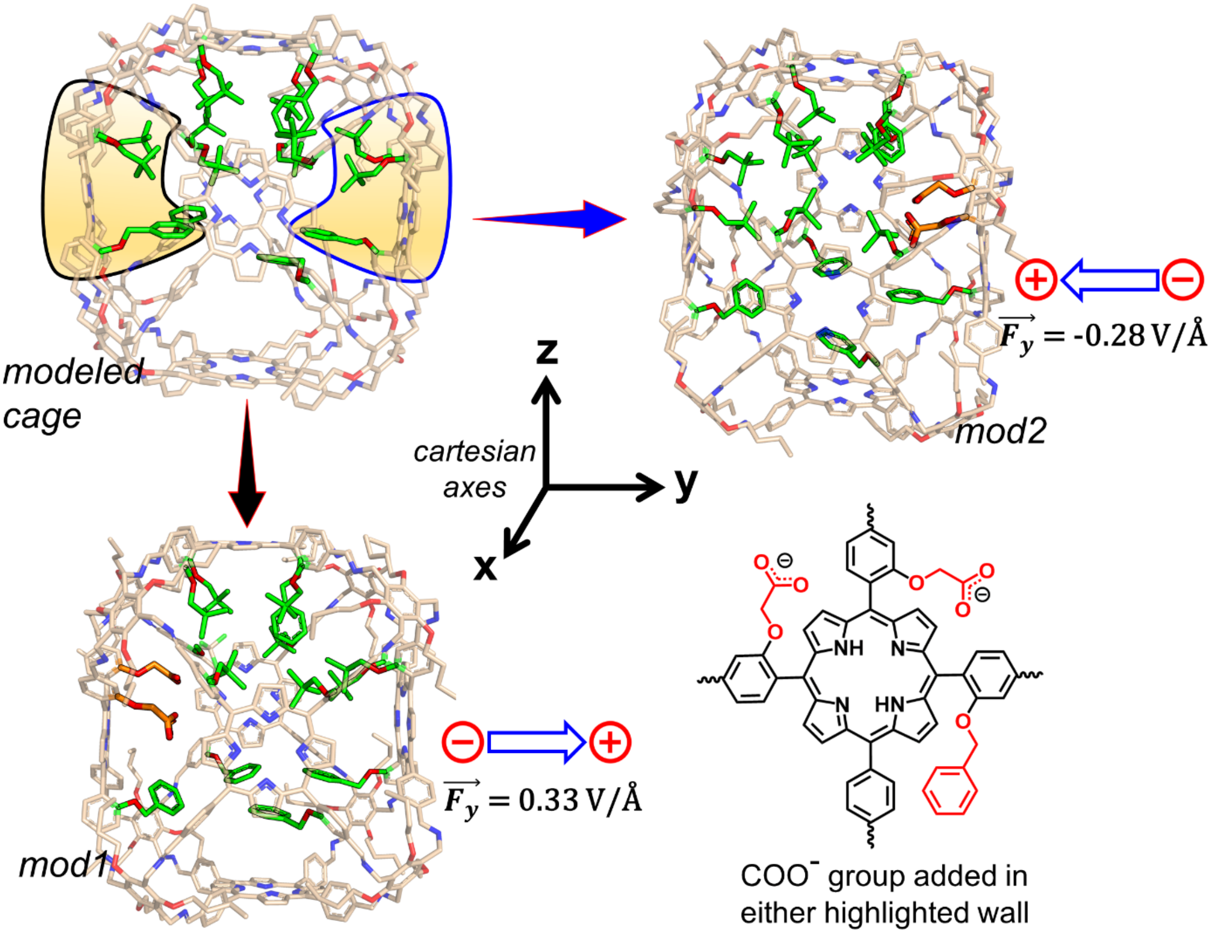

Figure S14. Modification done in either highlighted wall of modeled cage for generating mod1 and mod2. Local electric field (LEF) along y-axis due to modification is also shown.

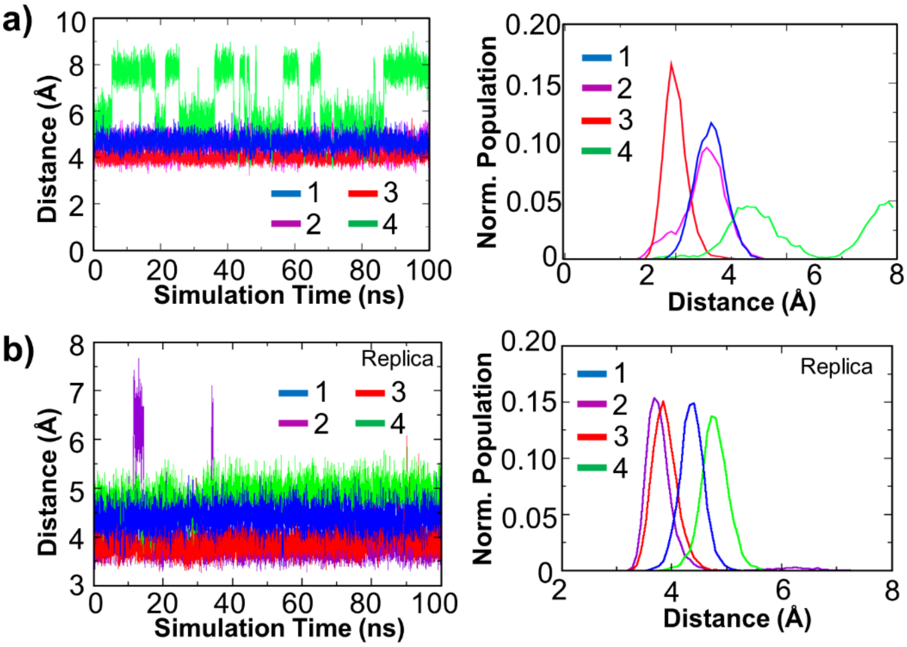

Figure S15. Characterization of pi-pi interactions present between HM1 and cage to stabilize the HM1 inside the cage. The mutual distance between rings of the HM1 and the phenyl rings of the modeled supramolecular cage, (a) for MD simulations, (b) for another replica simulation.

Table S3. QM/MM energy values for all the systems. Energy units are in a.u. B1, B2 and D3 stand for def2-SVP, def2-TZVP and Grimme's Dispersion respectively.

	**B1**	**B2**	**B2+D3**	**ZPE**
**^{2}RS**	-3936.253600	-3939.2999	-3939.4429	0.963369031
**^{2}TS1(R)**	-3936.243468	-3939.2868	-3939.4374	0.957975041
**^{2}IM(R)**	-3936.3388	-3939.3788	-3939.524	0.972140886
**^{2}TS2(R)**	-3936.3291	-3939.3706	-3939.5265	0.96922187
**^{2}PS(R)**	-3936.3457	-3939.3805	-3939.5398	0.970948112
**^{2}TS1(S)**	-3936.2472	-3939.2897	-3939.4427	0.957905446
**^{2}IM(S)**	-3936.3321	-3939.3763	-3939.5215	0.972140924
**^{2}TS2(S)**	-3936.3165	-3939.3673	-3939.5127	0.970201639
**^{2}PS(S)**	-3936.3370	-3939.3820	-3939.5344	0.976732388
**^{6}RS**	-3936.2423	-3939.2882	-3939.4342	0.96293547
**^{6}TS1(R)**	-3936.2376	-3939.2833	-3939.433	0.95858311
**^{6}IM(R)**	-3936.3286	-3939.3759	-3939.5224	0.97358339
**^{6}TS2(R)**	-3936.3245	-3939.3734	-3939.5215	0.9727141
**^{6}PS(R)**	-3936.3576	-3939.3959	-3939.5537	0.97449413
**^{6}TS1(S)**	-3936.2274	-3939.2714	-3939.4231	0.95616875
**^{6}IM(S)**	-3936.3281	-3939.3744	-3939.5209	0.97227416
**^{6}TS2(S)**	-3936.3164	-3939.3629	-3939.5141	0.97182621
**^{6}PS(S)**	-3936.3556	-3939.3938	-3939.5533	0.97189892
**^{2}RS$_{mod1}$**	-3936.8143	-3939.863	-3939.0438	0.96484176
**^{2}TS1-R$_{mod1}$**	-3936.7995	-3939.8452	-3939.998099	0.95821706
**^{2}TS1-S$_{mod1}$**	-3936.7738	-3939.8182	-3939.969374	0.95855375
**^{2}RS$_{mod2}$**	-3936.8369	-3939.8853	-3940.038961	0.96675416
**^{2}TS1-R$_{mod2}$**	-3936.8111	-3939.856	-3940.020040	0.96106259
**^{2}TS1-S$_{mod2}$**	-3936.8191	-3939.8643	-3940.028253	0.96104858

References:

1 Y. Yusran, Q. Fang and S. Qiu, *Isr. J. Chem.*, 2018, **58**, 971–984.

2 S. Hong, M. R. Rohman, J. Jia, Y. Kim, D. Moon, Y. Kim, Y. H. Ko, E. Lee and K. Kim, *Angew. Chemie - Int. Ed.*, 2015, **54**, 13241–13244.

3 P. T. Smith, B. P. Benke, L. An, Y. Kim, K. Kim and C. J. Chang, *ChemElectroChem*, 2021, **8**, 1653–1657.

4 L. An, P. De La Torre, P. T. Smith, M. R. Narouz and C. J. Chang, *Angew. Chemie Int. Ed.*, 2023, **62**, e202209396.

5 J. Chakraborty, I. Nath and F. Verpoort, *Coord. Chem. Rev.*, 2016, **326**, 135–163.

6 C. Jarzynski, *Phys. Rev. Lett.*, 1997, **78**, 2690.

7 N. Shi, Q. Zheng and H. Zhang, *ACS Chem. Neurosci.*, 2022, **13**, 1526–1533.

QM region for QM/MM optimized geometries:

For *modeled cage:*

^{2}RS

N 26.9476408 29.9956137 23.9125132
N 25.8465862 27.4397354 24.3478479
N 28.1412405 26.7405672 25.8981098
N 29.3131899 29.2393443 25.2962543
C 25.8176145 30.1294860 23.1557746
C 27.5032922 31.2535479 24.0143636
C 27.3233466 25.6997550 26.2877451
C 29.3552120 26.5330956 26.5016708
C 25.0240264 27.7906525 23.2980338
C 25.4568606 26.1907186 24.7427133
C 29.6722712 30.5118883 24.9393723
C 30.4632940 28.6062402 25.7108993
C 25.5886184 31.5272152 22.8593416
C 26.6146580 32.2258460 23.4178951
C 28.0198547 24.8612940 27.2334494
C 29.2789566 25.3628631 27.3552596
C 24.1506533 26.6831567 22.9708405
C 24.3889389 25.7111351 23.8920548
C 31.1033263 30.6710839 25.0623469
C 31.5980416 29.4838185 25.5062301
C 24.9748021 29.0726458 22.7277396
C 28.8045233 31.5225153 24.4680674
C 30.5076846 27.3447582 26.3391839
C 26.0470705 25.4216376 25.7716118
H 24.7275804 31.9307808 22.3352022
H 26.7442343 33.3039744 23.4285401
H 27.5953679 24.0029061 27.7432903
H 30.0662015 24.9844678 28.0007346
H 23.4347942 26.6360093 22.1566833
H 23.9149059 24.7351028 23.9501802
H 31.6682363 31.5622222 24.8034019
H 32.6425290 29.2420779 25.6722453
C 24.0145411 29.3612048 21.6253857
C 29.3677174 32.9073634 24.3280394
C 31.8088799 26.8462491 26.8774889
C 25.2587294 24.2425906 26.2566985
C 24.4716504 29.9913522 20.4479573
C 23.5923786 30.2883895 19.4244233
N 22.2818352 29.9641687 19.5093091
C 22.6435107 29.0426525 21.6794215
C 21.8065474 29.3397848 20.6117629
H 25.5217566 30.2565896 20.3217702
H 23.9133132 30.7933334 18.5145342
H 22.1980355 28.5899421 22.5628845
H 20.7421571 29.0951165 20.6226952
C 23.9573143 24.4474575 26.7606929
C 23.1622960 23.3795886 27.1357301
N 23.6134770 22.1089406 27.0275384
C 25.7066507 22.9094500 26.2009460
C 24.8696905 21.8689858 26.5910818
H 23.5482057 25.4504713 26.8637946
H 22.1477597 23.5088723 27.5199204
H 26.6963037 22.6564933 25.8187470
H 25.1804123 20.8229617 26.5474896
C 32.6785892 27.6468452 27.6471791
C 33.8858720 27.1410436 28.1096454
N 34.2454967 25.8603839 27.8641232

C	32.2348710	25.5232887	26.6333218
C	33.4344905	25.0573420	27.1401316
H	32.4211771	28.6679855	27.9213963
H	34.5860058	27.7455518	28.6890935
H	31.6284986	24.8384219	26.0459079
H	33.7756984	24.0333796	26.9779936
C	29.4990358	33.5304284	23.0723467
C	30.0989453	34.7786727	22.9587190
N	30.5751147	35.4265631	24.0455112
C	29.8356928	33.6346807	25.4433540
C	30.4290899	34.8791952	25.2773086
H	29.1586955	33.0379853	22.1604006
H	30.2239718	35.2816244	21.9972610
H	29.7339849	33.2411091	26.4562970
H	30.8029506	35.4643834	26.1216443
O	28.2874250	27.7393090	23.3806880
O	26.7600615	29.1087730	26.5643731
H	25.8252122	29.3654175	26.5677572
C	31.3121109	36.7081736	23.8792664
H	31.0438204	37.1575970	22.9154598
H	31.0390748	37.3962842	24.6881781
H	32.3918730	36.5016212	23.9114139
C	21.3776963	30.2784908	18.3798885
H	20.3741354	30.4728735	18.7753267
H	21.7486397	31.1696315	17.8594144
H	21.3487469	29.4225510	17.6892096
C	22.6887329	20.9975965	27.3543253
H	21.9193873	20.9357069	26.5734672
H	23.2537178	20.0615071	27.4074056
H	22.2154516	21.1960155	28.3230308
C	35.5376155	25.3425839	28.3686209
H	35.4651061	24.2561901	28.4923731
H	36.3349341	25.5961806	27.6543077
H	35.7495023	25.8060605	29.3392438
H	26.9221909	28.6558336	27.4046511
Fe	27.6271235	28.3027446	24.7380043
C	30.2450193	26.4538725	20.1136485
C	30.7764720	25.2977155	19.3953400
C	32.1428696	24.9766190	19.5241410
C	32.9870015	25.7910858	20.2539383
C	32.4777635	26.9639269	20.9046102
C	31.1286561	27.2730011	20.8396948
H	32.5318321	24.1032291	18.9972774
H	34.0507642	25.5599566	20.3289150
H	33.1678305	27.6055543	21.4587274
H	30.7314224	28.1409737	21.3705391
C	29.8855317	24.4557589	18.5422585
H	29.7810603	23.4820459	19.0647325
H	30.4218936	24.1833740	17.6163345
C	28.7844670	26.7778078	20.0541593
H	28.6886112	27.6576657	19.3797021
H	28.4622109	27.1407676	21.0470000
C	28.5014792	25.0418063	18.2595222
H	27.8501639	24.2584183	17.8485883
H	28.5845997	25.8207918	17.4823879
C	27.9002666	25.6423213	19.5285068
H	26.8850687	26.0233019	19.3390427
H	27.7998034	24.8592974	20.2992831

^{2}TS1(R)

N	27.1254323	29.8833185	23.6124582
N	25.9710955	27.3933735	24.1768278

N	28.2276509	26.7408966	25.8024296
N	29.4373180	29.1708829	25.0914636
C	25.9536022	30.0278476	22.9115636
C	27.6549752	31.1525646	23.7513135
C	27.3823325	25.7454951	26.2426135
C	29.4340330	26.5467810	26.4346864
C	25.1429937	27.7132307	23.1181461
C	25.5600991	26.1628882	24.6294774
C	29.8118921	30.4330715	24.6965657
C	30.5843955	28.5337815	25.5197794
C	25.6933397	31.4272863	22.6761985
C	26.7295905	32.1235962	23.2232847
C	28.0407311	24.9658233	27.2598503
C	29.3107519	25.4462200	27.3687565
C	24.2529135	26.6112465	22.8447121
C	24.4839264	25.6712684	23.8029011
C	31.2410017	30.5740993	24.8103061
C	31.7240803	29.3879731	25.2768748
C	25.0891540	28.9814929	22.5169558
C	28.9513077	31.4390383	24.2122138
C	30.6090357	27.3089160	26.2209901
C	26.1171209	25.4457758	25.7064888
H	24.8035597	31.8330483	22.2040485
H	26.8373548	33.2029254	23.2762688
H	27.5857968	24.1567388	27.8218738
H	30.0778132	25.0972200	28.0542042
H	23.5307500	26.5461049	22.0372541
H	23.9923962	24.7072933	23.9026301
H	31.8139063	31.4526722	24.5269525
H	32.7666909	29.1349088	25.4352112
C	24.0827953	29.2803599	21.4584577
C	29.4943462	32.8328606	24.0996870
C	31.8997332	26.8119315	26.7924843
C	25.3089428	24.2970263	26.2331344
C	24.4916133	29.9235103	20.2704980
C	23.5672378	30.2546112	19.2985400
N	22.2567949	29.9564594	19.4466511
C	22.7130944	28.9751066	21.5716178
C	21.8280858	29.3108459	20.5548827
H	25.5370683	30.1799994	20.0964634
H	23.8508819	30.7693492	18.3824805
H	22.3016437	28.5099005	22.4645115
H	20.7620813	29.0830035	20.6152615
C	23.9978822	24.5307657	26.6994038
C	23.1925013	23.4814137	27.1065854
N	23.6421334	22.2057237	27.0655030
C	25.7545030	22.9627561	26.2472059
C	24.9058803	21.9425607	26.6665239
H	23.5879657	25.5394753	26.7425997
H	22.1702391	23.6288460	27.4636264
H	26.7509370	22.6912110	25.8962598
H	25.2138945	20.8947701	26.6762074
C	32.8118856	27.6413499	27.4790426
C	33.9988015	27.1316451	27.9870725
N	34.3050000	25.8210087	27.8568910
C	32.2702353	25.4559767	26.6707224
C	33.4565180	24.9905763	27.2112919
H	32.6064433	28.6931591	27.6605776
H	34.7208439	27.7582931	28.5158319
H	31.6332638	24.7435459	26.1520348
H	33.7567341	23.9438763	27.1372495
C	29.5508767	33.5197360	22.8718364

C	30.1233157	34.7840992	22.7905998
N	30.6464444	35.3859955	23.8818743
C	30.0089171	33.5150342	25.2235940
C	30.5734295	34.7760563	25.0902943
H	29.1733871	33.0681833	21.9535932
H	30.1896991	35.3370543	21.8509574
H	29.9602899	33.0724881	26.2193684
H	30.9800127	35.3268928	25.9424521
O	28.5114819	27.4660771	23.2785956
O	26.8677594	29.1694242	26.2798477
H	25.9002580	29.1992148	26.3408984
C	31.3548504	36.6876535	23.7456207
H	31.0497666	37.1695143	22.8088939
H	31.0947937	37.3367522	24.5907994
H	32.4382673	36.4991338	23.7367936
C	21.3017857	30.3282793	18.3776690
H	20.3110752	30.4685530	18.8249475
H	21.6324486	31.2639064	17.9100093
H	21.2669334	29.5222427	17.6296454
C	22.7071691	21.1116352	27.4251178
H	21.9216413	21.0521685	26.6606534
H	23.2586709	20.1674534	27.4764065
H	22.2569268	21.3293094	28.4009902
C	35.5809535	25.3010081	28.4004378
H	35.4855154	24.2227630	28.5703517
H	36.3899258	25.5066384	27.6837484
H	35.7917229	25.8046194	29.3515235
H	27.1886333	28.9212118	27.1601567
Fe	27.7645608	28.2241713	24.5268062
C	30.2240609	27.0761215	20.5702673
C	30.4874283	25.7953431	19.9498695
C	31.7986792	25.3261269	19.9206601
C	32.8493303	26.0810665	20.4638086
C	32.6061471	27.3479765	21.0514221
C	31.3188711	27.8313283	21.1050570
H	32.0211495	24.3715806	19.4359971
H	33.8707843	25.6977003	20.4143318
H	33.4386990	27.9326113	21.4473736
H	31.1118506	28.8024016	21.5626798
C	29.3607967	25.0465281	19.2862159
H	28.9238005	24.3317458	20.0079021
H	29.7571834	24.4345619	18.4614687
C	28.8776468	27.5986751	20.6676308
H	28.8455441	28.6996133	20.5880807
H	28.6595923	27.5833331	21.9219651
C	28.2507376	25.9830606	18.8066270
H	27.4235994	25.3890872	18.3900489
H	28.6268864	26.6304651	17.9953734
C	27.7453950	26.8443586	19.9724632
H	26.9794295	27.5582918	19.6318803
H	27.2428519	26.1843735	20.7041235

^{2}IM(R)

N	26.9636933	29.9361537	23.8526755
N	25.8708422	27.4303237	24.4703476
N	28.1850705	26.8260146	26.0622485
N	29.3395735	29.2586672	25.2942311
C	25.7838365	30.0516763	23.1697209
C	27.4893899	31.2050290	23.9422511
C	27.3488071	25.8477375	26.5579487
C	29.3885798	26.6777443	26.7099209
C	25.0571817	27.7033915	23.3923162

C	25.5203694	26.1841463	24.9182298
C	29.6911257	30.4997200	24.8279514
C	30.5044413	28.6414126	25.6959102
C	25.4972474	31.4491362	22.9154453
C	26.5411120	32.1650665	23.4178989
C	28.0086825	25.1398592	27.6283521
C	29.2764242	25.6349693	27.7097608
C	24.2425354	26.5472664	23.0942901
C	24.4972145	25.6246729	24.0638987
C	31.1266902	30.6428021	24.8602789
C	31.6340533	29.4803886	25.3598945
C	24.9553148	28.9695085	22.7808537
C	28.8068521	31.4924566	24.3465914
C	30.5540257	27.4369396	26.4341504
C	26.0963881	25.5033349	26.0146454
H	24.5905296	31.8380404	22.4615975
H	26.6394697	33.2464658	23.4469601
H	27.5516064	24.3755484	28.2485147
H	30.0456474	25.3392122	28.4184702
H	23.5565518	26.4315029	22.2617132
H	24.0615081	24.6327339	24.1502762
H	31.6857243	31.5029975	24.5028689
H	32.6847366	29.2385221	25.4822842
C	23.9402740	29.2147493	21.7199593
C	29.3555826	32.8754315	24.1730012
C	31.8610178	26.9455383	26.9562743
C	25.3233252	24.3355577	26.5433612
C	24.2819908	29.9508583	20.5622989
C	23.3295634	30.2381032	19.6025151
N	22.0554796	29.7992164	19.7249150
C	22.6096738	28.7575374	21.7946420
C	21.6985350	29.0487990	20.7907145
H	25.2987441	30.3109554	20.4044841
H	23.5574170	30.8282301	18.7147807
H	22.2416988	28.2032154	22.6529615
H	20.6647447	28.7012118	20.8299905
C	23.9860754	24.5184231	26.9566444
C	23.2074267	23.4365068	27.3250967
N	23.7050177	22.1789990	27.2998428
C	25.8247514	23.0209433	26.5975896
C	24.9989456	21.9667180	26.9729249
H	23.5380739	25.5116501	26.9927401
H	22.1662009	23.5376502	27.6346622
H	26.8497814	22.7916453	26.3040307
H	25.3465357	20.9316994	26.9994198
C	32.8155937	27.7888164	27.5653404
C	34.0298195	27.2856950	28.0084017
N	34.3218328	25.9695715	27.8905547
C	32.2113898	25.5815172	26.8518285
C	33.4296168	25.1268753	27.3251784
H	32.6167934	28.8453519	27.7315359
H	34.7884488	27.9196664	28.4725369
H	31.5369544	24.8585512	26.3941992
H	33.7269334	24.0797449	27.2623248
C	29.3607552	33.5314338	22.9270585
C	29.9419630	34.7860971	22.7872695
N	30.5240023	35.4087278	23.8361742
C	29.9348324	33.5787535	25.2526022
C	30.5057036	34.8282395	25.0609666
H	28.9364475	33.0587360	22.0402891
H	29.9690738	35.3158728	21.8329255
H	29.9293800	33.1621366	26.2605694

H	30.9607105	35.3948081	25.8772880
O	28.3653065	27.5518322	23.3869215
O	26.7647200	29.2350195	26.5235208
H	25.8110540	29.4100438	26.4986139
C	31.2347393	36.6989879	23.6360246
H	30.9021635	37.1530640	22.6949596
H	31.0055253	37.3764956	24.4680663
H	32.3158694	36.5030501	23.5983488
C	21.0586939	30.1388156	18.6859290
H	20.0635266	30.1533009	19.1455863
H	21.2924997	31.1294843	18.2784040
H	21.0939718	29.3857270	17.8844861
C	22.7855591	21.0523001	27.5853465
H	22.0450256	20.9916259	26.7768457
H	23.3595821	20.1220171	27.6418898
H	22.2757140	21.2324330	28.5398951
C	35.6245885	25.4555723	28.3700105
H	35.5659791	24.3659112	28.4700901
H	36.4112232	25.7314684	27.6526290
H	35.8419885	25.9013116	29.3484954
H	26.9562382	28.8654664	27.3991942
Fe	27.6272459	28.3298990	24.8530858
C	30.2211456	26.2618039	20.3169829
C	30.8640752	25.4158901	19.3336918
C	32.2548212	25.3992901	19.2991189
C	32.9988455	26.2453670	20.1330319
C	32.3808526	27.1157784	21.0610607
C	31.0084415	27.1102524	21.1724077
H	32.7655584	24.7453950	18.5897795
H	34.0889916	26.2369983	20.0605920
H	32.9961237	27.7673957	21.6846042
H	30.4810014	27.7282252	21.9046621
C	30.0376613	24.5480062	18.4261959
H	30.0615764	23.5189240	18.8308670
H	30.5311713	24.4704052	17.4456277
C	28.8452466	26.1922044	20.4949985
H	28.4104685	26.7400968	21.3477181
H	28.9878715	26.8861188	23.7186569
C	28.5944500	25.0371047	18.2787375
H	27.9863259	24.2845477	17.7597357
H	28.5796330	25.9452010	17.6526245
C	27.9677757	25.3717975	19.6358653
H	26.9800573	25.8564441	19.5423439
H	27.7479113	24.4483987	20.2216098

^{2}TS2(R)

N	27.0266478	29.8880689	23.6912943
N	25.9579478	27.3663635	24.2783614
N	28.2940622	26.7376886	25.8298549
N	29.4172926	29.2012055	25.1093301
C	25.8236436	30.0158451	23.0438210
C	27.5425765	31.1615473	23.8114830
C	27.4715485	25.7353877	26.3024866
C	29.5007497	26.5880453	26.4744730
C	25.1054683	27.6641058	23.2349729
C	25.6058244	26.1170905	24.7215199
C	29.7528576	30.4652700	24.6862748
C	30.5859960	28.6009214	25.5300623
C	25.5249507	31.4158421	22.8368590
C	26.5768976	32.1253310	23.3344225
C	28.1485619	24.9955347	27.3393667
C	29.4067869	25.5083812	27.4362413

C	24.2694881	26.5201617	22.9508745
C	24.5448225	25.5836782	23.9005811
C	31.1828147	30.6382909	24.7663520
C	31.7019913	29.4753084	25.2535495
C	24.9842022	28.9424260	22.6537876
C	28.8586137	31.4553520	24.2181804
C	30.6509945	27.3805297	26.2398147
C	26.2056847	25.4104283	25.7851478
H	24.6048799	31.8108868	22.4165065
H	26.6680906	33.2061823	23.3930565
H	27.7103363	24.1945386	27.9267069
H	30.1814598	25.1986427	28.1320876
H	23.5573010	26.4202559	22.1390696
H	24.0994360	24.5965125	23.9928003
H	31.7328490	31.5211981	24.4527426
H	32.7527412	29.2526486	25.4060592
C	23.9310417	29.2083165	21.6331101
C	29.3887274	32.8513416	24.0766914
C	31.9557461	26.9093035	26.7914677
C	25.4297401	24.2478722	26.3300472
C	24.2279292	29.9696164	20.4798413
C	23.2433758	30.2632662	19.5544734
N	21.9792343	29.8075189	19.7090062
C	22.6078225	28.7390235	21.7428387
C	21.6633850	29.0352447	20.7711176
H	25.2339842	30.3468024	20.2965770
H	23.4357525	30.8703753	18.6695552
H	22.2698084	28.1684096	22.6027020
H	20.6365445	28.6713446	20.8344582
C	24.1484171	24.4670522	26.8767633
C	23.3635894	23.4011119	27.2819435

N	23.8026399	22.1278926	27.1581928
C	25.8779888	22.9149538	26.2791164
C	25.0472420	21.8787492	26.6942889
H	23.7480560	25.4750002	26.9917208
H	22.3634578	23.5310694	27.6994354
H	26.8594973	22.6603177	25.8762115
H	25.3499758	20.8305005	26.6438952
C	32.8626152	27.7503957	27.4701505
C	34.0630123	27.2522509	27.9580830
N	34.3822925	25.9449277	27.8191621
C	32.3396562	25.5581877	26.6564660
C	33.5369867	25.1055501	27.1813339
H	32.6381057	28.7979406	27.6603149
H	34.7867990	27.8841818	28.4773075
H	31.7018082	24.8424194	26.1414140
H	33.8517084	24.0638859	27.1017130
C	29.3944482	33.5301635	22.8433897
C	29.9652979	34.7927331	22.7287640
N	30.5363494	35.4004135	23.7918899
C	29.9494071	33.5425738	25.1734044
C	30.5113190	34.8002058	25.0068983
H	28.9782795	33.0721922	21.9451585
H	29.9933168	35.3394296	21.7836459
H	29.9392710	33.1097441	26.1745823
H	30.9552446	35.3566452	25.8363034
O	28.4585247	27.5105369	23.1511588
O	26.8639189	29.1136718	26.3433793
H	25.9041595	29.2561821	26.3347919
C	31.2438810	36.6969109	23.6174484
H	30.9113256	37.1673859	22.6844748
H	31.0115197	37.3572227	24.4622689

H	32.3254200	36.5033086	23.5774482
C	20.9477941	30.1566119	18.7072551
H	19.9635305	30.1155201	19.1877811
H	21.1378315	31.1723388	18.3402049
H	20.9945170	29.4404574	17.8733433
C	22.8692103	21.0218148	27.4814987
H	22.1170105	20.9538936	26.6840184
H	23.4309977	20.0851492	27.5543680
H	22.3760330	21.2326661	28.4379603
C	35.6681763	25.4345729	28.3474784
H	35.5924378	24.3505959	28.4896396
H	36.4721305	25.6720561	27.6352671
H	35.8706569	25.9168147	29.3113050
H	27.0826484	28.7273233	27.2058874
Fe	27.7124308	28.2655292	24.6592967
C	30.3470764	27.5412866	20.9176522
C	30.4606867	26.3356739	20.1441017
C	31.7318217	25.7990982	19.9378787
C	32.8694090	26.4321317	20.4539196
C	32.7666198	27.6139927	21.2135502
C	31.5189454	28.1578654	21.4509843
H	31.8490207	24.8867849	19.3473966
H	33.8558236	26.0078426	20.2522679
H	33.6666222	28.0970311	21.5953021
H	31.4171114	29.0749063	22.0372932
C	29.2149313	25.7087468	19.5737317
H	28.7622525	25.0533855	20.3426526
H	29.4721145	25.0549786	18.7273086
C	29.0820834	28.1445407	21.1060803
H	29.0543147	29.1175482	21.5959186
H	29.1772064	26.9144994	23.4154140
C	28.1951350	26.7749143	19.1651954
H	27.2679457	26.2975076	18.8184291
H	28.5895700	27.3641851	18.3202621
C	27.8759225	27.7004368	20.3473569
H	27.3076488	28.5908509	20.0292950
H	27.2298311	27.1793178	21.0765205

^{2}PS(R)

N	27.0367866	29.8459784	23.7610440
N	25.9535400	27.3440814	24.3663039
N	28.2920968	26.7106610	25.9144166
N	29.4138146	29.1657873	25.1973072
C	25.8369108	29.9773947	23.0993145
C	27.5624460	31.1201515	23.8679107
C	27.4701109	25.7018794	26.3819994
C	29.5066389	26.5501631	26.5529647
C	25.0665987	27.6547089	23.3503297
C	25.5791000	26.1065647	24.8401349
C	29.7529972	30.4345135	24.7799861
C	30.5842840	28.5718516	25.6366492
C	25.5615868	31.3728800	22.8583541
C	26.6139091	32.0804217	23.3589508
C	28.1581449	24.9422790	27.3923358
C	29.4185149	25.4533512	27.4888140
C	24.1817041	26.5396813	23.1228040
C	24.4677987	25.6049512	24.0713634
C	31.1775792	30.6142293	24.8870509
C	31.6956690	29.4549031	25.3817499
C	24.9755239	28.9166701	22.7341061
C	28.8706729	31.4191226	24.2877249
C	30.6550679	27.3454284	26.3303096

C	26.1955105	25.3880860	25.8827315
H	24.6539510	31.7689386	22.4124396
H	26.7180018	33.1611425	23.3952301
H	27.7257532	24.1252168	27.9617334
H	30.2022949	25.1264124	28.1663900
H	23.4287303	26.4559053	22.3468547
H	23.9910199	24.6365328	24.1965316
H	31.7266158	31.4986727	24.5779326
H	32.7452335	29.2375531	25.5472559
C	23.9500678	29.1783226	21.6827271
C	29.4058442	32.8130034	24.1254735
C	31.9679448	26.8665749	26.8619712
C	25.4263825	24.2173197	26.4266718
C	24.3166948	29.8566286	20.4989579
C	23.3698620	30.1500787	19.5347834
N	22.0804364	29.7699010	19.6820312
C	22.6030949	28.7844779	21.7874515
C	21.6988334	29.0748010	20.7752034
H	25.3465478	30.1663515	20.3194379
H	23.6154102	30.6983946	18.6253438
H	22.2167973	28.2785373	22.6677057
H	20.6535692	28.7664314	20.8282700
C	24.1888632	24.4353922	27.0638733
C	23.3965166	23.3643848	27.4437521
N	23.7896175	22.0919984	27.2103217
C	25.8371879	22.8810048	26.2711412
C	25.0014226	21.8413960	26.6673242
H	23.8230750	25.4438802	27.2624022
H	22.4233587	23.4924759	27.9207976
H	26.7881734	22.6272328	25.8001900
H	25.2712247	20.7912963	26.5346896

C	32.8715294	27.6943748	27.5603718
C	34.0918568	27.1973921	27.9994876
N	34.4315620	25.9044183	27.7920572
C	32.3639934	25.5270975	26.6707166
C	33.5832251	25.0755723	27.1461127
H	32.6349107	28.7303589	27.7959101
H	34.8175637	27.8193034	28.5288721
H	31.7247594	24.8192858	26.1458876
H	33.9133167	24.0438973	27.0165211
C	29.4547269	33.4499605	22.8715287
C	30.0237420	34.7119824	22.7394650
N	30.5540541	35.3547950	23.8029199
C	29.9252791	33.5375749	25.2197822
C	30.4898345	34.7922399	25.0339579
H	29.0752188	32.9611489	21.9729988
H	30.0826936	35.2297708	21.7798466
H	29.8840060	33.1350580	26.2330462
H	30.9045576	35.3744933	25.8608276
O	28.5771298	27.3703656	23.2178674
O	26.8717597	29.0763637	26.3950388
H	25.9047150	29.1699812	26.4008499
C	31.2620715	36.6494729	23.6097665
H	30.9210442	37.1118978	22.6756018
H	31.0387497	37.3172491	24.4509639
H	32.3430487	36.4536212	23.5609576
C	21.0918527	30.1189684	18.6370198
H	20.0948762	30.1386983	19.0915893
H	21.3359638	31.1095830	18.2350594
H	21.1295248	29.3675289	17.8341209
C	22.8400237	20.9882789	27.4942977
H	22.1035688	20.9431187	26.6801589

H	23.3923613	20.0453221	27.5605179
H	22.3297504	21.1849944	28.4442946
C	35.7457983	25.4005018	28.2544670
H	35.7085483	24.3075783	28.3179493
H	36.5242198	25.7156004	27.5438895
H	35.9545119	25.8178165	29.2465866
H	27.1358360	28.7175227	27.2579905
Fe	27.7000360	28.2422787	24.7594039
C	30.0444549	27.2135308	21.2764922
C	30.3070603	26.3228705	20.2098675
C	31.6452603	26.0078502	19.9199208
C	32.7050478	26.6094305	20.5958508
C	32.4425179	27.5495327	21.5991624
C	31.1222218	27.8380083	21.9389344
H	31.8601484	25.2758120	19.1381299
H	33.7333305	26.3607185	20.3247642
H	33.2667283	28.0514487	22.1102322
H	30.9199307	28.5679187	22.7255706
C	29.1965805	25.7497488	19.3487610
H	29.0986552	24.6655240	19.5271950
H	29.4840866	25.8402262	18.2890594
C	28.6233681	27.4980898	21.7248459
H	28.3511418	28.5521184	21.5585704
H	29.2763824	26.7514952	23.4884366
C	27.8462468	26.4235137	19.5961331
H	27.0365486	25.8355268	19.1380537
H	27.8227666	27.4204651	19.1190107
C	27.5966377	26.5598585	21.1003932
H	26.5798235	26.9208029	21.3077404
H	27.6906020	25.5674936	21.5752175

^{2}TS1(S)

N	26.8160401	30.0443277	23.9244974
N	25.7639872	27.4745520	24.3217947
N	28.0595198	26.7778002	25.8374874
N	29.2027131	29.2889172	25.2725903
C	25.6998587	30.1648888	23.1381910
C	27.3726371	31.3017152	24.0171592
C	27.2266726	25.7701271	26.2907939
C	29.2715931	26.5936170	26.4664898
C	24.9156054	27.8278306	23.2915836
C	25.3516884	26.2376972	24.7592068
C	29.5500988	30.5689905	24.9177612
C	30.3650803	28.6589030	25.6624362
C	25.4800447	31.5577727	22.8224495
C	26.4943813	32.2640340	23.3955119
C	27.9089591	24.9838539	27.2850886
C	29.1763349	25.4769278	27.3804122
C	24.0078403	26.7400982	23.0165067
C	24.2478768	25.7785521	23.9525166
C	30.9795913	30.7333260	25.0232427
C	31.4886257	29.5453326	25.4492939
C	24.8696749	29.1059173	22.7072523
C	28.6766457	31.5754945	24.4629182
C	30.4242414	27.3962218	26.2887062
C	25.9440421	25.4856778	25.7931491
H	24.6315621	31.9555534	22.2735584
H	26.6270596	33.3422182	23.3928681
H	27.4755429	24.1616056	27.8470781
H	29.9586174	25.1253194	28.0476939
H	23.2601852	26.6994154	22.2311592
H	23.7429356	24.8213245	24.0503317

H	31.5330146	31.6302905	24.7612791
H	32.5373075	29.3094221	25.5960371
C	23.9331520	29.3824161	21.5798211
C	29.2426155	32.9596191	24.3102315
C	31.7285585	26.8924737	26.8159745
C	25.1597740	24.3207557	26.3200790
C	24.4398972	29.9663716	20.3992392
C	23.5961616	30.2676175	19.3478649
N	22.2732350	29.9938402	19.4117134
C	22.5535632	29.1004231	21.6071155
C	21.7523697	29.4049830	20.5131013
H	25.5013578	30.1930141	20.2932206
H	23.9561302	30.7367319	18.4335669
H	22.0726172	28.6735734	22.4860823
H	20.6802636	29.1963995	20.5027691
C	23.8775386	24.5316175	26.8692142
C	23.0920630	23.4600478	27.2583577
N	23.5370100	22.1885011	27.1302574
C	25.6085656	22.9889550	26.2563257
C	24.7829334	21.9476533	26.6674526
H	23.4757488	25.5366697	26.9997768
H	22.0870091	23.5863315	27.6671575
H	26.5902406	22.7364659	25.8535582
H	25.0934838	20.9018317	26.6172076
C	32.6257619	27.6941954	27.5529755
C	33.8439050	27.1840609	27.9814498
N	34.1897383	25.9002945	27.7292627
C	32.1267679	25.5586733	26.5874918
C	33.3444875	25.0926468	27.0519159
H	32.3822112	28.7189141	27.8271363
H	34.5647318	27.7880330	28.5368778
H	31.4882455	24.8661002	26.0430096
H	33.6739335	24.0654232	26.8902200
C	29.4152804	33.5510436	23.0452060
C	30.0266104	34.7932477	22.9224314
N	30.4744426	35.4618449	24.0083526
C	29.6801328	33.7077316	25.4231243
C	30.2881690	34.9441784	25.2470503
H	29.1003569	33.0393055	22.1344773
H	30.1834584	35.2737165	21.9541626
H	29.5471421	33.3359696	26.4409207
H	30.6441535	35.5445577	26.0883025
O	28.2215649	27.9223706	23.2697242
O	26.6714772	29.1141209	26.5314597
H	25.7373767	29.3745330	26.5256455
C	31.2267334	36.7338297	23.8342865
H	30.9724340	37.1743255	22.8624990
H	30.9536495	37.4340986	24.6326022
H	32.3033014	36.5143822	23.8803842
C	21.4073219	30.3303526	18.2586096
H	20.3954828	30.5342505	18.6256128
H	21.8041068	31.2237567	17.7619822
H	21.3903400	29.4827566	17.5574758
C	22.6167820	21.0742686	27.4637398
H	21.8194019	21.0363105	26.7100299
H	23.1763424	20.1331710	27.4734796
H	22.1815972	21.2511648	28.4539194
C	35.5033558	25.3859053	28.1824968
H	35.4712250	24.2910988	28.2058564
H	36.2873884	25.7308289	27.4920714
H	35.7035386	25.7673985	29.1908703
H	26.8156522	28.6293629	27.3582681

Fe	27.5273258	28.3473723	24.6933221
C	29.5698968	25.8122174	21.1752525
C	29.5867597	25.3098161	19.8212521
C	30.8093532	25.2592754	19.1554312
C	31.9706092	25.7920564	19.7323713
C	31.9335964	26.3974703	21.0130328
C	30.7553162	26.4000266	21.7235530
H	30.8557571	24.7871594	18.1711751
H	32.9143116	25.7617871	19.1853330
H	32.8440472	26.8365050	21.4274400
H	30.7036736	26.8329167	22.7248623
C	28.3319669	24.7533369	19.1994017
H	28.3880231	23.6478144	19.2357695
H	28.3140981	25.0020445	18.1258921
C	28.3965185	25.6361982	22.0120390
H	28.1608730	26.7657134	22.4849126
H	28.6669952	25.2781531	23.0180233
C	27.0628501	25.2265491	19.9089733
H	26.1855718	24.7102725	19.4954314
H	26.9137785	26.3066505	19.7270648
C	27.1729671	24.9680134	21.4118651
H	26.2640868	25.2956282	21.9405022
H	27.2430373	23.8778813	21.5932014

^{2}IM(S)

N	26.9346521	30.0789967	23.9160000
N	25.8317082	27.5040285	24.3970221
N	28.1001281	26.8355032	25.9497519
N	29.2643483	29.3600708	25.3335761
C	25.8054425	30.1856711	23.1423999
C	27.4693105	31.3482047	24.0041612
C	27.2826184	25.8145143	26.3862081
C	29.3129995	26.6496436	26.5585264
C	25.0224444	27.8373952	23.3342260
C	25.4344926	26.2610679	24.8129177
C	29.6182872	30.6440288	24.9997779
C	30.4117806	28.7337316	25.7651283
C	25.5715388	31.5739572	22.8138282
C	26.5848922	32.2956313	23.3713409
C	27.9762693	25.0115444	27.3645040
C	29.2384599	25.5113722	27.4580944
C	24.1551779	26.7233022	23.0155179
C	24.3798691	25.7666852	23.9588662
C	31.0412909	30.8181548	25.1652716
C	31.5395141	29.6290398	25.6005201
C	24.9732430	29.1131971	22.7373820
C	28.7565620	31.6388176	24.4931948
C	30.4606993	27.4604426	26.3767134
C	26.0099505	25.5199349	25.8679781
H	24.7160275	31.9627445	22.2697269
H	26.7057880	33.3748914	23.3562839
H	27.5493054	24.1747309	27.9074763
H	30.0285595	25.1549357	28.1131151
H	23.4493333	26.6587437	22.1938412
H	23.9008838	24.7939010	24.0313392
H	31.5995380	31.7213020	24.9401568
H	32.5835208	29.4051673	25.7900974
C	24.0079998	29.3760975	21.6334749
C	29.3112453	33.0253803	24.3459687
C	31.7643774	26.9320738	26.8753901
C	25.2226519	24.3518359	26.3784940
C	24.4497072	30.0085507	20.4504050

C	23.5623302	30.2931095	19.4306596
N	22.2565411	29.9519246	19.5218036
C	22.6416777	29.0344266	21.6895488
C	21.7964756	29.3189374	20.6258815
H	25.4950349	30.2883114	20.3173519
H	23.8720825	30.8023748	18.5191448
H	22.2047406	28.5735229	22.5727842
H	20.7360688	29.0584282	20.6418266
C	23.9234004	24.5578248	26.8895960
C	23.1314815	23.4855797	27.2612717
N	23.5848428	22.2152142	27.1514641
C	25.6787621	23.0210833	26.3364174
C	24.8455739	21.9783778	26.7270278
H	23.5143968	25.5616442	27.0040837
H	22.1149793	23.6097527	27.6416350
H	26.6732266	22.7727159	25.9634298
H	25.1608495	20.9334743	26.6904580
C	32.6787521	27.6923822	27.6347024
C	33.8854311	27.1437606	28.0463726
N	34.2047558	25.8615139	27.7547391
C	32.1415594	25.6007309	26.5936943
C	33.3473657	25.0954192	27.0443096
H	32.4531986	28.7113874	27.9437192
H	34.6180429	27.7128523	28.6217719
H	31.4884372	24.9432620	26.0224712
H	33.6599997	24.0690366	26.8491217
C	29.4906942	33.6217783	23.0834707
C	30.0913761	34.8692227	22.9672547
N	30.5207490	35.5422825	24.0581642
C	29.7282544	33.7798422	25.4631981
C	30.3253258	35.0218224	25.2946353
H	29.1880523	33.1090448	22.1694352
H	30.2539445	35.3517576	22.0011181
H	29.5820040	33.4075863	26.4786041
H	30.6636832	35.6264179	26.1400587
O	28.3239870	27.7779029	23.3099404
O	26.7155782	29.1528030	26.5920643
H	25.9994892	29.7980959	26.4817402
C	31.2586333	36.8234046	23.8958430
H	31.0413696	37.2428886	22.9060893
H	30.9376522	37.5330423	24.6676552
H	32.3352634	36.6245782	23.9959597
C	21.3419041	30.2566619	18.3992124
H	20.3379373	30.4349013	18.8010395
H	21.6971078	31.1550410	17.8807220
H	21.3218421	29.4037411	17.7046896
C	22.6583581	21.1000148	27.4615256
H	21.8725317	21.0687560	26.6953816
H	23.2161619	20.1578846	27.4731452
H	22.2079044	21.2693488	28.4463920
C	35.4999849	25.3002721	28.2005871
H	35.4256719	24.2077823	28.2303490
H	36.2928777	25.6130871	27.5052953
H	35.7203817	25.6750657	29.2068356
H	26.4028045	28.5066776	27.2443631
Fe	27.5713019	28.4157853	24.8367048
C	30.2110093	25.9311892	20.5647806
C	30.8214385	25.1494269	19.5103489
C	32.2101368	25.1087864	19.4526722
C	32.9831368	25.8911463	20.3204692
C	32.3972428	26.7023420	21.3198084
C	31.0283191	26.6997757	21.4664270

H	32.6998179	24.4869521	18.7009797
H	34.0708904	25.8732144	20.2209727
H	33.0345015	27.2966021	21.9772837
H	30.5261197	27.2524184	22.2654207
C	29.9618598	24.3623905	18.5611014
H	29.9722331	23.3092736	18.8971460
H	30.4308581	24.3360174	17.5665891
C	28.8382357	25.8717512	20.7646171
H	28.5218904	28.5594755	22.7701102
H	28.4288741	26.3829751	21.6523372
C	28.5274695	24.8887500	18.4750067
H	27.8959679	24.1915735	17.9091922
H	28.5243425	25.8422768	17.9202187
C	27.9304090	25.1299048	19.8653897
H	26.9482018	25.6324751	19.8282068
H	27.7096342	24.1673913	20.3834422

^{2}TS2(S)

N	26.8903611	30.0892421	23.9331679
N	25.7745032	27.5124792	24.4071895
N	28.0645285	26.8097878	25.9073711
N	29.2386604	29.3368234	25.2860163
C	25.7635918	30.1993085	23.1562457
C	27.4419005	31.3519753	24.0058252
C	27.2384343	25.8015364	26.3668196
C	29.2765966	26.6304768	26.5265230
C	24.9539586	27.8602768	23.3574275
C	25.3668066	26.2733435	24.8278435
C	29.5966620	30.6253965	24.9688394
C	30.3836522	28.7058466	25.7208353
C	25.5472616	31.5863916	22.8121375
C	26.5677079	32.3025751	23.3639848
C	27.9276063	25.0156389	27.3607756
C	29.1927182	25.5119131	27.4470107
C	24.0639818	26.7611263	23.0522963
C	24.2905698	25.7982314	23.9898273
C	31.0191877	30.7951798	25.1424509
C	31.5130281	29.6014276	25.5681590
C	24.9224504	29.1335000	22.7550003
C	28.7376713	31.6299459	24.4791263
C	30.4288225	27.4354821	26.3402825
C	25.9539578	25.5186731	25.8687874
H	24.6981226	31.9784371	22.2601110
H	26.7030503	33.3798312	23.3359244
H	27.4983549	24.1947407	27.9270426
H	29.9792666	25.1640230	28.1110880
H	23.3406453	26.7118017	22.2450285
H	23.7961121	24.8339116	24.0696265
H	31.5809314	31.7002209	24.9341949
H	32.5551907	29.3742377	25.7615044
C	23.9776442	29.3970561	21.6317013
C	29.3017709	33.0149221	24.3312080
C	31.7307726	26.9065182	26.8493902
C	25.1662914	24.3546888	26.3878102
C	24.4621645	29.9809878	20.4410665
C	23.6034185	30.2652109	19.3970413
N	22.2848954	29.9742291	19.4759873
C	22.6008315	29.1010521	21.6741760
C	21.7840308	29.3873134	20.5877011
H	25.5185955	30.2224474	20.3208742
H	23.9471129	30.7342914	18.4763434
H	22.1347520	28.6757710	22.5612415

H	20.7146216	29.1656521	20.5911165
C	23.8592472	24.5598639	26.8801503
C	23.0708420	23.4886489	27.2618466
N	23.5343682	22.2197682	27.1817055
C	25.6305859	23.0258794	26.3739840
C	24.8008059	21.9842595	26.7749905
H	23.4408562	25.5619246	26.9729632
H	22.0492543	23.6125429	27.6281575
H	26.6300989	22.7745549	26.0175155
H	25.1251549	20.9415052	26.7622675
C	32.6497121	27.6691020	27.6009306
C	33.8571715	27.1203100	28.0115356
N	34.1729886	25.8353373	27.7281979
C	32.1013408	25.5706156	26.5845444
C	33.3085749	25.0648954	27.0319835
H	32.4298955	28.6912766	27.9027130
H	34.5929211	27.6929184	28.5797355
H	31.4434750	24.9066276	26.0277435
H	33.6143838	24.0349948	26.8438273
C	29.5061931	33.5980760	23.0667745
C	30.1101984	34.8443354	22.9503348
N	30.5197838	35.5269223	24.0426789
C	29.6991458	33.7780686	25.4490340
C	30.3017886	35.0179240	25.2798929
H	29.2191838	33.0774563	22.1521015
H	30.2896094	35.3189873	21.9833737
H	29.5348743	33.4157157	26.4653205
H	30.6265304	35.6292542	26.1259118
O	28.2059725	27.8055747	23.2433308
O	26.7115315	29.1376211	26.5923397
H	26.0488501	29.8396119	26.4913391
C	31.2601542	36.8075744	23.8810742
H	31.0469005	37.2260134	22.8899117
H	30.9368046	37.5181471	24.6508785
H	32.3362248	36.6078617	23.9859466
C	21.4012979	30.2881423	18.3305146
H	20.3924223	30.4873074	18.7085034
H	21.7836855	31.1777790	17.8162499
H	21.3818451	29.4318292	17.6402525
C	22.6139247	21.1045971	27.5096487
H	21.8152930	21.0719519	26.7571093
H	23.1723266	20.1628736	27.5123835
H	22.1806530	21.2758780	28.5019177
C	35.4727647	25.2768808	28.1671693
H	35.4049699	24.1837896	28.1832242
H	36.2623173	25.6026108	27.4739108
H	35.6916403	25.6419876	29.1774287
H	26.3300892	28.4908710	27.2067522
Fe	27.5253637	28.4166976	24.8161092
C	29.8787532	25.9845377	21.3251009
C	29.9179060	25.4599934	19.9851130
C	31.1512215	25.4369427	19.3330363
C	32.2819938	26.0251600	19.9086440
C	32.2276849	26.6257699	21.1859109
C	31.0487419	26.5782471	21.8982359
H	31.2242739	24.9474209	18.3606315
H	33.2239537	26.0348009	19.3554014
H	33.1220584	27.0942450	21.6019467
H	30.9762098	26.9976103	22.9043827
C	28.6857998	24.8618822	19.3548406
H	28.7643392	23.7588014	19.4074007
H	28.6777230	25.0934777	18.2776168

C	28.7486423	25.7247942	22.1149705
H	28.5554559	28.5444226	22.7226968
H	28.8518514	25.7676078	23.1949464
C	27.3969267	25.3331282	20.0309382
H	26.5347646	24.7702849	19.6455354
H	27.2197350	26.3959663	19.7906816
C	27.4964214	25.1803556	21.5498616
H	26.6388354	25.6232891	22.0825340
H	27.4766753	24.1085611	21.8480855

^{2}PS(S)

N	26.9159418	30.0292098	23.9556236
N	25.7647401	27.4831968	24.4850186
N	28.0580195	26.7672702	25.9605353
N	29.2495324	29.2706495	25.3127780
C	25.7958100	30.1385557	23.1636248
C	27.4842269	31.2898188	24.0036053
C	27.2385743	25.7392733	26.3993640
C	29.2857792	26.5616781	26.5517076
C	24.9196287	27.8408210	23.4525253
C	25.3242132	26.2598496	24.9385670
C	29.6135675	30.5686467	25.0105874
C	30.3880722	28.6468705	25.7861818
C	25.6118185	31.5151834	22.7737851
C	26.6343570	32.2311886	23.3231262
C	27.9515666	24.9092742	27.3324074
C	29.2188415	25.4025856	27.4151659
C	23.9759119	26.7768957	23.2074208
C	24.1984567	25.8222271	24.1540091
C	31.0241095	30.7436602	25.2379868
C	31.5102093	29.5517379	25.6785246
C	24.9239182	29.0877053	22.8017972
C	28.7720698	31.5709390	24.4922603
C	30.4352654	27.3686962	26.3838130
C	25.9376803	25.4790231	25.9384138
H	24.7793599	31.9032406	22.1940782
H	26.7880188	33.3045012	23.2653374
H	27.5347744	24.0578824	27.8616799
H	30.0213246	25.0224457	28.0409554
H	23.2228468	26.7420798	22.4269435
H	23.6703517	24.8798171	24.2671641
H	31.5889778	31.6519599	25.0553296
H	32.5446396	29.3311021	25.9144240
C	23.9890509	29.3352779	21.6634253
C	29.3412835	32.9547510	24.3293738
C	31.7447635	26.8268197	26.8676431
C	25.1676058	24.2918759	26.4379404
C	24.5109042	29.8013093	20.4380312
C	23.6703253	30.0692685	19.3743698
N	22.3352723	29.8765174	19.4738353
C	22.5979439	29.1310814	21.7261537
C	21.7994597	29.3980734	20.6198056
H	25.5815386	29.9592108	20.3035845
H	24.0421552	30.4434025	18.4214374
H	22.1077336	28.7913182	22.6377200
H	20.7186009	29.2438609	20.6327999
C	23.9083587	24.4748643	27.0447798
C	23.1289444	23.3830090	27.3897451
N	23.5599288	22.1210106	27.1625951
C	25.6067264	22.9655935	26.2728693
C	24.7884604	21.9040683	26.6445457
H	23.5162629	25.4717222	27.2502881

H	22.1376822	23.4877233	27.8359994
H	26.5713669	22.7359244	25.8177005
H	25.0888895	20.8621927	26.5144105
C	32.6504240	27.5560409	27.6647844
C	33.8688586	27.0019225	28.0363755
N	34.2061087	25.7458337	27.6642665
C	32.1329820	25.5181699	26.5146502
C	33.3527334	25.0067427	26.9229062
H	32.4168104	28.5553249	28.0270685
H	34.5968807	27.5479245	28.6400010
H	31.4851951	24.8823892	25.9143640
H	33.6779330	23.9987177	26.6628965
C	29.5889118	33.5019569	23.0566700
C	30.1821031	34.7523967	22.9273181
N	30.5437733	35.4704977	24.0136043
C	29.6903671	33.7521130	25.4383550
C	30.2882636	34.9934083	25.2556658
H	29.3417986	32.9537929	22.1462974
H	30.3897715	35.2022152	21.9543203
H	29.4922878	33.4196624	26.4587451
H	30.5777614	35.6301219	26.0958240
O	28.2297796	27.7440442	23.1761967
O	26.7412496	29.1096026	26.5977408
H	26.1024578	29.8319730	26.4740997
C	31.2741396	36.7562102	23.8379355
H	31.0331735	37.1775278	22.8540765
H	30.9680566	37.4613034	24.6195710
H	32.3534883	36.5602684	23.9135059
C	21.4701138	30.1848027	18.3114992
H	20.4607119	30.4045072	18.6748973
H	21.8713391	31.0616811	17.7896678
H	21.4462607	29.3185172	17.6340556
C	22.6417861	20.9893692	27.4404842
H	21.8736097	20.9578573	26.6562649
H	23.2133614	20.0553488	27.4526357
H	22.1684278	21.1404154	28.4170169
C	35.5220385	25.1855753	28.0517685
H	35.4664040	24.0926779	28.0218497
H	36.2932803	25.5509567	27.3571958
H	35.7565534	25.5077890	29.0724062
H	26.3256091	28.4687927	27.1983673
Fe	27.5182520	28.3669766	24.8858490
C	29.8793526	26.5392470	21.8256598
C	29.8233959	26.1814431	20.4606545
C	31.0123627	26.2108011	19.7176750
C	32.2188217	26.6350344	20.2723918
C	32.2585932	27.0445851	21.6118266
C	31.0991060	26.9834700	22.3794741
H	30.9716087	25.8806119	18.6791357
H	33.1260666	26.6491869	19.6634021
H	33.1988527	27.3789920	22.0553019
H	31.1405230	27.2493150	23.4396669
C	28.5390176	25.7308979	19.7948002
H	28.5853884	24.6395210	19.6245923
H	28.4740980	26.1773454	18.7892425
C	28.6795373	26.3720310	22.7218828
H	28.7859429	28.4071922	22.7334602
H	28.9715526	25.8946535	23.6636726
C	27.2982009	26.0636773	20.6213272
H	26.4125311	25.5624071	20.2021140
H	27.0964356	27.1505471	20.5826064
C	27.5146679	25.6354643	22.0730877

H	26.6092141	25.7621212	22.6788499
H	27.7541612	24.5587635	22.1019787

[6]RS

N	26.9050244	30.0814126	23.8956096
N	25.7055420	27.5070567	24.4652202
N	28.1027825	26.7646163	26.0285394
N	29.3041886	29.3321924	25.4305601
C	25.7660871	30.1985659	23.1611038
C	27.4919850	31.3209405	23.9717762
C	27.2697400	25.7365731	26.4051498
C	29.3095045	26.5754530	26.6395299
C	24.9062178	27.8478208	23.3970656
C	25.3565913	26.2472642	24.8557959
C	29.6647884	30.5789471	25.0007224
C	30.4424169	28.6843550	25.8502449
C	25.5635773	31.5958063	22.8235531
C	26.6125162	32.2913835	23.3478460
C	27.9634937	24.8954494	27.3559839
C	29.2219728	25.4007419	27.4943136
C	24.0534206	26.7206474	23.0681583
C	24.3035476	25.7500765	23.9928480
C	31.1010512	30.7216342	25.1120085
C	31.5859470	29.5462882	25.6060278
C	24.8970437	29.1249630	22.7919919
C	28.7959839	31.5684079	24.4562571
C	30.4598929	27.4109120	26.4782809
C	25.9825574	25.4848875	25.8835863
H	24.7046427	32.0075882	22.3018882
H	26.7542723	33.3677657	23.3233214
H	27.5372445	24.0355327	27.8614472
H	29.9974363	25.0211523	28.1538573
H	23.3471699	26.6508222	22.2469427
H	23.8451151	24.7658911	24.0412350
H	31.6793785	31.5894356	24.8074729
H	32.6316335	29.3074310	25.7715347
C	23.9524905	29.3891900	21.6705799
C	29.3699713	32.9454233	24.2911943
C	31.7616163	26.8955973	26.9985295
C	25.1988483	24.3014388	26.3689615
C	24.4254286	30.0050551	20.4910010
C	23.5624415	30.2843454	19.4494247
N	22.2527539	29.9535762	19.5165579
C	22.5832291	29.0574278	21.7027269
C	21.7635164	29.3362255	20.6170791
H	25.4761068	30.2722643	20.3765739
H	23.8959646	30.7803542	18.5390208
H	22.1256271	28.6080022	22.5818484
H	20.7014783	29.0816608	20.6107359
C	23.9165811	24.5014821	26.9221873
C	23.1226897	23.4229699	27.2717507
N	23.5586331	22.1537421	27.0981651
C	25.6382315	22.9694610	26.2580538
C	24.8038402	21.9207124	26.6284492
H	23.5232526	25.5046413	27.0886661
H	22.1180939	23.5425395	27.6834246
H	26.6183371	22.7262361	25.8461425
H	25.1055679	20.8749956	26.5397131
C	32.6642260	27.6881420	27.7393375
C	33.8762430	27.1663030	28.1710841
N	34.2088980	25.8780352	27.9260494
C	32.1596447	25.5627739	26.7556959

C	33.3653875	25.0822185	27.2322455
H	32.4283145	28.7150965	28.0131748
H	34.6033918	27.7627215	28.7248563
H	31.5288826	24.8810347	26.1904090
H	33.6863687	24.0520965	27.0688209
C	29.4929162	33.5575146	23.0289824
C	30.0886399	34.8062406	22.9017626
N	30.5694385	35.4649479	23.9797453
C	29.8460304	33.6830173	25.3967649
C	30.4354318	34.9267712	25.2166242
H	29.1494832	33.0563311	22.1229822
H	30.2073505	35.3017939	21.9358029
H	29.7498849	33.2982348	26.4134298
H	30.8143383	35.5200228	26.0527542
O	28.2525832	27.7460460	23.4148633
O	26.7163626	29.1897423	26.5628959
H	25.7550767	29.1058795	26.6606952
C	31.3020238	36.7461088	23.7974202
H	31.0068531	37.1998319	22.8434674
H	31.0540613	37.4306323	24.6175641
H	32.3816595	36.5368283	23.7978481
C	21.3656086	30.2530008	18.3704926
H	20.3585106	30.4607732	18.7496363
H	21.7487610	31.1328461	17.8400740
H	21.3415144	29.3857892	17.6939909
C	22.6309904	21.0341962	27.3877555
H	21.8623843	20.9990745	26.6041200
H	23.1943602	20.0956225	27.4111065
H	22.1582663	21.2002834	28.3624027
C	35.5052070	25.3421707	28.3995631
H	35.4263863	24.2541244	28.5034771
H	36.2927341	25.6033049	27.6771525
H	35.7356209	25.7847682	29.3756889
H	27.1018128	28.9478020	27.4182077
Fe	27.6021917	28.3363113	24.7609619
C	30.1643074	26.3308485	20.1443090
C	30.8078485	25.2265136	19.4411473
C	32.2008424	25.0439422	19.5711112
C	32.9613990	25.9432891	20.2908510
C	32.3361143	27.0585766	20.9378252
C	30.9607146	27.2324189	20.8699437
H	32.6714296	24.2077244	19.0514724
H	34.0436780	25.8236559	20.3594620
H	32.9579608	27.7671061	21.4912690
H	30.4792976	28.0585767	21.3983960
C	30.0084854	24.2832581	18.6047085
H	30.0195785	23.3108302	19.1408300
H	30.5661108	24.0563197	17.6787421
C	28.6784386	26.5152533	20.0654014
H	28.5047304	27.3552347	19.3573231
H	28.3135867	26.8806837	21.0415914
C	28.5669283	24.7092303	18.3207071
H	28.0018087	23.8463450	17.9453210
H	28.5572114	25.4654658	17.5168435
C	27.9098415	25.2834457	19.5742207
H	26.8623503	25.5568746	19.3745190
H	27.8852931	24.5152731	20.3656595

^{6}TS1(R)

N	26.9818695	29.9085114	23.7008023
N	25.8005141	27.3295744	24.2309232
N	28.1960323	26.5470182	25.7589567

N	29.3926995	29.1156908	25.1759485
C	25.8323362	30.0460274	22.9762418
C	27.5697162	31.1449579	23.7976140
C	27.3692216	25.5086425	26.1189079
C	29.4099664	26.3444687	26.3628679
C	24.9477987	27.7081213	23.2191974
C	25.4154473	26.0839519	24.6497964
C	29.7336081	30.4028251	24.8404906
C	30.5160508	28.4999883	25.6721769
C	25.6382100	31.4476996	22.6617313
C	26.6950236	32.1280820	23.1928351
C	28.0760412	24.6329596	27.0256936
C	29.3347598	25.1370683	27.1682949
C	24.0256430	26.6239463	22.9479832
C	24.2873861	25.6420528	23.8577443
C	31.1471897	30.5925077	25.0846997
C	31.6360325	29.4168103	25.5717952
C	24.9398516	28.9928922	22.6257837
C	28.8669197	31.3911378	24.3069844
C	30.5393267	27.2077899	26.2633740
C	26.0579304	25.2943185	25.6375420
H	24.7789616	31.8747201	22.1525448
H	26.8430409	33.2035820	23.1863117
H	27.6629193	23.7558013	27.5147508
H	30.1154339	24.7334528	27.8059800
H	23.2623555	26.5901990	22.1773861
H	23.7836922	24.6826307	23.9407476
H	31.7118803	31.4989633	24.8901133
H	32.6682487	29.2174033	25.8389432
C	23.9539904	29.2825660	21.5455408
C	29.4124773	32.7891056	24.2028470

C	31.8237062	26.7123543	26.8473613
C	25.2551345	24.1402598	26.1630527
C	24.3983368	29.8623157	20.3372614
C	23.5029156	30.1536080	19.3270818
N	22.1853169	29.8779601	19.4569237
C	22.5767518	29.0041872	21.6421132
C	21.7219528	29.2970165	20.5873363
H	25.4521040	30.0905862	20.1756762
H	23.8162024	30.6101414	18.3904919
H	22.1405647	28.5846582	22.5464694
H	20.6530906	29.0799912	20.6288773
C	24.0264787	24.3955134	26.8070573
C	23.2118861	23.3527386	27.2133488
N	23.5778932	22.0662713	27.0087835
C	25.6250312	22.7913579	26.0174101
C	24.7740288	21.7799074	26.4493534
H	23.6925808	25.4155044	26.9998344
H	22.2453770	23.5156017	27.6960919
H	26.5608092	22.5071866	25.5344320
H	25.0215833	20.7220950	26.3393432
C	32.6277205	27.4763399	27.7176034
C	33.8036929	26.9472931	28.2358250
N	34.1872678	25.6819242	27.9545943
C	32.2870003	25.4121826	26.5503362
C	33.4443125	24.9192507	27.1215629
H	32.3416934	28.4801879	28.0309542
H	34.4603228	27.5184183	28.8918691
H	31.7368705	24.7635379	25.8746176
H	33.8034929	23.9066716	26.9281860
C	29.6058689	33.4222103	22.9605850
C	30.1672296	34.6916590	22.8912496

N	30.5444624	35.3519380	24.0086765
C	29.7761771	33.5286181	25.3483008
C	30.3358124	34.7937866	25.2260896
H	29.3384532	32.9256843	22.0270602
H	30.3365731	35.2054328	21.9428906
H	29.6123895	33.1300097	26.3509048
H	30.6304182	35.3885602	26.0947270
O	28.3371083	27.5465829	23.0396530
O	26.8272594	28.9863122	26.3638708
H	25.8611267	29.0714677	26.3674373
C	31.2421634	36.6612848	23.8945297
H	30.9931458	37.1221728	22.9307469
H	30.9176276	37.3214986	24.7073580
H	32.3261570	36.4909326	23.9642828
C	21.2612457	30.2033336	18.3469015
H	20.2603316	30.3680886	18.7611309
H	21.6070198	31.1171049	17.8476011
H	21.2392640	29.3660701	17.6334144
C	22.6241384	20.9873463	27.3607643
H	21.8250032	20.9608253	26.6081054
H	23.1548896	20.0300293	27.3869669
H	22.1950672	21.1935445	28.3475256
C	35.4283197	25.1252657	28.5390242
H	35.2721446	24.0626802	28.7603583
H	36.2557381	25.2563704	27.8261528
H	35.6537204	25.6592205	29.4688289
H	27.0541169	28.5311612	27.1891024
Fe	27.7150259	28.1188308	24.4661678
C	30.3006567	26.6007259	20.3314503
C	30.4258807	25.2881444	19.7150171
C	31.6779840	24.6838729	19.6866495
C	32.7968775	25.3300853	20.2328106
C	32.6976183	26.6411329	20.7818412
C	31.4745776	27.2609191	20.8299973
H	31.7949699	23.7017598	19.2214180
H	33.7646376	24.8246789	20.2304377
H	33.5947405	27.1390336	21.1538269
H	31.3712774	28.2602120	21.2599713
C	29.2182527	24.6491970	19.0884388
H	28.7432951	23.9784344	19.8311147
H	29.5366943	23.9951907	18.2622465
C	29.0057390	27.2376370	20.4726177
H	29.0874417	28.3353869	20.3692712
H	28.7874501	27.2782917	21.6763444
C	28.1927849	25.6856458	18.6242018
H	27.3058812	25.1715611	18.2253321
H	28.6180316	26.2807759	17.7974439
C	27.7930610	26.6066940	19.7835401
H	27.1200344	27.4055663	19.4350466
H	27.2132592	26.0253336	20.5222933

^{6}IM(R)

N	27.0324788	29.8992774	23.9656482
N	25.8670778	27.2814449	24.3990894
N	28.2410012	26.4779137	25.9609060
N	29.3708590	29.1565374	25.5885850
C	25.8884517	30.0369860	23.2248231
C	27.5991431	31.1447254	24.0937838
C	27.4077423	25.4373610	26.3018052
C	29.4187522	26.3148249	26.6368227
C	25.0300754	27.6787409	23.3848387
C	25.4932343	26.0223780	24.7778551

C	29.7305537	30.4321996	25.2225120
C	30.4996060	28.5218695	26.0523895
C	25.6842959	31.4388807	22.9302362
C	26.7267435	32.1243723	23.4861710
C	28.0863645	24.5829323	27.2513518
C	29.3250758	25.1168199	27.4532614
C	24.1349934	26.5862888	23.0572712
C	24.3944347	25.5815789	23.9431484
C	31.1492699	30.6057812	25.4456877
C	31.6286038	29.4257916	25.9304695
C	25.0174286	28.9822844	22.8265284
C	28.8831096	31.4063766	24.6349755
C	30.5362874	27.2040395	26.5915247
C	26.1154166	25.2179443	25.7726515
H	24.8303130	31.8643834	22.4118235
H	26.8625531	33.2019486	23.4942770
H	27.6685483	23.7103637	27.7470743
H	30.0741596	24.7492094	28.1474209
H	23.3924132	26.5597950	22.2664803
H	23.9091466	24.6099065	23.9803803
H	31.7276006	31.4992233	25.2317529
H	32.6646957	29.2129436	26.1703570
C	24.0455831	29.2899858	21.7394735
C	29.4401752	32.7921608	24.4701897
C	31.8252217	26.6907261	27.1415825
C	25.2920228	24.0650364	26.2609206
C	24.4863558	29.9348628	20.5619908
C	23.5956237	30.2399976	19.5507052
N	22.2874163	29.9077624	19.6450025
C	22.6754594	28.9671713	21.8017754
C	21.8274027	29.2694196	20.7457206
H	25.5342038	30.2039305	20.4261055
H	23.9047328	30.7563941	18.6424099
H	22.2395558	28.5046435	22.6839944
H	20.7656280	29.0159930	20.7633662
C	24.0084897	24.3223863	26.7898495
C	23.1706950	23.2847873	27.1549100
N	23.5632211	21.9967275	27.0201017
C	25.6835246	22.7160621	26.1895746
C	24.8057615	21.7082312	26.5750356
H	23.6557029	25.3438094	26.9292636
H	22.1669169	23.4503964	27.5535472
H	26.6594063	22.4264422	25.7984541
H	25.0712112	20.6505926	26.5187809
C	32.6866642	27.4444053	27.9677234
C	33.8790594	26.9000502	28.4267693
N	34.2298385	25.6281360	28.1314734
C	32.2491167	25.3766302	26.8398012
C	33.4292079	24.8710481	27.3491294
H	32.4357037	28.4546078	28.2895876
H	34.5788463	27.4630498	29.0441682
H	31.6467012	24.7287742	26.2084273
H	33.7648331	23.8527330	27.1473170
C	29.6400198	33.3590904	23.1961156
C	30.2068825	34.6197758	23.0614374
N	30.5845196	35.3371830	24.1437022
C	29.8075626	33.5889344	25.5750766
C	30.3729108	34.8436266	25.3880631
H	29.3748733	32.8134122	22.2894818
H	30.3806014	35.0805840	22.0871035
H	29.6373546	33.2462046	26.5967580
H	30.6677342	35.4819011	26.2251434

O	28.6560666	27.4474431	23.2378382
O	26.6630291	28.7160560	26.7593624
H	25.8139026	29.1821693	26.7242555
C	31.2837014	36.6377158	23.9625321
H	31.0324684	37.0504811	22.9777931
H	30.9618234	37.3385142	24.7416932
H	32.3676861	36.4699188	24.0384647
C	21.3686185	30.2286867	18.5303711
H	20.3648470	30.3944318	18.9383912
H	21.7191757	31.1371823	18.0266390
H	21.3499969	29.3883063	17.8205021
C	22.5913906	20.9227032	27.3314399
H	21.8254407	20.8972662	26.5450940
H	23.1166120	19.9637451	27.3813614
H	22.1203470	21.1315563	28.2989279
C	35.4956359	25.0602627	28.6444167
H	35.3577357	23.9880286	28.8241079
H	36.2951844	25.2292683	27.9081790
H	35.7500026	25.5542059	29.5887288
H	27.1759325	29.1669177	27.4463859
Fe	27.7543747	28.1146949	24.7502040
C	30.2222845	26.1539272	20.2255149
C	30.7997254	25.2329758	19.2706439
C	32.1782558	25.0515112	19.2985325
C	32.9812497	25.8252181	20.1462764
C	32.4354657	26.7847625	21.0289536
C	31.0689208	26.9344340	21.0905557
H	32.6412325	24.3297491	18.6229413
H	34.0635122	25.6857535	20.1220386
H	33.0988675	27.3757959	21.6629474
H	30.5881958	27.6099170	21.8034474
C	29.9127099	24.4602496	18.3346465
H	29.8432776	23.4230420	18.7082182
H	30.4073970	24.3634410	17.3574289
C	28.8450581	26.1947328	20.3926855
H	28.4576744	26.7814671	21.2410738
H	29.1083922	26.6196662	23.4580173
C	28.5182794	25.0692821	18.1838022
H	27.8546597	24.3809231	17.6445780
H	28.5845967	25.9892629	17.5795129
C	27.9134238	25.4257940	19.5430831
H	26.9525858	25.9620087	19.4569502
H	27.6548265	24.5094433	20.1236578

^{6}TS2(R)

N	27.0214322	29.8339296	23.7307042
N	25.8563255	27.2180604	24.1693304
N	28.2752788	26.3826490	25.6475072
N	29.3917568	29.0587069	25.2762072
C	25.8470817	29.9890614	23.0439006
C	27.5823562	31.0800989	23.8846715
C	27.4394225	25.3510174	26.0095683
C	29.4575181	26.2175835	26.3184716
C	24.9795883	27.6342318	23.1971567
C	25.4772516	25.9650185	24.5620539
C	29.7402963	30.3462718	24.9419916
C	30.5211660	28.4321926	25.7478228
C	25.6223727	31.3986402	22.8062940
C	26.6809783	32.0729077	23.3457357
C	28.1248361	24.4948062	26.9521174
C	29.3689470	25.0201662	27.1361998
C	24.0519875	26.5574190	22.9072997

C	24.3332367	25.5469101	23.7788981
C	31.1557032	30.5283665	25.1743829
C	31.6422438	29.3463590	25.6490871
C	24.9571040	28.9443247	22.6558346
C	28.8771990	31.3342985	24.4019249
C	30.5641001	27.1168736	26.2901008
C	26.1268229	25.1482239	25.5267180
H	24.7439139	31.8348368	22.3401779
H	26.8079102	33.1506635	23.3901601
H	27.7053484	23.6295980	27.4587388
H	30.1239952	24.6476200	27.8208305
H	23.2745570	26.5434736	22.1508577
H	23.8329513	24.5842713	23.8386924
H	31.7261573	31.4304556	24.9767027
H	32.6766701	29.1383873	25.9004219
C	23.9531561	29.2765564	21.6067417
C	29.4206366	32.7308609	24.2905918
C	31.8385791	26.6390856	26.9028636
C	25.3051728	24.0119812	26.0566873
C	24.3607186	29.9588769	20.4381876
C	23.4460109	30.2835479	19.4554885
N	22.1447793	29.9309505	19.5649257
C	22.5865062	28.9427634	21.6906348
C	21.7149538	29.2591793	20.6578997
H	25.4019146	30.2444167	20.2872089
H	23.7304892	30.8303410	18.5575968
H	22.1710664	28.4587225	22.5713629
H	20.6578173	28.9894827	20.6883294
C	24.0556578	24.2951918	26.6489515
C	23.2202389	23.2743828	27.0642694
N	23.5836527	21.9792567	26.9189360

C	25.6700163	22.6563028	25.9742009
C	24.7966370	21.6662188	26.4127452
H	23.7289472	25.3236252	26.8002001
H	22.2410438	23.4590278	27.5125611
H	26.6203277	22.3493932	25.5359122
H	25.0402079	20.6035011	26.3514711
C	32.6195730	27.4156418	27.7842370
C	33.7903024	26.9011185	28.3269289
N	34.1914527	25.6377118	28.0627626
C	32.3198810	25.3402876	26.6256770
C	33.4704355	24.8621621	27.2220133
H	32.3197093	28.4185220	28.0883023
H	34.4300292	27.4829349	28.9892048
H	31.7847186	24.6832261	25.9456070
H	33.8432635	23.8518339	27.0443366
C	29.5774167	33.3664241	23.0435589
C	30.1429423	34.6325788	22.9565442
N	30.5584546	35.2910309	24.0616465
C	29.8237313	33.4703560	25.4235156
C	30.3842941	34.7330686	25.2845717
H	29.2792298	32.8710424	22.1187884
H	30.2866237	35.1445708	22.0031032
H	29.6889543	33.0749978	26.4312790
H	30.7079168	35.3265252	26.1437491
O	28.5716815	27.4038242	22.8644302
O	26.7135179	28.6400482	26.5013871
H	25.8485439	29.0767851	26.4811565
C	31.2564267	36.5982779	23.9290002
H	31.0065044	37.0467512	22.9596811
H	30.9323865	37.2693487	24.7332717
H	32.3404738	36.4291825	24.0002830

C	21.2029125	30.2625377	18.4726579
H	20.2031520	30.4021564	18.9000220
H	21.5297772	31.1876807	17.9826649
H	21.1872354	29.4371014	17.7452775
C	22.6090106	20.9238261	27.2823019
H	21.8246463	20.8860659	26.5147870
H	23.1258880	19.9611301	27.3461747
H	22.1629376	21.1639846	28.2542320
C	35.4234891	25.0940241	28.6775168
H	35.2511560	24.0467382	28.9535829
H	36.2532733	25.1736088	27.9599170
H	35.6567672	25.6732015	29.5777381
H	27.2242478	29.1130608	27.1747295
Fe	27.7649752	28.0223409	24.4464805
C	30.3827349	26.6883647	20.1753191
C	30.6005171	25.4278741	19.5025189
C	31.8837881	24.8879299	19.5274566
C	32.9261922	25.5592859	20.1797491
C	32.7318617	26.8041474	20.8183576
C	31.4732675	27.3606996	20.8236976
H	32.0836090	23.9388652	19.0245284
H	33.9185131	25.1045130	20.1973981
H	33.5725159	27.3090682	21.2957264
H	31.2767564	28.3098898	21.3271274
C	29.4490144	24.7253824	18.8370088
H	29.0353133	23.9900913	19.5528649
H	29.8141635	24.1314829	17.9858533
C	29.1091086	27.2482911	20.2126004
H	28.9743006	28.1886141	20.7485275
H	29.0748831	26.5882607	23.0003217
C	28.3263399	25.6726799	18.4155211
H	27.4529247	25.0852870	18.0986957
H	28.6430731	26.2730279	17.5471286
C	27.9401113	26.6213666	19.5581863
H	27.2247192	27.3980859	19.2411856
H	27.4150464	26.0759484	20.3739224

^{6}PS(R)

N	27.1898686	29.7150316	23.7023332
N	25.9197602	27.1560396	24.1951242
N	28.2940780	26.2719816	25.6768656
N	29.4698826	28.9214508	25.3328001
C	26.0508951	29.8826208	22.9431949
C	27.7979930	30.9508709	23.8133951
C	27.4542252	25.2203943	25.9908487
C	29.4935693	26.0454645	26.3171450
C	25.0459284	27.6009441	23.2278404
C	25.4809837	25.9225655	24.6113428
C	29.8464450	30.2250139	25.0691588
C	30.5459795	28.2982135	25.9350128
C	25.9230162	31.2732887	22.5880745
C	26.9815574	31.9344406	23.1480420
C	28.1618991	24.2868435	26.8313687
C	29.4235000	24.7790494	27.0099793
C	24.0517673	26.5804187	22.9844563
C	24.2982068	25.5651592	23.8634606
C	31.2105924	30.4161718	25.5014776
C	31.6429717	29.2334251	26.0298360
C	25.1021495	28.8769669	22.6203857
C	29.0513229	31.2041217	24.4236269
C	30.5727024	26.9618257	26.4047105
C	26.1221751	25.0676045	25.5434412

H	25.1040996	31.7163338	22.0284618
H	27.1611609	33.0042953	23.1195432
H	27.7506106	23.3829201	27.2719860
H	30.2113092	24.3280231	27.6042788
H	23.2561664	26.6051687	22.2465521
H	23.7474980	24.6322670	23.9455799
H	31.7934741	31.3266561	25.4017836
H	32.6288417	29.0328843	26.4416937
C	24.1114066	29.1964455	21.5490122
C	29.6035862	32.6007728	24.3114344
C	31.7925599	26.4927300	27.1466774
C	25.2827948	23.9360151	26.0619653
C	24.5668607	29.6206262	20.2828103
C	23.6652982	29.9039271	19.2741673
N	22.3329054	29.7786611	19.4724083
C	22.7206344	29.0739793	21.7186942
C	21.8586573	29.3593104	20.6668158
H	25.6314274	29.7213488	20.0685045
H	23.9855198	30.2212158	18.2827865
H	22.2834386	28.7761488	22.6709255
H	20.7765187	29.2583880	20.7593109
C	24.0741289	24.2328227	26.7258626
C	23.2227436	23.2199392	27.1321722
N	23.5338771	21.9227364	26.9083457
C	25.5942169	22.5759839	25.8929129
C	24.7086823	21.5952696	26.3276619
H	23.7885717	25.2632735	26.9378480
H	22.2716217	23.4161127	27.6332028
H	26.5081380	22.2585111	25.3904529
H	24.9120240	20.5298393	26.2026274
C	32.1846517	27.0810944	28.3606191
C	33.2492907	26.5549276	29.0816696
N	33.9425525	25.4897231	28.6283720
C	32.5628671	25.4069358	26.6876311
C	33.6246017	24.9301875	27.4388665
H	31.6557141	27.9387168	28.7738376
H	33.5550023	26.9558879	30.0460157
H	32.3439491	24.9207700	25.7393248
H	34.2327212	24.0825082	27.1182780
C	29.9055248	33.1713808	23.0599728
C	30.4247716	34.4571725	22.9750660
N	30.6710027	35.1893437	24.0845478
C	29.8348888	33.4103565	25.4421755
C	30.3697181	34.6859887	25.3050110
H	29.7512174	32.6160599	22.1336783
H	30.6610751	34.9288519	22.0196552
H	29.5795908	33.0662904	26.4451494
H	30.5636002	35.3327937	26.1648328
O	28.6938346	27.1268610	22.9287061
O	26.7480325	28.5962633	26.5188500
H	25.8944409	29.0572443	26.4898501
C	31.3256763	36.5204721	23.9557680
H	31.0372813	36.9760115	22.9999248
H	31.0037592	37.1666864	24.7802766
H	32.4159871	36.3824465	23.9964694
C	21.4012156	30.0926021	18.3638418
H	20.4003649	30.2419652	18.7821140
H	21.7320242	31.0118888	17.8641738
H	21.3885159	29.2551638	17.6505950
C	22.5466260	20.8767569	27.2674646
H	21.7586216	20.8544648	26.5029865
H	23.0525056	19.9074625	27.3215180

H	22.1090521	21.1142246	28.2433066
C	35.0330520	24.9025419	29.4440278
H	34.6903847	23.9458962	29.8629921
H	35.9113449	24.7458554	28.8046724
H	35.2853967	25.5954830	30.2547863
H	27.2558061	29.0128030	27.2317333
Fe	27.7696963	27.9713713	24.6251907
C	30.1807294	26.9055941	21.0217003
C	30.3831526	25.9855705	19.9691720
C	31.6952722	25.5614662	19.7004340
C	32.7854879	26.0748764	20.3999799
C	32.5855709	27.0344285	21.3993141
C	31.2899489	27.4364891	21.7107409
H	31.8639509	24.8154902	18.9194517
H	33.7941328	25.7406588	20.1543687
H	33.4375569	27.4643412	21.9290092
H	31.1257935	28.1743826	22.5011556
C	29.2319381	25.4850559	19.1173459
H	28.9920531	24.4396617	19.3797752
H	29.5479399	25.4525172	18.0629695
C	28.7904328	27.3189403	21.4544493
H	28.6486389	28.4057294	21.3405195
H	29.1866395	26.3182046	23.1454778
C	27.9720198	26.3374674	19.2773088
H	27.1160500	25.8440257	18.7920734
H	28.1044058	27.3138904	18.7758860
C	27.6687542	26.5444755	20.7633379
H	26.7078412	27.0604069	20.9145185
H	27.5767578	25.5561008	21.2458685

^{6}TS1(S)

N	26.8300409	30.0097150	23.7625973
N	25.6628894	27.4239286	24.2994494
N	28.0673239	26.6578494	25.8244199
N	29.2519945	29.2251036	25.2218318
C	25.6813875	30.1349146	23.0378330
C	27.4141041	31.2478964	23.8483129
C	27.2235398	25.6541948	26.2406355
C	29.2664982	26.4890675	26.4654468
C	24.8274374	27.7839116	23.2661136
C	25.3023734	26.1662179	24.7046632
C	29.5999869	30.4934975	24.8298485
C	30.3918831	28.5925572	25.6620888
C	25.4757527	31.5350381	22.7212428
C	26.5288624	32.2247765	23.2476254
C	27.8980693	24.8517294	27.2372309
C	29.1597473	25.3545241	27.3679138
C	23.9409516	26.6770873	22.9693885
C	24.2082144	25.6973203	23.8813819
C	31.0295195	30.6523142	24.9707456
C	31.5240307	29.4755190	25.4533510
C	24.8107550	29.0681620	22.6712117
C	28.7253935	31.4891670	24.3207663
C	30.4127868	27.3225605	26.3036559
C	25.9319641	25.4019494	25.7229484
H	24.6115387	31.9524281	22.2124663
H	26.6710586	33.3012019	23.2372845
H	27.4648438	24.0188120	27.7835295
H	29.9247001	24.9965139	28.0517451
H	23.1934052	26.6290931	22.1841090
H	23.7281118	24.7247915	23.9499970
H	31.5983477	31.5362354	24.6978365

H	32.5696451	29.2531738	25.6395135
C	23.8605441	29.3439560	21.5557723
C	29.2911432	32.8746431	24.1858916
C	31.7084063	26.8246224	26.8562823
C	25.1425801	24.2365805	26.2423211
C	24.3498721	29.9362907	20.3709116
C	23.4938565	30.2350816	19.3295944
N	22.1740817	29.9495191	19.4041739
C	22.4832947	29.0499555	21.5933561
C	21.6695317	29.3508280	20.5080203
H	25.4083972	30.1694831	20.2535385
H	23.8407225	30.7100240	18.4135909
H	22.0145867	28.6164840	22.4753049
H	20.6002085	29.1297852	20.5051761
C	23.8646430	24.4576386	26.7985235
C	23.0726511	23.3943287	27.1950477
N	23.5064761	22.1188977	27.0658759
C	25.5787922	22.9006653	26.1753685
C	24.7465888	21.8673291	26.5925246
H	23.4739002	25.4665302	26.9314853
H	22.0722741	23.5307231	27.6125376
H	26.5555354	22.6400774	25.7658672
H	25.0471070	20.8187843	26.5403711
C	32.5962396	27.6355751	27.5953549
C	33.8096463	27.1327892	28.0438096
N	34.1592915	25.8458297	27.8149223
C	32.1133169	25.4882201	26.6476604
C	33.3240488	25.0292684	27.1353272
H	32.3471572	28.6626332	27.8554257
H	34.5251875	27.7445841	28.5963262
H	31.4839325	24.7884435	26.1011318
H	33.6575308	24.0005572	26.9930687
C	29.4342201	33.5040160	22.9351128
C	30.0259074	34.7577777	22.8370062
N	30.4815924	35.4043303	23.9328974
C	29.7373848	33.6007050	25.3110539
C	30.3231125	34.8502937	25.1597692
H	29.1084127	33.0144645	22.0162801
H	30.1600956	35.2674559	21.8807044
H	29.6217736	33.2020392	26.3203705
H	30.6810672	35.4347045	26.0113702
O	28.1823056	27.6594198	23.0864327
O	26.6825252	29.0946743	26.4392877
H	25.7163553	29.1157991	26.5188092
C	31.2121161	36.6913430	23.7824791
H	30.9371842	37.1545601	22.8270103
H	30.9412443	37.3648276	24.6043477
H	32.2924109	36.4877806	23.8068720
C	21.2954632	30.2817798	18.2601402
H	20.2849112	30.4752466	18.6365188
H	21.6793348	31.1804403	17.7621154
H	21.2800573	29.4361965	17.5565659
C	22.5804241	21.0136703	27.4102000
H	21.7798781	20.9750708	26.6598514
H	23.1337796	20.0690972	27.4249460
H	22.1493762	21.2003147	28.4003530
C	35.4651914	25.3372748	28.2939511
H	35.4209392	24.2447653	28.3645057
H	36.2548435	25.6440737	27.5920933
H	35.6672104	25.7592636	29.2857738
H	27.0118696	28.7784356	27.2938042
Fe	27.5679160	28.2348087	24.5302445

C	30.2483139	25.9723885	20.9668861
C	30.6697685	25.3586926	19.7207235
C	32.0370587	25.2842319	19.4630570
C	32.9580428	25.9572569	20.2757722
C	32.5311899	26.6904075	21.4205600
C	31.2042223	26.6774679	21.7716663
H	32.3955262	24.7160255	18.6016723
H	34.0193503	25.9359895	20.0205680
H	33.2675709	27.2337412	22.0167279
H	30.8431220	27.1813433	22.6715576
C	29.6479670	24.7414755	18.8079265
H	29.6631438	23.6442511	18.9570088
H	29.9604930	24.8819267	17.7608377
C	28.8948470	25.7671433	21.4546727
H	28.5279140	26.8111699	21.9858541
H	28.9203407	25.3122268	22.4615350
C	28.2388509	25.2883404	19.0510065
H	27.5153014	24.7518791	18.4194546
H	28.1996781	26.3513276	18.7516965
C	27.8589674	25.1479526	20.5269011
H	26.8713279	25.5963473	20.7291905
H	27.7436397	24.0775126	20.7812901

[6]IM(S)

N	26.8645478	30.0699276	23.8762887
N	25.7161099	27.4460322	24.3605953
N	28.1117588	26.6830351	25.8875554
N	29.2381867	29.3357913	25.4399102
C	25.7117711	30.1859136	23.1473158
C	27.4188703	31.3218931	23.9908686
C	27.2715012	25.6736887	26.2940795
C	29.3004543	26.5311208	26.5469842
C	24.8838638	27.8157262	23.3319592
C	25.3517527	26.1905274	24.7608957
C	29.5867655	30.6031070	25.0419752
C	30.3857550	28.6960217	25.8509064
C	25.4864795	31.5843243	22.8480609
C	26.5247745	32.2875642	23.3910119
C	27.9491632	24.8644698	27.2810325
C	29.2044552	25.3793155	27.4276602
C	24.0053115	26.7055617	23.0165877
C	24.2664345	25.7211972	23.9253735
C	31.0170655	30.7639877	25.1780574
C	31.5149528	29.5835656	25.6465485
C	24.8569448	29.1121471	22.7571987
C	28.7181732	31.5852231	24.4945545
C	30.4300067	27.3949206	26.4267349
C	25.9823196	25.4200491	25.7748697
H	24.6216671	31.9948626	22.3350441
H	26.6463859	33.3670024	23.3911954
H	27.5195400	24.0199474	27.8113095
H	29.9684725	25.0228008	28.1134552
H	23.2697771	26.6556149	22.2203238
H	23.7907676	24.7454241	23.9804846
H	31.5853034	31.6478214	24.9045982
H	32.5632017	29.3601578	25.8165598
C	23.8995398	29.3917089	21.6500642
C	29.2845720	32.9650406	24.3298074
C	31.7333832	26.8814313	26.9382305
C	25.1922461	24.2544591	26.2883443
C	24.3600935	30.0308543	20.4772521
C	23.4920310	30.3154593	19.4414897

N	22.1860544	29.9702444	19.5071806
C	22.5324606	29.0486951	21.6809396
C	21.7071050	29.3338686	20.6016049
H	25.4072160	30.3115246	20.3631478
H	23.8181340	30.8248135	18.5358398
H	22.0804061	28.5845869	22.5550442
H	20.6477798	29.0690920	20.5949781
C	23.9149516	24.4768826	26.8457653
C	23.1177094	23.4137920	27.2315717
N	23.5454834	22.1374180	27.0902070
C	25.6244474	22.9174630	26.2139278
C	24.7867123	21.8845003	26.6197290
H	23.5323020	25.4874749	26.9889681
H	22.1173681	23.5504523	27.6490265
H	26.6018814	22.6578784	25.8050287
H	25.0822149	20.8349963	26.5593083
C	32.6344403	27.6569134	27.6995874
C	33.8489105	27.1278230	28.1134397
N	34.1876519	25.8489985	27.8280815
C	32.1326757	25.5550419	26.6602626
C	33.3433963	25.0682797	27.1176329
H	32.3947564	28.6743815	28.0045134
H	34.5740513	27.7102820	28.6845002
H	31.4960501	24.8884527	26.0810124
H	33.6711237	24.0458691	26.9259092
C	29.4137335	33.5706583	23.0646904
C	30.0184018	34.8141200	22.9303312
N	30.4995281	35.4778762	24.0052832
C	29.7595030	33.7099163	25.4315716
C	30.3561864	34.9491547	25.2453776
H	29.0697693	33.0665272	22.1605869
H	30.1441578	35.3007702	21.9607682
H	29.6585454	33.3330388	26.4506655
H	30.7348053	35.5459541	26.0791806
O	28.4015829	27.7756736	23.0191953
O	26.5587599	28.9616008	26.6606695
H	25.6625261	29.3293325	26.6771437
C	31.2373965	36.7554356	23.8190812
H	30.9775186	37.1847172	22.8438905
H	30.9573594	37.4602057	24.6112954
H	32.3163010	36.5493060	23.8663737
C	21.2930140	30.2737579	18.3670041
H	20.2843983	30.4655385	18.7506598
H	21.6646739	31.1643650	17.8461152
H	21.2765109	29.4143935	17.6804147
C	22.6120178	21.0331942	27.4159000
H	21.8201215	21.0021772	26.6559108
H	23.1622255	20.0867354	27.4304224
H	22.1700372	21.2134795	28.4024521
C	35.4912516	25.3095111	28.2767221
H	35.4307218	24.2163738	28.3200825
H	36.2778615	25.6232052	27.5745403
H	35.7110126	25.7000941	29.2772437
H	27.0509282	29.4442641	27.3406672
Fe	27.6077460	28.2724027	24.6610306
C	30.2622405	25.8925445	20.4335131
C	30.9109513	25.0897992	19.4201841
C	32.3005772	25.0770046	19.3937480
C	33.0387779	25.8970364	20.2569736
C	32.4150604	26.7289693	21.2148747
C	31.0429723	26.7092362	21.3260969
H	32.8162928	24.4491259	18.6657687

H	34.1285560	25.8942745	20.1858756
H	33.0258773	27.3558698	21.8673651
H	30.5074339	27.2822305	22.0883249
C	30.0905797	24.2525649	18.4802335
H	30.1357842	23.2087669	18.8406229
H	30.5751225	24.2175188	17.4939484
C	28.8877703	25.8071252	20.6086306
H	28.4390344	28.5389568	22.4231218
H	28.4559453	26.3475915	21.4671830
C	28.6396504	24.7238639	18.3575328
H	28.0413964	23.9778623	17.8200717
H	28.6063014	25.6493665	17.7585008
C	28.0143517	25.0061401	19.7267882
H	27.0209771	25.4819774	19.6504118
H	27.8070070	24.0610678	20.2807093

[6]TS2(S)

N	26.8625044	30.0052714	23.7251366
N	25.7290048	27.3648169	24.1710866
N	28.1577364	26.5577935	25.6133997
N	29.2675752	29.2335049	25.1971665
C	25.6924825	30.1361311	23.0284023
C	27.4197961	31.2546296	23.8499123
C	27.3175322	25.5507009	26.0334349
C	29.3424369	26.4197069	26.2893563
C	24.8547168	27.7682506	23.1903021
C	25.3550254	26.1126099	24.5735797
C	29.6121165	30.5103772	24.8219177
C	30.4165075	28.5946546	25.6064675
C	25.4579217	31.5399385	22.7643159
C	26.5086046	32.2325231	23.2966482
C	27.9983489	24.7473449	27.0237658
C	29.2468509	25.2744094	27.1774041
C	23.9363169	26.6834074	22.9046572
C	24.2192229	25.6812029	23.7869575
C	31.0423471	30.6695949	24.9493238
C	31.5430313	29.4847408	25.4062127
C	24.8242728	29.0736389	22.6399195
C	28.7325041	31.5072189	24.3215982
C	30.4637628	27.2956176	26.1896832
C	26.0094136	25.3161273	25.5522386
H	24.5783160	31.9577778	22.2832888
H	26.6286911	33.3116334	23.3218850
H	27.5753688	23.9077738	27.5693438
H	30.0038101	24.9315966	27.8768085
H	23.1569862	26.6641774	22.1502096
H	23.7226056	24.7172290	23.8558493
H	31.6094459	31.5561431	24.6808083
H	32.5915841	29.2584345	25.5709109
C	23.8525313	29.3762471	21.5506324
C	29.2915679	32.8940349	24.1883895
C	31.7576634	26.8194784	26.7610589
C	25.2100125	24.1680532	26.0914844
C	24.3175586	29.9968866	20.3702324
C	23.4463022	30.2954904	19.3415942
N	22.1331000	29.9832386	19.4240022
C	22.4780837	29.0702358	21.6022263
C	21.6493355	29.3679797	20.5279408
H	25.3704447	30.2500169	20.2445052
H	23.7753806	30.7888493	18.4286395
H	22.0246600	28.6258173	22.4865864
H	20.5843363	29.1284921	20.5318536

C	23.9436706	24.4196283	26.6625476
C	23.1326189	23.3784830	27.0767934
N	23.5361236	22.0928323	26.9525140
C	25.6167329	22.8223442	26.0344718
C	24.7664066	21.8112957	26.4704523
H	23.5818093	25.4382442	26.7976166
H	22.1413336	23.5399876	27.5072355
H	26.5831690	22.5360977	25.6177252
H	25.0439586	20.7561722	26.4255228
C	32.6110683	27.6379660	27.5321133
C	33.8221243	27.1528958	28.0049496
N	34.2014055	25.8750706	27.7726963
C	32.1961037	25.4946106	26.5447290
C	33.3998685	25.0510310	27.0617216
H	32.3371832	28.6568176	27.7997394
H	34.5117597	27.7719625	28.5813501
H	31.5944461	24.7924010	25.9713396
H	33.7541784	24.0292254	26.9182313
C	29.3829948	33.5441396	22.9425817
C	29.9779699	34.7954479	22.8355746
N	30.4869899	35.4232819	23.9187970
C	29.7932437	33.6032342	25.3020783
C	30.3793665	34.8512669	25.1430550
H	29.0162440	33.0704654	22.0310301
H	30.0741461	35.3172374	21.8811509
H	29.7222732	33.1926778	26.3104661
H	30.7778550	35.4212063	25.9863885
O	28.3540788	27.7071756	22.7533363
O	26.6277872	28.8226169	26.4637018
H	25.7572730	29.2488504	26.4608591
C	31.2178731	36.7086161	23.7572703
H	30.9377513	37.1669039	22.8010593
H	30.9523531	37.3872635	24.5769091
H	32.2980100	36.5040116	23.7759014
C	21.2388127	30.3022493	18.2886874
H	20.2286154	30.4793327	18.6742916
H	21.6046951	31.2061629	17.7869618
H	21.2306023	29.4564518	17.5852392
C	22.5876028	21.0121831	27.3127636
H	21.7958726	20.9672802	26.5533353
H	23.1251419	20.0596012	27.3583014
H	22.1479926	21.2309593	28.2927536
C	35.5037034	25.3855555	28.2792721
H	35.4633258	24.2953493	28.3806200
H	36.2996444	25.6752174	27.5771494
H	35.6938743	25.8367852	29.2603436
H	27.1578232	29.3268051	27.0986443
Fe	27.6276682	28.1845344	24.4154084
C	30.3391484	25.9468452	20.8855004
C	30.7698431	25.3531151	19.6421251
C	32.1382221	25.3432842	19.3716431
C	33.0331044	26.0359120	20.1920451
C	32.6006117	26.7238835	21.3493258
C	31.2765126	26.6446563	21.7177452
H	32.5112150	24.8064218	18.4970346
H	34.0927039	26.0520305	19.9311062
H	33.3217639	27.2820077	21.9500029
H	30.8978838	27.1202873	22.6250730
C	29.7746468	24.6774757	18.7354047
H	29.8600445	23.5837093	18.8735056
H	30.0636980	24.8430794	17.6862927
C	29.0573786	25.6415680	21.3451092

H	28.6118000	28.3767118	22.1058290
H	28.8455353	25.7616208	22.4039714
C	28.3400176	25.1489289	18.9874977
H	27.6298257	24.5435090	18.4065874
H	28.2344798	26.1907552	18.6375926
C	28.0002712	25.0904754	20.4793699
H	27.0578164	25.6125027	20.7288102
H	27.8144463	24.0488512	20.8235969

[6]PS(S)

N	26.9373178	29.8860594	23.6621262
N	25.7584934	27.2827889	24.2070418
N	28.1988419	26.4832115	25.6167806
N	29.3112440	29.1300483	25.1542068
C	25.7640586	30.0115092	22.9548705
C	27.4939724	31.1434897	23.7685765
C	27.3645256	25.4634720	26.0406416
C	29.4048516	26.3247347	26.2662865
C	24.8666561	27.6824341	23.2362794
C	25.3633760	26.0442078	24.6488958
C	29.6583098	30.4162810	24.7909104
C	30.4641066	28.4980079	25.5844911
C	25.5495027	31.4040959	22.6451261
C	26.5981190	32.1049700	23.1760705
C	28.0688120	24.6410222	26.9899219
C	29.3253407	25.1619393	27.1230734
C	23.9099862	26.6217824	23.0138298
C	24.1927606	25.6321899	23.9120714
C	31.0802606	30.5810406	24.9475794
C	31.5820183	29.3968351	25.4073211
C	24.8626709	28.9613729	22.6307528
C	28.7916942	31.4095612	24.2679905
C	30.5206166	27.2026333	26.1674523
C	26.0415559	25.2409677	25.6004403
H	24.6808807	31.8157955	22.1390441
H	26.7248029	33.1831980	23.1740749
H	27.6571899	23.7897117	27.5263282
H	30.0997302	24.7970213	27.7912705
H	23.1127003	26.6039837	22.2780545
H	23.6740666	24.6831320	24.0149101
H	31.6444098	31.4713895	24.6878264
H	32.6293250	29.1746905	25.5837629
C	23.8526537	29.2452080	21.5699299
C	29.3437380	32.8024305	24.1463839
C	31.8201299	26.7359101	26.7434811
C	25.2559377	24.0835505	26.1445999
C	24.2798334	29.7430237	20.3203284
C	23.3613883	30.0074538	19.3224538
N	22.0420486	29.7762093	19.5100471
C	22.4732109	29.0255870	21.7324499
C	21.5971696	29.2787082	20.6845536
H	25.3353155	29.9230459	20.1133819
H	23.6552133	30.4053715	18.3522235
H	22.0536874	28.6809440	22.6768349
H	20.5265695	29.0907232	20.7631396
C	24.0347192	24.3320208	26.8039070
C	23.2268107	23.2837504	27.2093604
N	23.5912473	22.0008611	26.9851228
C	25.6326148	22.7377871	25.9899091
C	24.7851995	21.7207933	26.4183643
H	23.7018463	25.3501418	27.0068009
H	22.2665895	23.4383438	27.7059442

H	26.5664051	22.4588196	25.4997987
H	25.0334369	20.6644482	26.2962520
C	32.6404652	27.5489536	27.5524445
C	33.8528271	27.0720230	28.0335439
N	34.2607667	25.8107557	27.7675897
C	32.2870795	25.4295640	26.4908504
C	33.4916139	24.9941450	27.0150584
H	32.3436810	28.5553439	27.8428843
H	34.5198352	27.6852721	28.6416311
H	31.7110505	24.7348913	25.8835450
H	33.8714970	23.9859836	26.8426821
C	29.4655069	33.4506907	22.9027007
C	30.0434599	34.7121211	22.8139573
N	30.5099934	35.3478550	23.9112455
C	29.7994748	33.5189179	25.2742905
C	30.3737789	34.7753091	25.1319522
H	29.1368870	32.9714906	21.9794362
H	30.1594656	35.2356682	21.8630136
H	29.7021081	33.1093475	26.2809878
H	30.7390091	35.3506171	25.9867621
O	28.3316115	27.5737171	22.6504016
O	26.6333678	28.7721485	26.4680842
H	25.7711260	29.2181737	26.4665230
C	31.2288719	36.6436640	23.7673193
H	30.9351472	37.1185305	22.8230995
H	30.9670624	37.3022646	24.6040615
H	32.3109408	36.4475615	23.7706618
C	21.0913197	30.0717347	18.4142769
H	20.0842804	30.1505567	18.8371329
H	21.3729411	31.0231281	17.9471875
H	21.1270624	29.2613134	17.6713416

C	22.6335539	20.9189152	27.3181175
H	21.8663714	20.8746113	26.5332752
H	23.1710797	19.9670849	27.3792900
H	22.1631030	21.1374518	28.2834969
C	35.5640448	25.3286710	28.2808300
H	35.5251428	24.2392711	28.3903237
H	36.3586295	25.6153668	27.5757130
H	35.7500947	25.7881067	29.2585315
H	27.1550239	29.1993078	27.1646632
Fe	27.5979945	28.1382615	24.5514229
C	30.0072292	26.4732718	21.2286913
C	30.0101879	26.0084166	19.8946174
C	31.2121679	26.0712709	19.1700964
C	32.3624979	26.6446315	19.7097769
C	32.3357923	27.1705679	21.0083769
C	31.1703740	27.0680336	21.7619654
H	31.2384829	25.6589289	18.1575359
H	33.2772728	26.6953607	19.1149398
H	33.2308678	27.6340138	21.4291724
H	31.1623537	27.4270248	22.7958923
C	28.7649478	25.4379304	19.2452878
H	28.8501196	24.3372504	19.1874825
H	28.7121139	25.7792198	18.1989135
C	28.8149902	26.2555724	22.1300629
H	28.5710236	28.2734436	22.0212908
H	29.1373477	25.7598846	23.0538758
C	27.4905912	25.8069373	20.0051544
H	26.6254230	25.2657064	19.5918872
H	27.2764458	26.8859486	19.8837269
C	27.6636446	25.4792065	21.4890459
H	26.7420226	25.6606546	22.0621768

H	27.8860433	24.4037407	21.5943961

For Modified Cage: '*mod1*'

$^2RS_{mod1}$

N	21.8779140	25.6408914	24.2087202
N	20.8394425	23.0462256	24.6788871
N	23.2007557	22.3931033	26.1304390
N	24.2962067	24.9233937	25.5215023
C	20.7188080	25.7485000	23.4923576
C	22.4058615	26.9075831	24.3016786
C	22.4459530	21.3014351	26.5003039
C	24.4487839	22.1922714	26.6660498
C	19.9731317	23.3881592	23.6675842
C	20.5131282	21.7605358	25.0406063
C	24.6106903	26.2153709	25.1959269
C	25.4713313	24.3112215	25.8910865
C	20.4466294	27.1422026	23.2156357
C	21.4780839	27.8621035	23.7420295
C	23.2094500	20.4442858	27.3747136
C	24.4509330	20.9899464	27.4727900
C	19.1211397	22.2639514	23.3454999
C	19.4243507	21.2720901	24.2251210
C	26.0369178	26.4123172	25.3021391
C	26.5744700	25.2263153	25.7023303
C	19.8894513	24.6707193	23.0946821
C	23.7083141	27.2031268	24.7439932
C	25.5646200	23.0318188	26.4747896
C	21.1790176	20.9723728	25.9933297
H	19.5583224	27.5335805	22.7283253
H	21.5806117	28.9439092	23.7547655
H	22.8441930	19.5347630	27.8396822
H	25.2838599	20.6101425	28.0569676
H	18.3938553	22.2070127	22.5427565
H	18.9927937	20.2748291	24.2708571
H	26.5734734	27.3245698	25.0589686
H	27.6273639	25.0098853	25.8510430
C	18.9096766	24.9403807	22.0167705
C	24.2424034	28.5880490	24.6066635
C	26.8865881	22.5712683	26.9885612
C	20.5542551	19.6568511	26.3469037
C	19.3283209	25.6335055	20.8567153
C	18.4201203	25.9831616	19.8807361
N	17.1108535	25.6578221	19.9955967
C	17.5505488	24.5674900	22.0689021
C	16.6795045	24.9347626	21.0563627
H	20.3740774	25.9076798	20.7188105
H	18.7073921	26.5379612	18.9878298
H	17.1360411	24.0346560	22.9216840
H	15.6173423	24.6835277	21.0876124
C	19.3162128	19.5992029	27.0202497
C	18.6891798	18.3812610	27.1964472
N	19.2433399	17.2358726	26.7347846
C	21.1292631	18.4495469	25.9442817
C	20.4545321	17.2482334	26.1535495
H	18.8247247	20.5009841	27.3867719
H	17.7204475	18.2849721	27.6871001
H	22.0720935	18.4080525	25.4011172
H	20.8497482	16.2974863	25.7896598
C	27.6422982	23.3919841	27.8498238
C	28.8639926	22.9617149	28.3314186
N	29.3409540	21.7387074	28.0101710
C	27.4248177	21.3092565	26.6605195

C	28.6446840	20.9154911	27.1938900
H	27.2744197	24.3628128	28.1756386
H	29.4827606	23.5729621	28.9881460
H	26.9186372	20.6043099	25.9964645
H	29.0830824	19.9394661	26.9770837
C	24.3053817	29.2507875	23.3636572
C	24.9115289	30.4933389	23.2511013
N	25.4519608	31.1115613	24.3273899
C	24.7758503	29.2883513	25.7137380
C	25.3665059	30.5303086	25.5510313
H	23.9169400	28.7794183	22.4599561
H	24.9980263	31.0163891	22.2971477
H	24.7206263	28.8642330	26.7171365
H	25.7875696	31.0919927	26.3883712
O	23.2514730	23.4171809	23.6184652
O	21.8019366	24.7410123	26.8643623
H	20.8317615	24.7276092	26.8737225
C	26.1864356	32.3896767	24.1629916
H	25.9127154	32.8443050	23.2034390
H	25.9164830	33.0751900	24.9759497
H	27.2674320	32.1910444	24.1872083
C	16.1654056	26.0939919	18.9525183
H	15.1703660	26.1922329	19.4008202
H	16.4892442	27.0649520	18.5594233
H	16.1433017	25.3534692	18.1402418
C	18.4563692	15.9841214	26.7888481
H	17.4887854	16.1733804	26.3078537
H	18.9835085	15.2206813	26.2090053
H	18.3328937	15.6792821	27.8352506
C	30.6388433	21.3042402	28.5589726
H	30.5637793	20.2555833	28.8594292
H	31.4241618	21.4271321	27.7990281
H	30.8627538	21.9183873	29.4360243
H	22.0723234	24.1923953	27.6169057
Fe	22.6199208	23.9494686	25.0000864
C	25.9089734	21.6560195	21.2371415
C	25.6016099	20.5873874	20.2788033
C	26.1578562	19.3162151	20.4728864
C	26.9840618	19.0638568	21.5546671
C	27.3073760	20.1207489	22.4860542
C	26.7762991	21.3873961	22.3085207
H	25.9099847	18.5084375	19.7841629
H	27.3967958	18.0671080	21.7272680
H	27.9599470	19.8801745	23.3293751
H	27.0088427	22.1875061	23.0150329
C	24.6698207	20.8347494	19.1341412
H	23.7322657	20.2952561	19.3828874
H	25.0550413	20.3027345	18.2472577
C	25.3270720	23.0236236	21.0576729
H	26.1460451	23.6619249	20.6621090
H	25.0821109	23.4371429	22.0509306
C	24.3530181	22.3057458	18.8378897
H	23.4756230	22.3590351	18.1761245
H	25.1804163	22.7690709	18.2758718
C	24.1126949	23.0921963	20.1259634
H	23.8933745	24.1475653	19.8981379
H	23.2343794	22.7001495	20.6638567

^{2}TS1-R$_{mod1}$

N	21.9760964	25.5500237	24.0200606
N	20.8772582	23.0022862	24.5952440
N	23.2802665	22.3268801	25.9725007

N	24.4128260	24.7911195	25.2274809
C	20.7842716	25.6755421	23.3585293
C	22.5192316	26.8140615	24.1002860
C	22.5148152	21.2524474	26.3786273
C	24.5409609	22.1089774	26.4794112
C	19.9787325	23.3475140	23.6108780
C	20.5362559	21.7286381	24.9904706
C	24.7361272	26.0885850	24.9122541
C	25.5843026	24.1784614	25.6204451
C	20.5187170	27.0719281	23.1004487
C	21.5785090	27.7785550	23.5881696
C	23.2875129	20.3962310	27.2428087
C	24.5406370	20.9215574	27.3029343
C	19.0826596	22.2432161	23.3505820
C	19.3983859	21.2583994	24.2332729
C	26.1599562	26.2747927	25.0243839
C	26.6900331	25.0869511	25.4338646
C	19.9115316	24.6160159	23.0085761
C	23.8364301	27.0940287	24.5004686
C	25.6660329	22.9209799	26.2489089
C	21.2259078	20.9357143	25.9206296
H	19.6148956	27.4758408	22.6537261
H	21.6913112	28.8589548	23.6034179
H	22.9193160	19.4983072	27.7270136
H	25.3841391	20.5358262	27.8671331
H	18.3208187	22.1931548	22.5804135
H	18.9444950	20.2731002	24.3118749
H	26.6993512	27.1869212	24.7879062
H	27.7399460	24.8655115	25.5966177
C	18.9045378	24.9015760	21.9587370
C	24.3636921	28.4865933	24.4008844
C	26.9819271	22.4772929	26.7992841
C	20.5998189	19.6270295	26.2994125
C	19.3078109	25.5841947	20.7870388
C	18.3810014	25.9630558	19.8400978
N	17.0685148	25.6678412	19.9906251
C	17.5394921	24.5618505	22.0490159
C	16.6504918	24.9488184	21.0585028
H	20.3555630	25.8315706	20.6181948
H	18.6552637	26.5181165	18.9433139
H	17.1335249	24.0392591	22.9121082
H	15.5858006	24.7144282	21.1141205
C	19.3805589	19.5822981	27.0075590
C	18.7390528	18.3723393	27.1877533
N	19.2629990	17.2235139	26.6991986
C	21.1503278	18.4147342	25.8784877
C	20.4630299	17.2215180	26.0958461
H	18.9139255	20.4876641	27.3972539
H	17.7812529	18.2855258	27.7015259
H	22.0792369	18.3580679	25.3129824
H	20.8381231	16.2682354	25.7168985
C	27.6869956	23.3173418	27.6840721
C	28.8981682	22.9151699	28.2134985
N	29.4092830	21.7015627	27.9119085
C	27.5507155	21.2250939	26.4936596
C	28.7605175	20.8627982	27.0743369
H	27.2876071	24.2815839	27.9909520
H	29.4793329	23.5406316	28.8908780
H	27.0877244	20.5031340	25.8144894
H	29.2297353	19.8976959	26.8781443
C	24.3918058	29.2001831	23.1860054
C	24.9690076	30.4606835	23.1162390

N	25.5176717	31.0433861	24.2070751
C	24.9048677	29.1498392	25.5261147
C	25.4695783	30.4086674	25.4054469
H	23.9934915	28.7617980	22.2701280
H	25.0228298	31.0278588	22.1855187
H	24.8731015	28.6860771	26.5125432
H	25.8966762	30.9421970	26.2579282
O	23.1843923	23.1832498	23.3859791
O	21.9663574	24.6931499	26.6616041
H	20.9952833	24.6927719	26.6758452
C	26.2206708	32.3448671	24.0858088
H	25.9240230	32.8311680	23.1489045
H	25.9464741	32.9892761	24.9305419
H	27.3058541	32.1696690	24.0881138
C	16.1077749	26.1336470	18.9738277
H	15.1151360	26.1988993	19.4330485
H	16.4158729	27.1243264	18.6180393
H	16.0923027	25.4258234	18.1329572
C	18.4524824	15.9863509	26.7459918
H	17.4893301	16.1969289	26.2646523
H	18.9657147	15.2164298	26.1621517
H	18.3211036	15.6790184	27.7906091
C	30.6966777	21.2919373	28.5033743
H	30.6264237	20.2459966	28.8148744
H	31.4995554	21.4145296	27.7621207
H	30.8886562	21.9208467	29.3775831
H	22.2340266	24.1229802	27.3999402
Fe	22.7082860	23.8540591	24.8080995
C	25.7312367	22.1483410	21.4782213
C	25.4064103	21.2304542	20.4068158
C	26.0018306	19.9756333	20.4023001
C	26.8655227	19.5781554	21.4367762
C	27.2332670	20.4889394	22.4677129
C	26.6914024	21.7514216	22.4692966
H	25.7728523	19.2725920	19.5980438
H	27.2592999	18.5604515	21.4735889
H	27.9175325	20.1423968	23.2458850
H	26.9453454	22.4679765	23.2551536
C	24.4656032	21.6540301	19.3064913
H	23.4639469	21.2414350	19.5243729
H	24.7762825	21.1736280	18.3660965
C	25.0606840	23.4212865	21.5946952
H	25.6897603	24.2037354	22.0516892
H	24.2716891	23.2721704	22.5900129
C	24.3771514	23.1743521	19.1397742
H	23.5772005	23.4246610	18.4246846
H	25.3115387	23.5556557	18.6987596
C	24.1141457	23.8732659	20.4797639
H	24.1764416	24.9678540	20.3667194
H	23.0783261	23.6546283	20.7977524

^{2}TS1-S$_{mod1}$

N	22.0393443	25.6222572	24.2992363
N	20.9695631	23.0643131	24.8906180
N	23.4498871	22.3586453	26.1322911
N	24.5196750	24.8808034	25.4450937
C	20.8566675	25.7277264	23.6148093
C	22.5902488	26.8859535	24.3327906
C	22.7259004	21.2411012	26.4982574
C	24.7401663	22.1328900	26.5608684
C	20.0315811	23.4164493	23.9447137
C	20.6446132	21.7841668	25.2879109

C	24.8252282	26.1792379	25.1155530
C	25.7110205	24.2682007	25.7768156
C	20.6071709	27.1111042	23.2898753
C	21.6608988	27.8326872	23.7694930
C	23.5615512	20.3359750	27.2461659
C	24.8071359	20.8820483	27.2821604
C	19.1047149	22.3273550	23.7365226
C	19.4569025	21.3350856	24.5943110
C	26.2511509	26.3740272	25.1789327
C	26.8021104	25.1877964	25.5616683
C	19.9738947	24.6664167	23.3049308
C	23.9118117	27.1740184	24.7106441
C	25.8383995	22.9845360	26.3418637
C	21.4063566	20.9460909	26.1188800
H	19.7147684	27.4977275	22.8059864
H	21.7799836	28.9120713	23.7398601
H	23.2395906	19.3920580	27.6730820
H	25.6879007	20.4642665	27.7605886
H	18.3017871	22.2819818	23.0089675
H	18.9941095	20.3553845	24.6809928
H	26.7779472	27.2882204	24.9213345
H	27.8589509	24.9705992	25.6803764
C	18.9891555	24.9003154	22.2187025
C	24.4377713	28.5651671	24.5596990
C	27.1923210	22.5259741	26.7849812
C	20.8126694	19.6021314	26.4303106
C	19.4322124	25.4640313	20.9994460
C	18.5380470	25.7449542	19.9871868
N	17.2177604	25.4817594	20.1310296
C	17.6168145	24.5924081	22.3061478
C	16.7598563	24.8887221	21.2582191
H	20.4874788	25.6849573	20.8382135
H	18.8466679	26.1888581	19.0401164
H	17.1819722	24.1572086	23.2024148
H	15.6898567	24.6767679	21.3088140
C	19.6537989	19.4900705	27.2246329
C	18.9999300	18.2762499	27.3125641
N	19.4539497	17.1878877	26.6481829
C	21.3053215	18.4361886	25.8360175
C	20.6058352	17.2381286	25.9568871
H	19.2416753	20.3486621	27.7551511
H	18.0863579	18.1403395	27.8922508
H	22.1936866	18.4238510	25.2071403
H	20.9295540	16.3303732	25.4431930
C	27.9527934	23.3081336	27.6813232
C	29.2195342	22.9030977	28.0577049
N	29.7413996	21.7449954	27.5872308
C	27.7531971	21.3142368	26.3438171
C	29.0267134	20.9470773	26.7650822
H	27.5590523	24.2329443	28.1010635
H	29.8474701	23.4873224	28.7333898
H	27.2415841	20.6289369	25.6647848
H	29.4920427	20.0162655	26.4356451
C	24.4909767	29.2177393	23.3121294
C	25.0656553	30.4757305	23.1938540
N	25.5893433	31.1113370	24.2673419
C	24.9532497	29.2811057	25.6632289
C	25.5186255	30.5349608	25.4934370
H	24.1129212	28.7331293	22.4109253
H	25.1372554	30.9980349	22.2381764
H	24.9030691	28.8638220	26.6697668
H	25.9284797	31.1094472	26.3277086

O	23.2848878	23.3043012	23.6117618
O	22.1211951	24.7003259	26.9714873
H	21.1508388	24.7292004	27.0025670
C	26.2910187	32.4079974	24.0960837
H	25.9877215	32.8609544	23.1446446
H	26.0210639	33.0816651	24.9188448
H	27.3763085	32.2326032	24.0976034
C	16.2850459	25.8445491	19.0478978
H	15.2943052	26.0169482	19.4832105
H	16.6403722	26.7607498	18.5618207
H	16.2336150	25.0292706	18.3126962
C	18.6112607	15.9733304	26.6071854
H	17.6908716	16.2235350	26.0631907
H	19.1404062	15.2021591	26.0404393
H	18.4010268	15.6492042	27.6335955
C	31.1282891	21.3910002	27.9550224
H	31.3361392	20.3678815	27.6367839
H	31.8198999	22.0811485	27.4529743
H	31.2396959	21.4752503	29.0423346
H	22.3816412	24.0869668	27.6772740
Fe	22.8083956	23.9361050	25.0510068
C	25.0027389	21.7517508	21.3336571
C	25.6975164	21.3540232	20.1303560
C	26.5645293	20.2674486	20.1989233
C	26.7995843	19.5977050	21.4127075
C	26.1352839	20.0003886	22.6014674
C	25.2542164	21.0514102	22.5556959
H	27.0423102	19.9019990	19.2861590
H	27.4794777	18.7458522	21.4687274
H	26.3246700	19.4493303	23.5229910
H	24.7195333	21.3749022	23.4502517
C	25.3755225	22.0241280	18.8168571
H	25.4821886	21.2806885	18.0157535
H	26.1299037	22.8007113	18.6078086
C	23.9655072	22.7553782	21.2718102
H	23.8241012	23.3183186	22.4637837
H	22.9994194	22.3099290	21.5733828
C	24.0063142	22.7129165	18.7816992
H	23.1939443	21.9688768	18.7612233
H	23.9138454	23.3033598	17.8586565
C	23.8558612	23.6055984	20.0240518
H	24.6510133	24.3754374	20.0143348
H	22.8942670	24.1446518	20.0138503

For modified cage: *'mod2'*

$^{2}RS_{mod2}$

N	22.0329668	25.8554698	23.7080609
N	20.8188246	23.4557761	24.5334693
N	23.1833969	22.8188304	26.0207524
N	24.4563340	25.1393761	24.9796775
C	20.8181167	26.0105459	23.0987693
C	22.6835249	27.0589041	23.5658999
C	22.3568867	21.8475497	26.5388625
C	24.4132618	22.6430676	26.6059954
C	19.9355944	23.7489502	23.5163613
C	20.4139301	22.2589939	25.0562341
C	24.8846124	26.2820713	24.3438189
C	25.5795489	24.5207000	25.4645096
C	20.6351213	27.3867264	22.6975428
C	21.7885371	28.0391866	23.0046316
C	23.0560688	21.1008991	27.5528515
C	24.3282532	21.5868010	27.5947970

C	18.9965474	22.6670392	23.3497756
C	19.2685896	21.7599531	24.3278196
C	26.3256197	26.3483725	24.3703963
C	26.7588818	25.2525078	25.0511136
C	19.8960244	24.9737194	22.8389712
C	24.0588948	27.2601251	23.7610818
C	25.5811192	23.3755602	26.2912692
C	21.0535497	21.5586054	26.0994946
H	19.7376117	27.8074558	22.2547190
H	22.0185732	29.0881795	22.8663367
H	22.6247758	20.3097095	28.1571025
H	25.1216622	21.2635231	28.2621149
H	18.2200344	22.5978239	22.5953767
H	18.7637032	20.8129600	24.4961140
H	26.9248739	27.1211817	23.8994880
H	27.7922888	24.9712126	25.2256846
C	18.9084449	25.2252711	21.7573136
C	24.6981176	28.4948092	23.2033662
C	26.8689839	22.9118388	26.8655071
C	20.2874698	20.4184153	26.6768145
C	19.3792906	25.7364325	20.5329429
C	18.4929869	26.0790560	19.5289346
N	17.1627792	25.8954293	19.6964693
C	17.5223666	25.0146357	21.8826272
C	16.6778979	25.3487582	20.8369308
H	20.4407894	25.8885187	20.3421958
H	18.8479027	26.5188560	18.5956986
H	17.0785166	24.6334517	22.8002502
H	15.5980256	25.2083337	20.8947358
C	19.0124081	20.6445560	27.2419273
C	18.2095311	19.5853324	27.6184394

N	18.6277944	18.3062287	27.4605178
C	20.7205053	19.0799274	26.6129184
C	19.8746006	18.0506740	27.0012143
H	18.6344032	21.6576866	27.3801826
H	17.2091938	19.7241994	28.0347300
H	21.6976884	18.8242315	26.2009947
H	20.1591425	16.9997559	26.9250264
C	27.7871777	23.7779321	27.4977647
C	28.9604989	23.2886586	28.0460105
N	29.2538512	21.9668917	27.9967600
C	27.2326671	21.5490950	26.8100568
C	28.4085707	21.1066743	27.3825279
H	27.5814340	24.8404188	27.6027095
H	29.6812756	23.9357292	28.5503022
H	26.5888633	20.8287771	26.3099878
H	28.7077855	20.0576934	27.3631837
C	24.5918720	28.8159862	21.8419101
C	25.3243652	29.8728272	21.3095278
N	26.1222764	30.6164127	22.0969880
C	25.4921773	29.3387438	24.0042008
C	26.1819290	30.3876154	23.4279476
H	23.9931564	28.2257834	21.1477299
H	25.3319970	30.0880837	20.2382443
H	25.5798395	29.1793919	25.0779095
H	26.8154254	31.0606851	24.0061799
O	23.2095282	23.4260885	23.3914251
O	21.9801116	25.3428677	26.4445098
H	21.0361917	25.5507397	26.3625054
C	27.0415475	31.6043988	21.4955819
H	26.7011721	31.8182544	20.4824681
H	27.0398214	32.5177951	22.1023027

H	28.0391694	31.1454281	21.4409692
C	16.2351217	26.3085722	18.6273084
H	15.2249561	26.3589405	19.0447001
H	16.5281273	27.3004804	18.2653146
H	16.2699026	25.5815305	17.8031131
C	17.6779452	17.2065752	27.7316938
H	16.9265736	17.1774216	26.9308607
H	18.2242449	16.2584019	27.7707930
H	17.1840791	17.3793134	28.6947964
C	30.5111359	21.4700997	28.5880805
H	30.4186248	20.3951358	28.7802602
H	31.3443428	21.6643534	27.8969678
H	30.6876009	21.9933522	29.5356723
H	22.0550413	24.8058194	27.2478247
Fe	22.6858410	24.2392186	24.6777135
C	25.7309237	24.4950412	20.3124874
C	25.7216441	23.0525626	20.0298710
C	26.9188546	22.3403605	20.1381857
C	28.0797465	22.9772766	20.5705025
C	28.0712480	24.3657867	20.9206619
C	26.9279213	25.1110198	20.7785847
H	26.9404576	21.2775507	19.8866137
H	29.0126842	22.4131764	20.6474974
H	28.9828993	24.8417326	21.2809979
H	26.9389310	26.1831559	20.9817833
C	24.4331316	22.3656720	19.6965394
H	24.0848856	21.9219288	20.6551508
H	24.6318245	21.5076795	19.0321428
C	24.5249830	25.3007022	20.0545007
H	24.6673489	25.7609326	19.0401527
H	24.5340938	26.1902050	20.7022807
C	23.3250885	23.2872959	19.1686940
H	22.3777776	22.7270173	19.1501997
H	23.5256720	23.6054831	18.1335682
C	23.2042244	24.5247927	20.0533851
H	22.4195574	25.1859598	19.6684806
H	22.9600904	24.2311828	21.0905935

^{2}TS1-R$_{mod2}$

N	22.1917220	26.5363187	23.6503859
N	21.2815575	23.8962641	23.8418941
N	23.5978992	23.2117648	25.3125883
N	24.6406711	25.8138427	24.8586806
C	20.9662045	26.6792352	23.0445844
C	22.6906218	27.8044953	23.8427152
C	22.7883436	22.1849013	25.7491317
C	24.7724971	23.1023043	26.0082010
C	20.4122248	24.2887302	22.8478120
C	20.9694028	22.5969786	24.1312744
C	24.9724200	27.0874906	24.4686578
C	25.8188166	25.1827659	25.1766394
C	20.6229851	28.0752493	22.9796679
C	21.6830915	28.7701879	23.4837936
C	23.4318111	21.4834218	26.8365494
C	24.6668988	22.0359720	26.9838304
C	19.6327876	23.1570028	22.4031175
C	19.9488542	22.1187272	23.2279842
C	26.4128622	27.2327778	24.4345998
C	26.9307620	26.0460621	24.8621598
C	20.1886128	25.6293763	22.5035978
C	24.0409463	28.0957364	24.1418437
C	25.9034424	23.9321973	25.8255079

C	21.5539688	21.8320385	25.1685745
H	19.6878281	28.4670452	22.5952156
H	21.7841524	29.8411356	23.5963982
H	22.9990857	20.6872394	27.4337749
H	25.4113436	21.7649484	27.7267289
H	18.9408331	23.1440034	21.5665415
H	19.5562409	21.1064245	23.1894269
H	26.9564035	28.0972183	24.0587345
H	27.9797490	25.7738380	24.9164530
C	19.0350291	25.9543878	21.6103675
C	24.5779556	29.4752846	23.9775398
C	27.1785854	23.4710951	26.4236491
C	20.8198361	20.6367698	25.6471192
C	19.1770973	26.7064019	20.4317559
C	18.0548396	27.0121586	19.6671355
N	16.8280602	26.5572725	20.0094282
C	17.7438606	25.4830548	21.9198833
C	16.6702001	25.7778265	21.1044547
H	20.1466937	27.0353296	20.0537679
H	18.1334740	27.5996478	18.7521838
H	17.5577009	24.8824561	22.8044456
H	15.6707713	25.3964274	21.3035680
C	19.4315299	20.7101508	25.9125947
C	18.7304337	19.6092362	26.3586521
N	19.3477792	18.4202579	26.5571761
C	21.4239208	19.3816023	25.8766557
C	20.6766559	18.3081438	26.3343276
H	18.8883882	21.6439363	25.7857366
H	17.6606141	19.6448442	26.5734295
H	22.4812929	19.2201166	25.6708215
H	21.1184768	17.3300798	26.5275385
C	28.0427238	24.3067829	27.1618857
C	29.1410962	23.7815977	27.8238148
N	29.4127955	22.4558232	27.7791897
C	27.5481330	22.1090077	26.3415421
C	28.6390612	21.6301800	27.0367099
H	27.8407082	25.3680822	27.2805632
H	29.8097635	24.4028344	28.4231577
H	26.9657601	21.4126138	25.7422442
H	28.9228870	20.5766735	27.0197195
C	24.3297126	30.2389829	22.8299330
C	24.9563704	31.4648911	22.6521376
N	25.8191653	31.9359936	23.5721959
C	25.4576994	30.0213044	24.9323507
C	26.0715325	31.2344303	24.6987999
H	23.7333165	29.8552056	22.0041327
H	24.8311291	32.0510814	21.7407406
H	25.6696996	29.4991846	25.8646312
H	26.7739779	31.6805133	25.4035167
O	23.7190064	24.4812447	22.7684689
O	22.1233211	25.4178019	26.1797040
H	21.1828368	25.6237694	26.0656991
C	26.5447034	33.1915041	23.3070166
H	27.1826898	33.0497083	22.4242964
H	25.8225567	33.9960743	23.1277765
H	27.1613427	33.4332620	24.1787578
C	15.6531650	26.8512805	19.1592421
H	14.7510563	26.8442551	19.7831361
H	15.7806560	27.8345265	18.6916092
H	15.5669525	26.0814229	18.3788151
C	18.5404298	17.2593987	26.9818464
H	17.7817540	17.0559688	26.2160222

H	19.1949653	16.3916301	27.1128178
H	18.0487293	17.4882962	27.9339516
C	30.5670175	21.9046568	28.5150545
H	30.3578630	20.8624374	28.7839236
H	31.4639408	21.9611751	27.8814348
H	30.7183430	22.4921662	29.4281592
H	22.1562127	24.7094289	26.8399168
Fe	23.0132221	24.8537963	24.2418038
C	25.6757402	27.0862586	19.0976062
C	25.6020158	26.3700011	20.3740314
C	26.8104185	25.9606482	21.0140865
C	28.0269878	26.3521037	20.5198044
C	28.0856516	27.1932147	19.3687973
C	26.9309266	27.5230351	18.6665153
H	26.7440716	25.3647902	21.9265836
H	28.9515327	26.0508340	21.0137033
H	29.0486168	27.5856881	19.0338032
H	26.9904580	28.1754464	17.7927955
C	24.3400553	26.3415094	21.0965260
H	24.4978911	26.9422578	22.0106099
H	24.0948520	25.3445821	21.8371513
C	24.4355617	27.4965141	18.3720511
H	24.0632452	26.6456057	17.7662613
H	24.6598387	28.3147665	17.6743685
C	23.0912190	26.7133307	20.3197268
H	22.2847607	26.9540707	21.0211005
H	22.7534036	25.8439996	19.7317438
C	23.3268119	27.8863331	19.3545506
H	22.4091560	28.1184342	18.8005828
H	23.6366915	28.7913270	19.9034612

^{2}TS1-S$_{mod2}$

N	22.0691008	25.9208673	23.6343638
N	20.9050675	23.4842328	24.3473291
N	23.2358814	22.8667255	25.8794466
N	24.4810567	25.2248254	24.9038167
C	20.8452749	26.0798743	23.0386391
C	22.6663097	27.1633831	23.6151136
C	22.4090274	21.8893657	26.3876740
C	24.4562035	22.7162545	26.4921973
C	20.0494271	23.7733956	23.3007068
C	20.5246053	22.2591687	24.8263140
C	24.8941741	26.3924757	24.2989955
C	25.6161942	24.5953518	25.3528795
C	20.6014653	27.4760909	22.7764645
C	21.7237179	28.1495090	23.1555624
C	23.0932598	21.1751082	27.4354015
C	24.3579750	21.6791983	27.4991224
C	19.1689154	22.6575122	23.0664016
C	19.4357820	21.7369537	24.0349432
C	26.3343951	26.4564149	24.2902914
C	26.7826682	25.3395325	24.9301406
C	19.9684148	25.0300171	22.6864010
C	24.0423196	27.3918433	23.7952159
C	25.6250480	23.4478609	26.1768860
C	21.1306213	21.5657935	25.8963077
H	19.6842295	27.8930555	22.3721866
H	21.9028314	29.2177132	23.1218429
H	22.6610571	20.3898194	28.0468599
H	25.1384005	21.3807973	28.1929094
H	18.4324287	22.5741508	22.2743601
H	18.9652662	20.7657546	24.1589867

H	26.9236500	27.2403143	23.8221677
H	27.8197369	25.0525156	25.0720732
C	18.9605085	25.3170976	21.6302046
C	24.6317835	28.6768767	23.3041154
C	26.9121160	22.9841318	26.7564721
C	20.3573926	20.4230806	26.4578714
C	19.3663890	25.9684269	20.4484968
C	18.4332441	26.3190185	19.4868253
N	17.1253382	26.0159952	19.6533080
C	17.5942283	24.9958863	21.7542960
C	16.7051928	25.3471026	20.7533575
H	20.4070347	26.2216007	20.2430291
H	18.7357285	26.8495417	18.5814467
H	17.1955021	24.5123401	22.6434623
H	15.6396616	25.1221399	20.8177423
C	19.0262552	20.6289279	26.8868504
C	18.2385924	19.5726327	27.2979478
N	18.7205254	18.3064159	27.2988087
C	20.8401138	19.1018209	26.5359788
C	20.0067862	18.0716737	26.9518309
H	18.5945683	21.6279177	26.8933036
H	17.2041149	19.7023421	27.6250930
H	21.8534793	18.8523514	26.2197800
H	20.3390413	17.0328796	26.9926735
C	27.8412667	23.8537180	27.3669804
C	29.0111015	23.3632707	27.9230586
N	29.2909975	22.0381811	27.8980050
C	27.2576299	21.6160850	26.7343891
C	28.4324132	21.1737662	27.3101084
H	27.6480423	24.9202878	27.4523106
H	29.7380686	24.0132493	28.4155282
H	26.6023454	20.8877238	26.2617063
H	28.7176022	20.1206038	27.3139270
C	24.3960618	29.1196100	21.9938641
C	25.0268166	30.2613355	21.5115037
N	25.8516297	30.9725589	22.2980282
C	25.4887220	29.4596004	24.1009741
C	26.0772528	30.5943093	23.5743491
H	23.7640003	28.5618656	21.3040024
H	24.9260776	30.5860552	20.4739324
H	25.6994070	29.1926697	25.1357247
H	26.7533898	31.2257788	24.1510929
O	23.3473188	23.4233121	23.2874129
O	21.9959531	25.3391073	26.3159613
H	21.0323188	25.4369613	26.2636508
C	26.5851944	32.1321890	21.7473577
H	26.4824871	32.1205631	20.6593454
H	26.1621856	33.0536093	22.1705841
H	27.6458450	32.0124682	21.9950582
C	16.1463854	26.4146026	18.6236046
H	15.1498595	26.4269798	19.0770194
H	16.3962540	27.4182919	18.2621854
H	16.1745958	25.6972156	17.7902935
C	17.7997000	17.1972385	27.6290138
H	17.0427052	17.1129174	26.8384153
H	18.3700519	16.2660036	27.7070032
H	17.3088735	17.4038818	28.5877278
C	30.5477401	21.5416644	28.4915066
H	30.4607294	20.4643340	28.6703978
H	31.3829915	21.7468662	27.8062103
H	30.7149246	22.0562143	29.4455394
H	22.1610680	24.8391575	27.1301833

Fe	22.7501868	24.2900494	24.5384110
C	25.7744475	25.2041967	20.3543807
C	25.4130157	23.8284277	20.6709096
C	26.4385693	22.8952638	21.0158133
C	27.7427118	23.3070838	21.1407957
C	28.0649128	24.6793993	20.9388101
C	27.1024211	25.6028969	20.5308290
H	26.1574699	21.8538877	21.1713080
H	28.5328060	22.5946935	21.3894824
H	29.0880853	25.0204602	21.0939960
H	27.3949412	26.6348631	20.3317601
C	24.0203224	23.4307706	20.7168040
H	23.7683782	23.5368412	21.9145670
H	23.8752916	22.3427866	20.6063487
C	24.7319688	26.1126724	19.7868896
H	24.7368361	25.9955565	18.6820446
H	25.0159140	27.1601393	19.9448792
C	22.9945874	24.3191654	20.0280456
H	21.9854057	24.0552282	20.3832742
H	23.0011464	24.1261067	18.9398148
C	23.3247457	25.7866277	20.2905383
H	22.6067664	26.4487273	19.7948071
H	23.2719658	25.9708216	21.3762162
